# Liposomal drug delivery for lung cancer therapy: progress, challenges, and future perspectives

**DOI:** 10.1186/s12943-026-02728-0

**Published:** 2026-07-03

**Authors:** Abulfazl Vatankhah, Sepehr Hoseinzadeh Moghaddam, Mohsen Rahmanian, Sarah Khosropanah, Fatemeh Oroojalian, Prashant Kesharwani, Amirhossein Sahebkar

**Affiliations:** 1 Natural Products and Medicinal Plants Research Center, North Khorasan University of Medical Sciences, Bojnurd, Iran; 2 North Khorasan University of Medical Science, Bojnurd, Iran; 3 Department of Medical Nanotechnology, School of Medicine, North Khorasan University of Medical Sciences, Bojnurd, Iran; 4 Next-Generation Translational Nanomedicine Laboratory, Department of Pharmaceutical Sciences, Dr. Harisingh Gour Vishwavidyalaya (A Central University), Sagar, 470003 Madhya Pradesh India; 5 Biotechnology Research Center, Pharmaceutical Technology Institute, Mashhad University of Medical Sciences, Mashhad, Iran; 6 Centre for Research Impact and Outcome, Chitkara College of Pharmacy, Chitkara University, Rajpura, India; 7 Applied Biomedical Research Center, Basic Sciences Research Institute, Mashhad University of Medical Sciences, Mashhad, Iran

**Keywords:** Liposomal drug delivery, Lung cancer, Multidrug resistance, Targeted therapy, Immunotherapy, Pulmonary delivery

## Abstract

Liposomes, as one of the most established nanocarriers, have been widely employed in lung cancer therapy, which remains the leading cause of cancer-related mortality worldwide. However, the current landscape of liposomal formulations and the principles guiding their rational design remain insufficiently defined. This review provides a comprehensive analysis of how liposomal delivery systems have been engineered to address biological and therapeutic challenges in lung cancer. We analyze liposome-based delivery of chemotherapeutic agents, including formulations that have advanced to clinical trials, with particular emphasis on strategies developed to overcome multidrug resistance (MDR). Surface engineering and functionalization strategies that enable active targeting and enhance therapeutic efficacy are also reviewed. Liposomal platforms for immunotherapy are examined, alongside their application in delivering phytochemicals, nucleic acid therapeutics, and photo-enabled modalities, including photothermal and photodynamic therapies, which are discussed in dedicated sections. Cutting-edge innovations, such as biomimetic liposomes, stimuli-responsive smart systems, and theranostic designs integrating diagnostic and therapeutic functions, are highlighted. Finally, pulmonary delivery of anticancer agents is evaluated, focusing on formulation parameters informed by current liposome-based evidence and broader lessons from nanomedicine. By providing mechanistic insights, design principles, and translational perspectives, this review offers a unified framework for liposome-based platforms in lung cancer therapy.

**Graphical abstract:**

**Figure:**
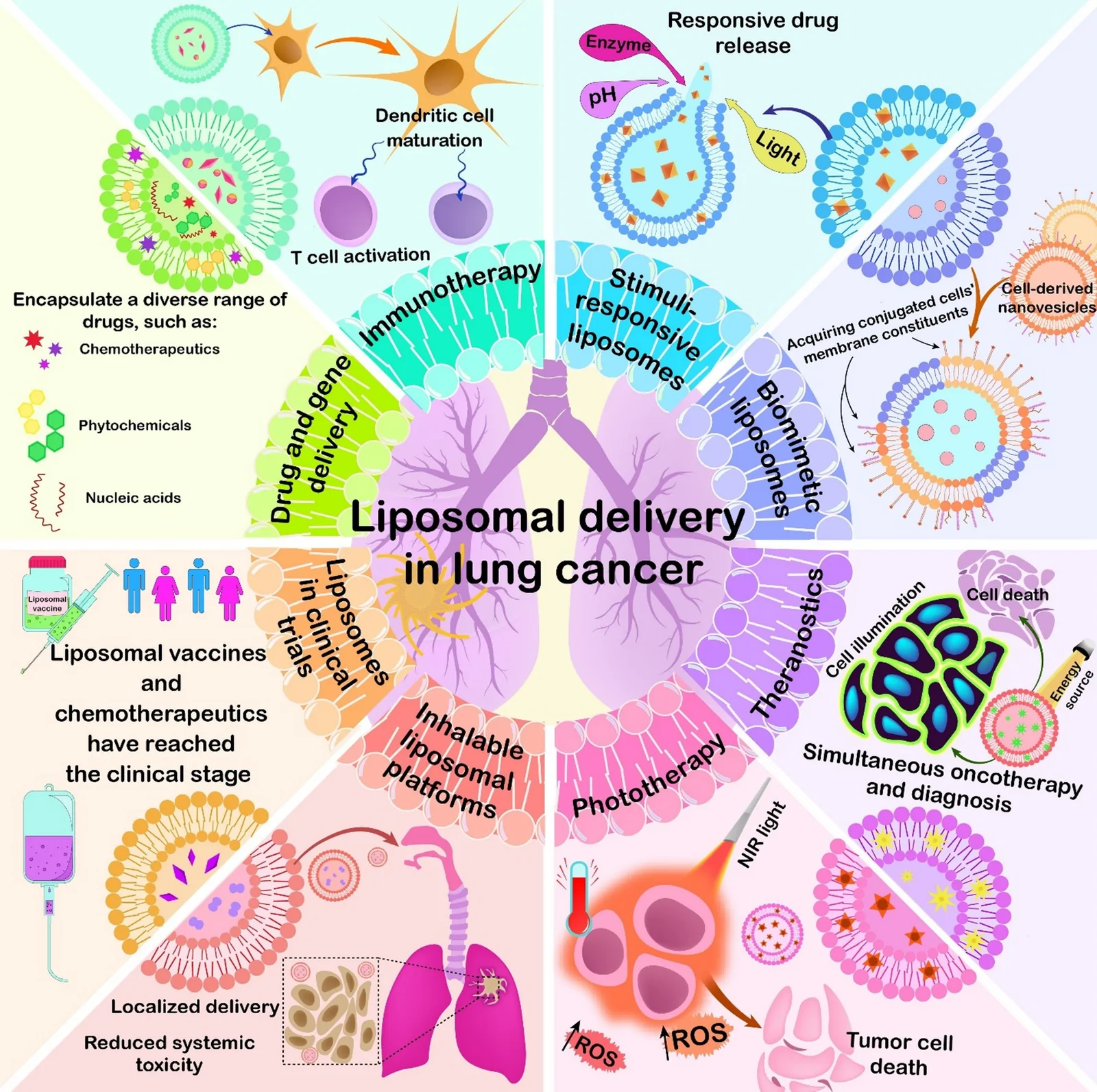

## Introduction

According to estimates from the Global Cancer Observatory (GLOBOCAN) 2022, lung cancer remains the leading cause of cancer-related morbidity and mortality worldwide, with nearly 2.5 million new cases and over 1.8 million deaths annually. Compared to the 2020 estimates, where lung cancer accounted for 2.2 million new cases and 1.8 million deaths, the increase in incidence reveals the growing global burden of this cancer. Accounting for approximately 12.4% of all cancer diagnoses and 18.7% of fatalities worldwide in 2022, lung cancer was the most commonly diagnosed malignancy and the leading cause of cancer mortality globally. Among men, it was the most frequently diagnosed cancer and the leading cause of cancer-related death, whereas among women it ranked second after breast cancer in both incidence and mortality [1, 2].

Lung cancer is primarily classified into two types: non-small cell lung cancer (NSCLC) and small cell lung cancer (SCLC). NSCLC, which accounts for approximately 85% of cases, generally has a better prognosis. NSCLC often involves mutations in key driver genes, such as epidermal growth factor receptor (EGFR), which drive uncontrolled cell growth and, over time, contribute to resistance against targeted therapies [3]. In contrast, SCLC is marked by the inactivation of tumor suppressor genes such as TP53 and RB1, along with the amplification of MYC family genes. These genetic alterations are major factors behind the highly aggressive behavior and significant metastatic potential of SCLC, making it particularly difficult to treat [4, 5]. For NSCLC, treatment options include surgery (for early stages), chemotherapy, and targeted therapies such as EGFR inhibitors, which provide significant but often temporary responses in mutation-positive patients [6, 7]. Moreover, SCLC is frequently diagnosed at an advanced stage, with chemotherapy combined with radiation therapy (and increasingly immunotherapy) remaining the standard treatments, resulting in suboptimal outcomes [8]. While emerging immunotherapies such as pembrolizumab offer some hope, they remain far from being definitive solutions [9].

Despite advances in research and treatment, lung cancer remains one of the deadliest forms of cancer, claiming countless lives every year [10]. Current treatments for lung cancer face numerous challenges, especially since most cases are diagnosed at advanced stages where surgery, chemotherapy, and targeted therapies often fail to achieve a cure [11]. Additionally, lung tumors frequently develop resistance to chemotherapy, with nearly all initially responsive cases eventually becoming resistant through mechanisms like oncogene alterations and activation of survival pathways [12]. Systemic chemotherapy can cause not only general systemic complications but also pulmonary toxicities, such as interstitial lung disease and pneumonitis, which are often challenging to manage with conventional treatments. Similarly, tyrosine kinase inhibitors (TKIs) are associated with these pulmonary toxicities in addition to their characteristic systemic effects [13–15]. While combining chemotherapy with immunotherapy has improved outcomes, it also leads to increased immune-related adverse events (irAEs), such as fatigue, gastrointestinal issues, and endocrine dysfunctions, adding another layer of complexity to treatment regimens [16]. These challenges highlight the unmet need for more targeted and precise therapies to minimize systemic toxicity and ultimately improve patient outcomes.

To address the challenges of conventional chemotherapy and immunotherapy associated with lung cancer treatment, numerous drug delivery systems (DDSs) have been developed, some of which have progressed to clinical trials [17]. Among these, nano drug delivery systems (NDDSs) have emerged as state-of-the-art strategies owing to the well-documented role of nanomaterials and nanostructures in the treatment of various types of tumors [18–22]. Different types of NDDS have been introduced to facilitate the delivery of antitumor agents, thereby significantly enhance their therapeutic outcomes [23–27].

NDDSs protect cytotoxic agents from premature clearance by the mononuclear phagocyte system (MPS) and prevent early degradation or release in circulation. This ensures that the therapeutic agent remains intact and active until it reaches its target site [28]. NDDSs also exploit the leaky vasculature of tumor vessels, a phenomenon known as the enhanced permeability and retention (EPR) effect, to accumulate and be retained within the tumor site. This allows the therapeutic agent to preferentially accumulate in the tumor microenvironment (TME) [29]. Beyond passive targeting through the EPR effect, NDDSs have been functionalized with targeting ligands to achieve active targeting of the tumor site [30]. For lung cancer, which is the primary focus of this review, targeting ligands directed at frequently overexpressed receptors, such as EGFR and the transferrin receptor (TfR), represent well-established strategies [31].

**Fig. 1:**
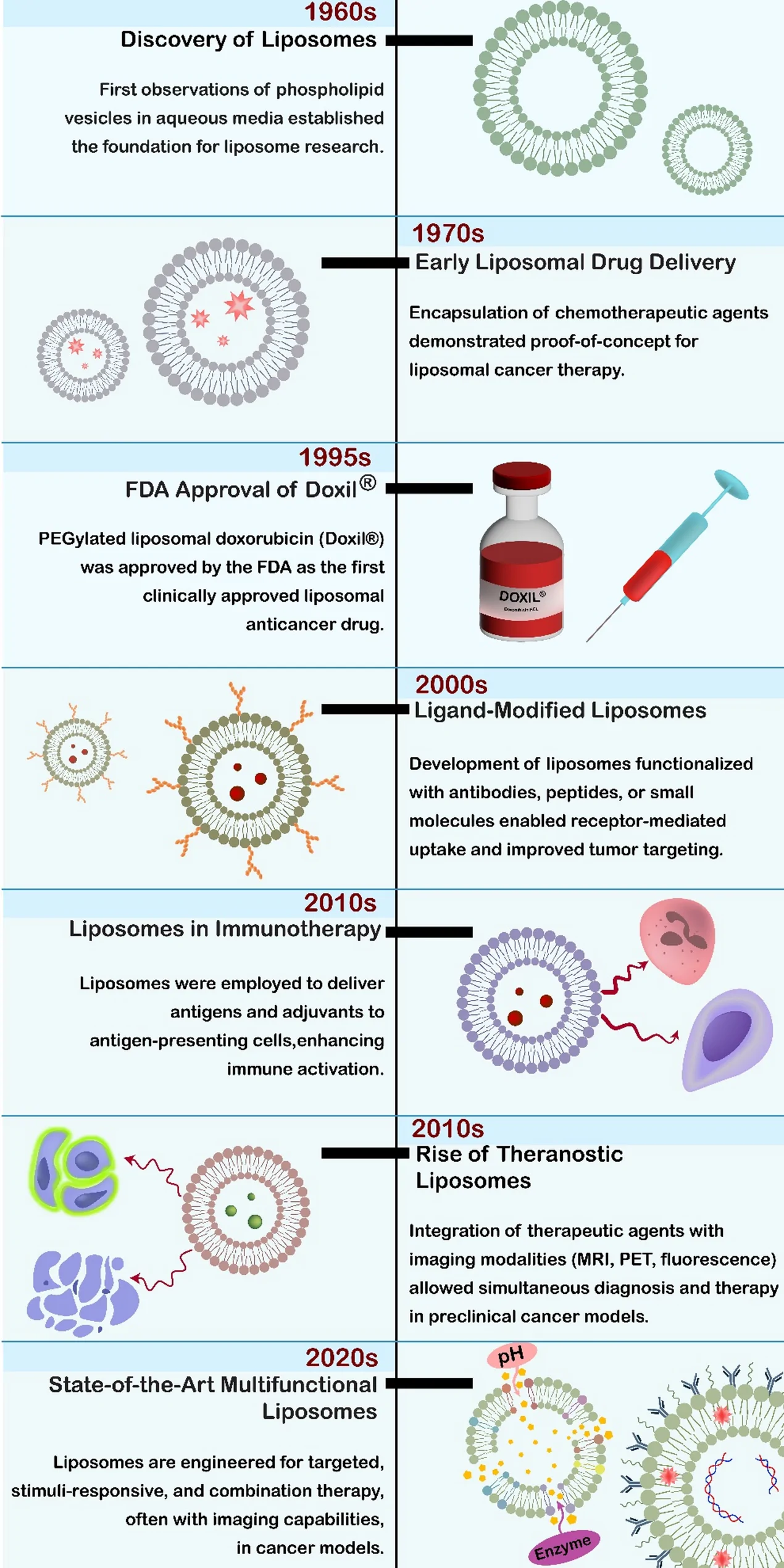
Historical evolution and functional advancement of liposomal DDSs in cancer therapy spanning approximately six decades. Liposomes were first described in the 1960s as model lipid bilayer vesicles and later explored for drug encapsulation and delivery applications, with early studies in the 1970s demonstrating proof-of-concept delivery of chemotherapeutic agents for cancer therapy. This conceptual foundation was translated into clinical development with the approval of PEGylated liposomal DOX (Doxil^®^) in 1995. The following decades saw progressive functionalization, including ligand-mediated active targeting strategies in the 2000s, the development of theranostic platforms in the 2010s, and the emergence of highly engineered multifunctional and stimuli-responsive systems in the 2020s. Current designs are increasingly focused on navigating TME barriers and addressing multidrug resistance, reflecting ongoing progression in precision nanomedicine

Liposomes, first described in the 1960s by Alec D. Bangham and R. W. Horne as phospholipid vesicles with concentric lamellar structures [32], are among the most extensively studied nanocarriers and have been widely investigated for the delivery of chemotherapeutic and immunotherapeutic agents across various cancers, including lung cancer [33]. In one of the foundational studies of liposomal drug delivery, Gregoriadis and Neerunjun demonstrated in 1975 that mice treated with liposome-encapsulated actinomycin D survived longer than those receiving the free drug [34]. This work, together with subsequent early studies on the pharmacokinetics and tissue distribution of liposome-encapsulated anticancer agents, helped establish liposomal drug delivery as a viable therapeutic strategy [35–37]. Liposomes, composed of the same phospholipid components as cell membranes, primarily offered a biocompatible strategy to reduce the systemic toxicity of cytotoxic agents, while also addressing challenges associated with drug targeting to the lungs [38]. Their nanoscale size allowed exploitation of the EPR effect, enabling improved penetration and accumulation within lung tumors [39]. However, the clinical relevance of the EPR effect in human tumors is increasingly considered heterogeneous. EPR-mediated accumulation varies substantially between patients, largely due to differences in vascular architecture, perfusion, and lymphatic drainage, which collectively reduce the predictability of nanocarrier delivery in clinical settings [40, 41]. Additional tumor-to-tumor variability in vascular permeability further limits the reliability of EPR-dependent accumulation [42]. The first liposomal chemotherapy approved by the U.S. Food and Drug Administration (FDA) was liposomal doxorubicin (DOX), marketed as Doxil^®^, introduced in the 1990s [43, 44]. Polyethylene glycol (PEG) was incorporated into the liposomal bilayer to extend circulation time and evade immune recognition. This formulation demonstrated high and selective tumor localization in several clinical trials, achieving significantly greater DOX concentrations in tumor interstitial fluid compared with free DOX, while substantially reducing cardiotoxicity [44], one of the most severe and dose-limiting adverse effects of DOX [45]. Over the past three decades, advances in pharmaceutical sciences have refined liposomal designs to maximize the delivery of cytotoxic agents while markedly reducing adverse effects [46]. The major developmental milestones in liposome research and their progressive translation into cancer therapy are summarized schematically in Fig. 1.

Although liposomal drug delivery has been extensively reviewed within the broader field of cancer nanomedicine, most existing studies adopt a generalized framework that does not systematically address lung cancer as a distinct therapeutic context characterized by unique biological, pharmacokinetic, and translational barriers. Consequently, key limitations specific to pulmonary delivery, TME constraints, and clinical translation in lung cancer are often not integrated into a unified design perspective. To bridge this gap, this review adopts a comprehensive, barrier-centric framework. We first summarize the principal obstacles limiting effective lung cancer therapy, with their mechanistic basis and therapeutic consequences outlined in Table 1. We then discuss rational liposomal design principles, examining how physicochemical properties, PEGylation, targeting strategies, and approaches to overcoming multidrug resistance (MDR) have been engineered to address these lung-specific limitations. Subsequent sections evaluate distinct liposomal platforms for chemotherapy, immunotherapy and cancer vaccines, phytochemicals, nucleic acids, phototherapy, and inhalation-based delivery. Translational challenges are also discussed within their corresponding sections, with dedicated emphasis on clinical studies of liposomal chemotherapy and immunotherapy. Finally, emerging smart designs are examined, including biomimetic and hybrid systems, stimuli-responsive formulations, and theranostics, followed by a dedicated analysis of the current patent landscape and forward-looking perspectives on the trajectory of liposomal lung cancer therapies.

**Table 1:** Major biological, delivery, and translational barriers limiting nanomedicine efficacy in lung cancer

Barrier Type	Mechanistic Basis	Consequence for Treatment
1. Tumor microenvironment and cellular barriers		
Physical	Dense ECM (collagen, hyaluronic acid), CAF-mediated stromal remodeling, elevated interstitial fluid pressure, abnormal vasculature, and impaired lymphatic drainage [47, 48].	Restricts intratumoral transport and tissue penetration, leading to heterogeneous drug distribution and sub‑lethal exposure in poorly perfused tumor regions, which may necessitate higher systemic doses [48–50].
Chemical/Metabolic	Regional hypoxia, extracellular acidosis, and metabolic reprogramming associated with aerobic glycolysis (Warburg effect) [51].	Drives resistance to radiation and conventional chemotherapies, while impairing local immune function [51, 52].
Immunological	Abundant immunosuppressive cells (Tregs, MDSCs, TAMs, CAFs) and upregulated checkpoint ligands (PDL1, TGF‑β, LAG‑3, TIM‑3) [53, 54].	Results in immune evasion and suppression of effector T‑cell activity, promote an immunosuppressive TME, and severely limit the efficacy of immunotherapy in lung cancer [53].
Cellular resistance mechanisms	Overexpression of ABC transporters (e.g., P-gp, BCRP), enhanced DNA repair pathways, and apoptosis resistance mechanisms [55].	Reduces intracellular drug accumulation and promotes tumor cell survival following therapeutic stress, driving multidrug resistance (MDR) and treatment failure [55].
2. Tumor evolution and heterogeneity barriers		
Intratumoral heterogeneity	Intrinsic genetic and phenotypic variability across spatial and temporal tumor regions [56, 57].	Drives differential treatment sensitivity and adaptive clonal selection, increasing the risk of therapeutic resistance and disease relapse [56, 57].
Interpatient heterogeneity	Variability in tumor subtype, molecular drivers, stromal composition, and immune contexture among patients [58, 59].	Results in inconsistent therapeutic responses and complicates patient stratification [58, 59].
3. Pharmacokinetic and Delivery Barriers		
Inhalation Clearance	Entrapment within airway mucus, mucociliary clearance, and rapid uptake by alveolar macrophages [60, 61].	Compromises local pulmonary retention, impedes therapeutic access to the underlying epithelium, and restricts deep pulmonary bioavailability [62].
Systemic Clearance	Plasma opsonization (protein corona) and RES sequestration in liver and spleen [63].	Shortens circulation half-life, restricting the dose reaching tumor site [63].
EPR variability	Intertumoral differences in vascular permeability, perfusion, stromal composition, and vessel maturity [64–66].	Can produce unpredictable nanoparticle accumulation and heterogeneous treatment outcomes [64, 65].
4. Clinical and Translational barriers		
Anti-PEG Immunity	Pre-existing or treatment-induced anti-PEG antibodies and accelerated blood clearance (ABC) phenomenon [67].	May trigger acute infusion reactions (CARPA, even on first dose) and cause rapid systemic clearance upon repeated dosing [67].
Manufacturing Scale-up	Complex nanomedicine formulations are difficult to manufacture reproducibly at industrial GMP scale while maintaining essential quality attributes [68].	Challenges in batch-to-batch consistency, increased production costs, and barriers to clinical translation and commercialization [69].
Regulatory Hurdles	Fragmented international regulatory standards and limited availability of validated testing methods and reference materials [70].	Delays clinical translation, inflates trial costs, and limits the clinical pipeline [71].

*ABC (transporters) *ATP-binding cassette transporters, *BCRP* Breast cancer resistance protein, *CAF* Cancer-associated fibroblast, *CARPA* Complement activation-related pseudoallergy, *ECM* Extracellular matrix, *EPR* Enhanced permeability and retention, *GMP* Good manufacturing practice, *LAG-3* Lymphocyte-activation gene 3, *MDSC* Myeloid-derived suppressor cell, *PDL1* Programmed death-ligand 1, *PEG* Polyethylene glycol, *P-gp* P-glycoprotein, *RES* Reticuloendothelial system, *TAM* Tumor-associated macrophage, *TGF-β* Transforming growth factor-beta, *TIM-3* T-cell immunoglobulin and mucin-domain-containing 3, *TME* Tumor microenvironment, *Treg* Regulatory T cell

## Rational design of liposomal systems to overcome biological barriers

### Physicochemical foundations of liposomal formulations

Structurally, liposomes are formed through the self-assembly of phospholipids, natural and synthetic lipids, steroids (e.g., cholesterol), and surfactants into bilayer vesicles capable of encapsulating hydrophilic and hydrophobic molecules. To establish generalizable rules for rational formulation, it is essential to recognize the cardinal design variables that determine liposomal physicochemical and pharmacokinetic characteristics, including encapsulation efficiency (EE), size, rigidity, surface charge, stability, and drug release kinetics [72, 73]:

(i) Composition of lipid constituents: Strongly influences core characteristics, including particle size, membrane rigidity, colloidal stability, and surface charge [74–80]. Alterations in membrane composition, such as adjusting lipid saturation, cholesterol content, or incorporating membrane-stabilizing moieties, can additionally reduce premature drug leakage and improve EE [81].
(ii) Fabrication method: Different production methods provide distinct advantages regarding size control, EE, scalability, and reproducibility. For example, detergent depletion and microfluidic-based methods provide improved control over particle size distribution, whereas reverse-phase evaporation methods may achieve higher EE [82].
(iii) Solvent selection and injection mechanics: Liposome size is governed by the rate of solvent-buffer mixing. Fast mixing (e.g., ethanol, owing to its low viscosity and high diffusivity, or high flow rate ratios) yields small, monodisperse vesicles, whereas slow mixing (e.g., isopropyl alcohol, due to its higher viscosity and lower diffusivity) produces larger, polydisperse populations. However, this solvent sensitivity is overridden in bilayers containing ≥ 14 wt% PEGylated lipids, where the solvent effect on size is no longer observed [83, 84].
(iv) Size reduction methods and formulation integrity: Extrusion provides mild, reproducible processing but is constrained by limited scalability and product losses. In contrast, high-energy methods such as sonication and high-pressure homogenization enable continuous manufacturing but impose shear stress, cavitation, and thermal effects that can compromise vesicle integrity, induce lipid degradation, and reduce encapsulation stability. Therefore, the rational selection of a downsizing strategy must balance the desired size uniformity against the sensitivity of both the lipid bilayer and the encapsulated cargo [85, 86].
(v) Influence of cargo physicochemical properties: The physicochemical properties of the therapeutic payload dictate the optimal liposomal architecture, as they determine cargo partitioning and retention. For instance, the incorporation of highly lipophilic drugs requires precise structural tuning, such as adjusting cholesterol content [87] or lipid chain length [78], to maximize EE and prevent premature release.
(vi) Loading method: The selection of loading mechanism (active versus passive encapsulation) dictates whether transmembrane gradients are established or exploited, influencing the molecular organization of the cargo and thus governing encapsulation capacity, initial liposome size, and drug release kinetics [88].
(vii) Lipid-to-drug ratio: This ratio dictates the balance between loading capacity and structural stability. Higher lipid-to-drug ratios enhance EE and bilayer integrity, whereas lower ratios (excess cargo) often exceed the bilayer’s structural capacity, inducing premature leakage and reduced EE. Therefore, formulation development must identify the optimal ratio that balances these competing factors [89, 90].
(viii) Lamellarity: Determines internal aqueous volume and bilayer organization; unilamellar and multivesicular vesicles generally provide higher hydrophilic drug loading capacity, whereas multilamellar structures favor lipophilic cargo entrapment and also influence drug release kinetics [91].

The ultimate physicochemical attributes of a formulation are determined by these interconnected fabrication variables. Among these, lipid composition remains the primary determinant of membrane behavior, fundamentally influencing bilayer fluidity, permeability, rigidity, and surface charge. These optimized physicochemical properties, in turn, directly dictate liposomal behavior, distribution, fate, and therapeutic efficiency:

#### Size

Liposome size, ranging from 5 nm to over 1000 nm [92], is a fundamental determinant of pharmacokinetic behavior. While tumor extravasation via the EPR effect is possible for vesicles within the 40–200 nm range [93], optimizing a formulation for successful delivery requires navigating a narrower set of constraints. Particle size influences circulation time, reticuloendothelial system (RES) uptake, and intracellular fate [94, 95]. A clear design trade-off exists: while keeping liposomes under 100 nm minimizes RES uptake and extends circulation, this size also results in passive accumulation in the liver and spleen through endothelial fenestrations [96]. Conversely, size-dependent internalization mechanisms demonstrate that larger particles are more prone to macrophage-mediated phagocytosis and elicit significantly higher pro-inflammatory cytokine responses, such as interleukin (IL)-1β and tumor necrosis factor-alpha (TNF-α), compared to smaller counterparts [97].

Furthermore, size affects cellular internalization routes, a critical consideration for evading lysosomal degradation. While classical biology defines rigid size limits for distinct endocytic pathways [98], these thresholds can shift depending on the target cell type and the nature of the delivery vehicle. For example, in targeted populations like dendritic cells (DCs), smaller cationic liposome/DNA complexes (~ 270 nm) effectively bypass the lysosome by utilizing caveolae-mediated entry, whereas larger complexes are predominantly internalized via clathrin-mediated pathways, routing them toward lysosomal digestion [94]. Ultimately, size governs pharmacokinetics, tissue distribution, and intracellular fate across multiple biological scales. Optimization therefore requires balancing systemic exposure, RES clearance, and tumor accumulation mechanisms that may involve EPR-related transport in solid tumors. Accordingly, size functions as a context-dependent design variable rather than a fixed target, tuned to overcome specific barriers from systemic clearance to intracellular trafficking.

#### Surface charge

Liposomes can be engineered to possess a cationic, anionic, or neutral surface charge depending on the choice of lipid composition [99]. The surface charge is a decisive design parameter that affects therapeutic performance from injection to the target site, modulating colloidal stability, systemic protein adsorption, and circulation kinetics, while simultaneously governing tumor-cell internalization and vascular interactions. While a high-magnitude zeta potential is traditionally viewed as a reliable indicator of stability, electrostatic repulsion is heavily suppressed in physiological environments. Due to charge shielding by increased ionic strength, bare charged liposomes rapidly aggregate within 24 h in physiological buffers [100]. Surface charge also governs the composition of the protein corona formed on liposomes. Specifically, Van der Sanden et al. demonstrated that the biomolecular corona varies distinctly with surface chemistry: cationic liposomes exhibit a marked enrichment of vitronectin, anionic liposomes preferentially enrich immunoglobulin M (IgM) fragments, and near-neutral PEGylated liposomes demonstrate an increased abundance of apolipoproteins and albumin [101]. As repeatedly evidenced, cationic nanoparticles (NPs) are taken up more efficiently by nonphagocytic cells, including many tumor cell lines, than anionic or neutral NPs, a property attributed to the negative charge of the plasma membrane [102]. However, the trade-offs associated with positively charged liposomes should be meticulously considered; highly positive formulations bind aggressively to angiogenic tumor endothelium and the peripheral extracellular matrix (ECM), impeding deep diffusion into the tumor parenchyma. Conversely, neutral or less cationic formulations exhibit deeper interstitial penetration [103, 104].

#### Rigidity

Although customizing the rigidity and deformability of NPs remains less explored than size or charge, the inherent contradictions and trade-offs are equally apparent. While rigid liposomes often exhibit lower drug EE, they are less prone to lipid transfer and destabilization by lipoproteins compared to highly fluid liposomes [105]. Furthermore, elasticity modulates intracellular fate: Guo et al. specifically demonstrated that soft nanolipogels (~ 45 kPa) are taken up by cancer cells predominantly via direct membrane fusion, ensuring cytosolic delivery, whereas stiffer counterparts (~ 19 MPa) are internalized via clathrin-mediated endocytosis and become trapped in endosomes [106]. Wu et al., however, determined that moderately rigid liposomes (28.2 ± 3.4 MPa) perfectly balance two opposing biophysical requirements: they are sufficiently deformable to navigate the dense interstitial stroma, yet rigid enough to trigger efficient cellular uptake [107]. Perceived discrepancies in optimal rigidity might stem from the experimental models employed. As Hui et al. demonstrated, 2D in vitro studies can yield misleading outcomes; in contrast, physiologically relevant 3D spheroids and in vivo tumor models reveal that higher deformability facilitates deeper interstitial penetration and superior overall tumor accumulation [108].

Given these competing biophysical requirements in tuning the physicochemical profile of liposomes for optimal tumor delivery, the rational application of surface coatings, such as PEGylation, and active targeting has become indispensable to preserve these properties during systemic circulation.

### PEGylation: evolution, benefits, and limitations

Systemically administered NDDSs are often cleared prematurely due to the body’s immune defenses, reducing their accumulation at the intended site of action. Surface modification of liposomes with stealth coatings, most commonly PEG, allows carriers to maintain prolonged circulation, evade immune recognition, and improve therapeutic outcomes [109]. In cancer therapy, this approach, widely referred to as PEGylation, has proven particularly valuable for extending circulation time and minimizing premature clearance by the immune system, thus increasing the likelihood of tumor accumulation of the NDDS. Additionally, PEGylation has been shown to mitigate the systemic toxicity of NPs and confers formulations with more favorable safety profiles [110, 111]. Such success has enabled PEGylated liposomal formulations to advance into human phase trials. Several trials have utilized PEGylated liposomal formulations for treating patients with lung cancer [112].

**Fig. 2:**
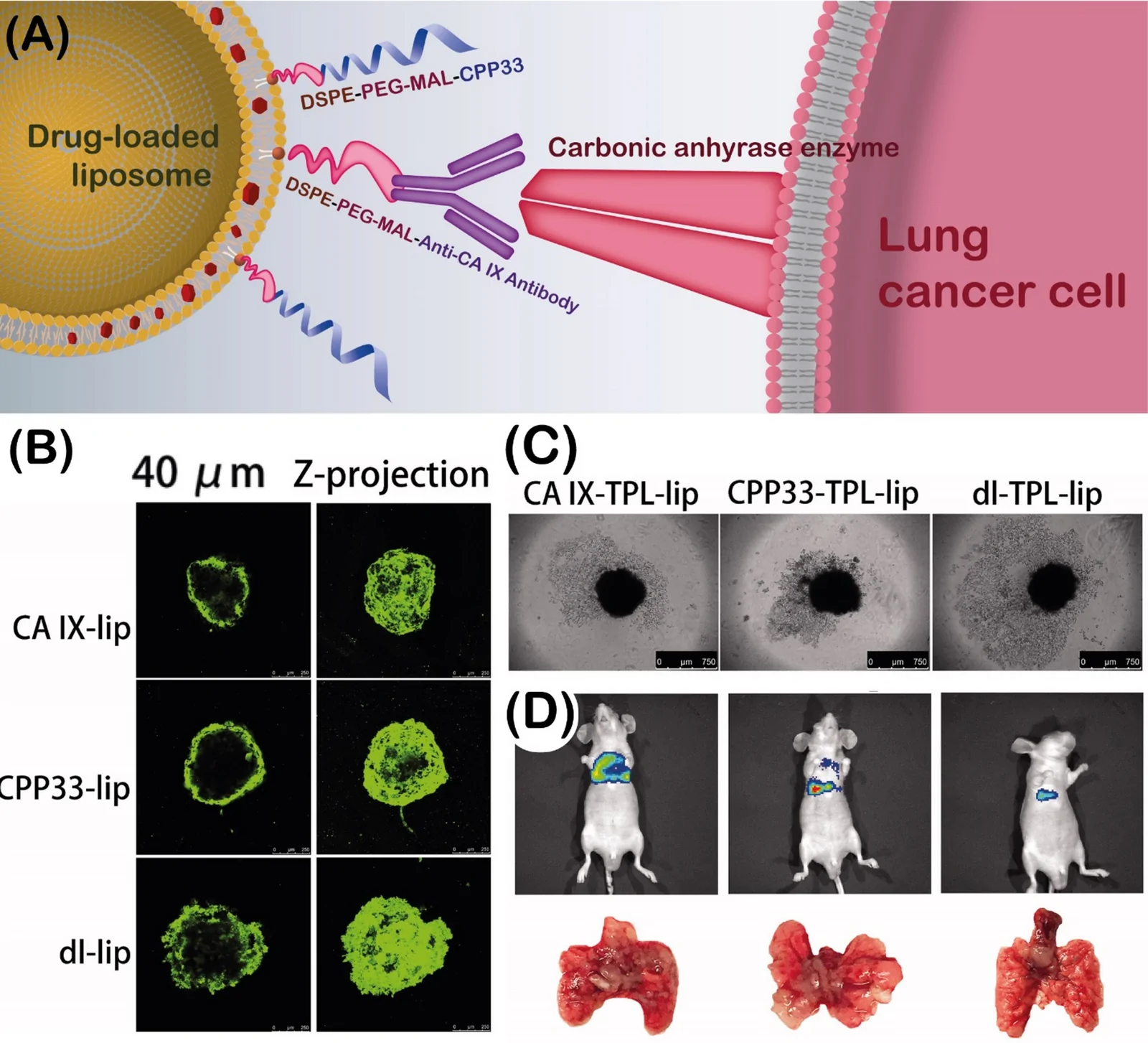
**A** Dual-ligand-modified liposomes incorporating DSPE-PEG-MAL-anti-CA IX antibodies for specific targeting of lung cancer cells and DSPE-PEG-MAL-CPP33 for enhanced tumor penetration. **B** Confocal laser scanning microscopy images demonstrating penetration of CA IX-lip, CPP33-lip, and dl-lip (dual-ligand-modified liposomes) into A549 3D tumor spheroids at 40 μm depth and Z-projection. **C** Brightfield microscopy images showing the structural collapse of A549 3D tumor spheroids after 4 days of treatment with CA IX-TPL-lip, CPP33-TPL-lip, and dl-TPL-lip. **D** Bioluminescence images and corresponding dissected lungs from an orthotopic lung cancer mouse model treated with CA IX-TPL-lip, CPP33-TPL-lip, and dl-TPL-lip, highlighting the strongest anti-cancer effect for dl-TPL-lip. Abbreviations: DSPE-PEG-MAL, distearoylphosphatidylethanolamine-polyethylene glycol-maleimide; CPP33, tumor lineage-homing cell-penetrating peptide; TPL, triptolide. Adapted and reillustrated with permission from ref [115]. Distributed under the terms of the Creative Commons Attribution 4.0 International License (CC BY 4.0)

PEGylation increases the surface hydrophilicity of liposomes, forming a dense corona that sterically hinders interactions with cell membranes and reduces cellular internalization [113]. Accordingly, cell-penetrating peptides (CPPs) have been integrated into PEGylated nanocarriers to restore intracellular delivery efficiency [114]. For example, Lin et al. modified the PEGylated liposomes with two ligands: an anti-carbonic anhydrase IX (CA IX) antibody, which specifically targets the CA IX protein overexpressed on hypoxic NSCLC cells (Fig. 2), and CPP33, a CPP that enables deeper penetration into NSCLC A549 cells. These dual-ligand-modified liposomes achieved active targeting of NSCLC cells, minimized off-target toxicity, and demonstrated superior penetration into tumor masses compared to liposomes lacking CPP33, as evidenced by 3D spheroid models [115]. In another study, PEGylated liposomes were functionalized with CB5005, serving two purposes. In addition to functioning as a CPP, CB5005 inhibited the nuclear translocation of nuclear factor-κB (NF-κB) proteins, a transcription factor that promotes antiapoptotic gene expression and drug resistance in tumor cells. As expected, CB5005-modified liposomes consistently outperformed unmodified formulations by exhibiting higher cellular uptake in A549 cells, inducing greater levels of apoptosis, and overall achieving stronger antitumor efficacy [116].

#### Immune recognition and its consequences

Although most studies on PEG-related in vivo behavior, including anti-PEG antibody formation and immune activation, have not been conducted specifically in lung cancer models, the systemic fate of liposomes is governed by these processes and therefore remains directly relevant to lung-targeted delivery. PEG is generally considered weakly immunogenic in its free form but can become immunogenic when presented in association with macromolecular carriers such as nanocarriers, sometimes acting as a hapten [117]. In this context, the immunogenicity of PEGylated nanocarriers (e.g., liposomes) refers to the induction of anti-PEG antibodies, predominantly IgM, but also IgG, IgE, and IgA. This response is initiated when PEG presented on the NP surface cross-links B-cell receptors (BCRs), triggering a T-cell-independent IgM response primarily mediated by splenic marginal zone B cells. Unlike free PEG chains, PEG on nanocarriers is displayed in a multivalent and highly repetitive configuration, which promotes BCR cross-linking [118]. Antibody induction per se does not equate to immune activation. Once anti-PEG antibodies are generated, their binding to PEGylated liposomes generates anaphylatoxins (C3a, C5a) and leads to opsonization with C3 fragments, marking the liposomes for rapid clearance by Kupffer cells and other phagocytes [119].

The immune activation triggered by PEGylated liposomes can be divided into two outcome domains: pharmacodynamic and pharmacokinetic alterations, which affect the in vivo performance of the nanocarrier, and hypersensitivity reactions, which pose direct safety risks to patients.

##### Pharmacokinetic alterations: the accelerated blood clearance (ABC) phenomenon

Anti-PEG antibodies alter the pharmacokinetics and, consequently, the pharmacodynamic performance of the cargo, primarily through accelerated systemic clearance. The accelerated blood clearance (ABC) phenomenon is a well-established effect in preclinical models and, to a lesser extent, as observed in both preclinical and clinical settings [120]. Upon repeated administration, anti-PEG IgM antibodies bind to PEGylated liposomes with high avidity through their multiple antigen-binding sites. This binding triggers the classical complement pathway, leading to deposition of C3b fragments on the liposome surface and subsequent opsonization. Opsonized particles are then rapidly cleared by the MPS, mainly Kupffer cells in the liver and macrophages in the spleen [121]. Anti-PEG IgG may also contribute to clearance, although its efficiency is generally lower than that of IgM, likely due to IgM’s pentameric structure providing more binding sites and enabling more extensive cross-linking of PEG epitopes displayed on large vesicular systems such as PEGylated liposomes [122]. Rapid clearance reduces systemic exposure, shortens circulation half-life, and decreases tumor accumulation. In humans, accelerated clearance is less evident and not consistently reproducible across studies, reflecting interspecies differences in immune and complement function. The immune landscape is more strongly influenced by pre-existing anti-PEG antibodies, predominantly IgG. Moreover, repeat-dose clearance effects are less consistently observed, while infusion-related hypersensitivity reactions are more frequently reported in clinical settings [123]. Anti-PEG antibodies have also been shown to activate the complement system, leading to the formation of the membrane attack complex and premature cargo release from liposomal systems, ultimately altering the efficacy and safety profile of the drug [124].

##### Hypersensitivity reactions

Hypersensitivity reactions arise from the same upstream immune recognition events but manifest as acute infusion-associated clinical outcomes. The dominant mechanism is complement activation-related pseudoallergy (CARPA), in which anti-PEG antibodies (primarily IgM) form immune complexes on the liposomal surface, triggering the classical complement pathway and generating anaphylatoxins C3a and C5a. These anaphylatoxins stimulate mast cells and basophils, leading to rapid release of vasoactive mediators, including histamine, platelet-activating factor, and leukotrienes [125]. In addition, anti-PEG IgG immune complexes can engage Fcγ receptors on innate immune cells, further amplifying mediator release [126]. Anti-PEG IgE has been reported only in rare cases, and its role in PEGylated NP-associated reactions remains uncertain and non-dominant [127]. Real-world pharmacovigilance data show that hypersensitivity reactions are reported more frequently with PEGylated formulations than with non-PEGylated comparators; however, the vast majority are mild to moderate, and severe anaphylaxis remains uncommon [128]. Clinical data from PEGylated liposomal DOX also indicate that infusion reactions occur in a minority of patients when appropriate premedication is used, decreasing from up to 45% to approximately 4–7%. Overall, hypersensitivity reactions to PEGylated products are clinically relevant but typically manageable with premedication (e.g., antihistamines and corticosteroids), and slow infusion rates [129].

#### Alternative stealth coatings

A large body of research has explored factors influencing PEG immunogenicity and approaches to mitigate it. PEG engineering strategies, including modulation of physicochemical parameters such as surface PEG density [130] and molecular weight [131], structural modifications such as cleavable PEG [132], and administration-related approaches such as slow infusion [133], dosing frequency adjustment [134], and PEG pretreatment [135], have been proposed and reviewed elsewhere [121, 136]. Because the present focus is on alternative coating materials, readers are referred to these recent reviews for comprehensive coverage of PEG engineering and dosing optimization strategies.

##### Zwitterionic polymer coatings

There is growing interest in alternative surface coating strategies to overcome the limitations of PEGylation, particularly the induction of anti-PEG antibodies and the ABC phenomenon. Among these, zwitterionic polymers have attracted attention due to their excellent antifouling properties— that is, their ability to resist the unwanted adsorption of proteins [137]. Unlike PEG, which binds water primarily through hydrogen bonding, zwitterionic polymers carry both positive and negative charges on the same monomer unit, resulting in an overall charge-neutral structure. They bind water via ionic solvation (ion-dipole interactions), creating a superhydrophilic surface with a dense hydration layer that resists nonspecific protein adsorption and cellular interactions [138]. In particular, polycarboxybetaines (PCBs) and poly(2-methacryloyloxyethyl phosphorylcholine) (PMPC) have been investigated as surface coatings with respect to their resistance to nonspecific protein adsorption and their in vivo pharmacokinetic and immunological profiles.

PCBs have been evaluated in several nanocarrier systems for their in vivo behavior. In a comparative study of PEGylated and PCBylated liposomes, Li et al. showed that PEGylated lipoplexes delivering anionic small interfering RNA (siRNA) induce anti-PEG IgM production and accelerated blood clearance upon second administration, resulting in reduced circulation time and increased hepatic uptake. In contrast, PCB-modified lipoplexes showed minimal anti-PCB IgM induction and did not exhibit accelerated clearance after repeated dosing, indicating attenuation of antibody-mediated clearance mechanisms [139]. IgG was additionally assessed to capture longer-term humoral immune responses beyond the initial IgM-driven accelerated clearance, providing further insight into adaptive immunogenicity under repeated administration. Similar suppression of antibody formation and preservation of repeat-dose circulation have also been observed in a gold NP system, supporting broader generalizability across nanocarrier platforms [140]. However, a subsequent study indicates that PCBylation cannot be considered universally biologically inert. Spacer length critically influences both pharmacokinetics and immunogenicity. PCB1 (single-carbon spacer) prolonged circulation at 10–15 mol% grafting density and, under some conditions, outperformed PEGylated liposomes in blood retention. By contrast, PCB3-modified (three-carbon spacer) was rapidly cleared from circulation. Importantly, both PCB1- and PCB3-modified liposomes induced specific anti-PCB IgM and triggered the ABC phenomenon after repeated administration. At higher doses, IgM induction was attenuated, indicating a dose-dependent tolerance effect rather than true absence of immunogenicity [141]. Furthermore, in another study, PCB-coated shell crosslinked knedel-like NPs induced greater cytokine release than their PEG-coated counterparts, indicating possible higher immunotoxicity [142].

Another coating alternative is PMPC, which is widely recognized for its antifouling properties and is already used in clinical biomedical devices [143]. PMPC-conjugated liposomes exhibit excellent stability against aggregation, comparable to PEGylated liposomes, with no aggregation observed over one month of storage or five days in the presence of serum albumin [144]. In vitro, Suzuki et al. reported that PMPC-modified liposomes adsorbed much less serum protein than PEGylated liposomes (~ 0–3 µg total protein vs. >600 µg for PEG) and were not recognized by an anti-PEG IgM antibody; even a short 3.7 kDa PMPC chain (DP10) was as effective as longer chains when incorporated at high density (9.5 mol%). However, these data rely on bulk protein adsorption assays without full protein corona characterization and lack in vivo validation, limiting conclusions on immune recognition and circulation [145]. In contrast, Kluzek et al. demonstrated in vivo that 20 kDa PMPC-coated liposomes (5 mol%) attenuated the ABC phenomenon and reduced IgM induction compared to PEGylated liposomes, an effect attributed to the highly hydrated zwitterionic interface. Nevertheless, accelerated clearance was not eliminated (ABC index 0.85 vs. 0.55 for PEG), indicating only partial reduction of the phenomenon, and a 2 kDa PMPC at the same density failed to prevent protein-mediated aggregation [146]. These studies indicate that effective protein resistance depends on a combined threshold of polymer chain length and surface density, which governs both colloidal stability and downstream immune recognition. Successful translation of zwitterionic coatings will therefore require precise optimization of polymer architecture and grafting parameters.

It should be noted that while PEGylation has undergone extensive optimization and validation for in vivo applications, alternative polymers such as PCB and PMPC remain comparatively underexplored. Key parameters, including polymer subtype, chain length, and grafting density, are not yet systematically defined. This relative lack of in vivo and translational data currently limits their progression compared to PEG-based systems.

##### Ganglioside-based biomimetic coatings

Natural materials have also been explored as alternatives [147]. Among these, gangliosides are amphiphilic glycosphingolipids naturally present in cellular membranes, consisting of a hydrophobic ceramide tail and a hydrophilic glycan headgroup containing sialic acid (SA) [148]. Incorporation of gangliosides has been shown to reduce serum protein adsorption, suggesting decreased susceptibility to protein corona formation compared to ganglioside-free lipid NPs. This stealth effect extends beyond simple surface shielding and has been attributed in part to electrostatic repulsion mediated by negatively charged SA residues [149]. In addition, terminal SAs can engage sialic acid-binding immunoglobulin-type lectins (Siglecs), contributing to immune modulation and potentially influencing receptor-mediated cellular interactions [150, 151]. In another study, insertion of different ganglioside subtypes into PEGylated liposomes suppressed anti-PEG IgM production and mitigated the ABC phenomenon upon repeated dosing. This effect required co-localization of PEG and gangliosides within the same membrane and was attributed, at least in part, to CD22 (Siglec-2)-mediated B-cell tolerance [152]. However, several limitations exist. PEGylated liposomes are far more clinically established and regulated, placing ganglioside-based systems at a comparatively earlier translational stage [153]. Moreover, Grad et al. suggested that structural changes (disk formation, i.e., lipodisks) may underlie the reported immune effects, as high ganglioside concentrations can alter liposome architecture and confound earlier interpretations [154].

##### Biomimetic immune evasion via cell membrane coatings

Beyond synthetic polymers, biologically derived membrane coatings offer biomimetic immune-evasive strategies. Erythrocyte-, platelet-, and macrophage-derived membranes reduce immune activation and prolong circulation. Furthermore, coating with cancer cell-derived membranes of tumor origin enables homotypic targeting to tumors of the same cellular lineage [155]. Because these systems represent a broader biomimetic design paradigm, their mechanisms and translational implications are discussed in the Biomimetic and Hybrid Liposomal Carriers section.

### Active targeting strategies

#### Mechanisms of ligand-mediated targeting

Although nanomedicine has traditionally relied on the EPR effect for passive tumor targeting, its inherent limitations, including nonspecific biodistribution and inconsistent tumor accumulation across different tumor types, have driven the design of actively targeted NPs [156, 157]. Active targeting via ligand functionalization enables greater precision in anticancer drug delivery [150]. Ligand-functionalized liposomes reinforce anticancer therapy by conjugating moieties such as antibodies, peptides, aptamers, and small molecules to specifically bind to receptors commonly overexpressed on lung cancer cells, such as EGFR, carbonic anhydrase, and human epidermal growth factor receptor 2 (HER2) [158]. These ligands can be covalently conjugated to the lipid components of liposomes through direct conjugation methods, such as amide bond formation and thiol-maleimide coupling, or via different linkers such as peptide linkers [159].

Conjugated ligands facilitate receptor-mediated endocytosis of liposomes, enabling selective tumor cell internalization [160]. For example, Fu et al. fabricated liposomes functionalized with arginine-glycine-aspartate (RGD) peptides to target integrin αvβ3 receptors on lung tumor cells. In vitro, the RGD-modified liposomes showed heightened cellular uptake via active targeting. In vivo, they demonstrated improved tumor accumulation and superior tumor growth inhibition. Unlike free DOX, which was associated with death in all mice before day 9 (attributed to DOX cytotoxicity), all liposome-treated groups survived the full 15-day study period with minimal organ toxicity (no histopathological changes in heart, liver, kidney, or spleen) and weight loss comparable to the saline control group [161].

Folate modification is an example of active targeting. Folate-modified liposomes co-delivering cisplatin (Cis) and miR-219a-5p (a microRNA that reverses Cis resistance) were developed to target folate receptors overexpressed on A549 cells. This folate-mediated targeting enhanced cellular uptake in A549 cells, which ultimately resulted in reversed Cis resistance, inhibited proliferation and migration, and promoted apoptosis in vitro [162]. Another study demonstrated the clinical significance of folate targeting in lung cancer, as the analysis of 184 patients revealed that high folate receptor β (FRβ) expression in tumor-associated macrophages (TAMs) correlated with poorer survival (median overall survival: 33.5 months for high FRβ vs. 81 months for low FRβ). To overcome the limitations of non-specific liposome delivery, researchers developed folate-modified liposomes loaded with a BIM-S plasmid to target FRα-positive tumor cells and FRβ-positive TAMs. In vivo, these liposomes reduced tumor growth, depleted TAMs, reprogrammed the TME, and promoted apoptosis with minimal systemic toxicity. This study demonstrates how specific ligands, such as folate, can enable liposomes to effectively target both cancer cells and TAMs [163].

Mucin-1 (MUC1) is a key surface glycoprotein, markedly overexpressed in NSCLC, particularly in lung adenocarcinoma, making it an attractive target for ligand-mediated drug delivery and emerging immunotherapeutic strategies [164]. Yang et al. functionalized Cis-loaded liposomes with peanut agglutinin (PNA). The PNA-modified liposomes exhibited a 1.24-fold higher uptake in MUC1-positive A549 cells compared to unmodified liposomes, without showing increased uptake in MUC1-negative H460 cells, thus confirming their tumor selectivity. The actively targeted formulation increased apoptosis by 1.5-fold relative to unmodified liposomes in vitro, and achieved an 83.1% tumor growth inhibition rate in vivo, while reducing systemic toxicity [165].

**Fig. 3:**
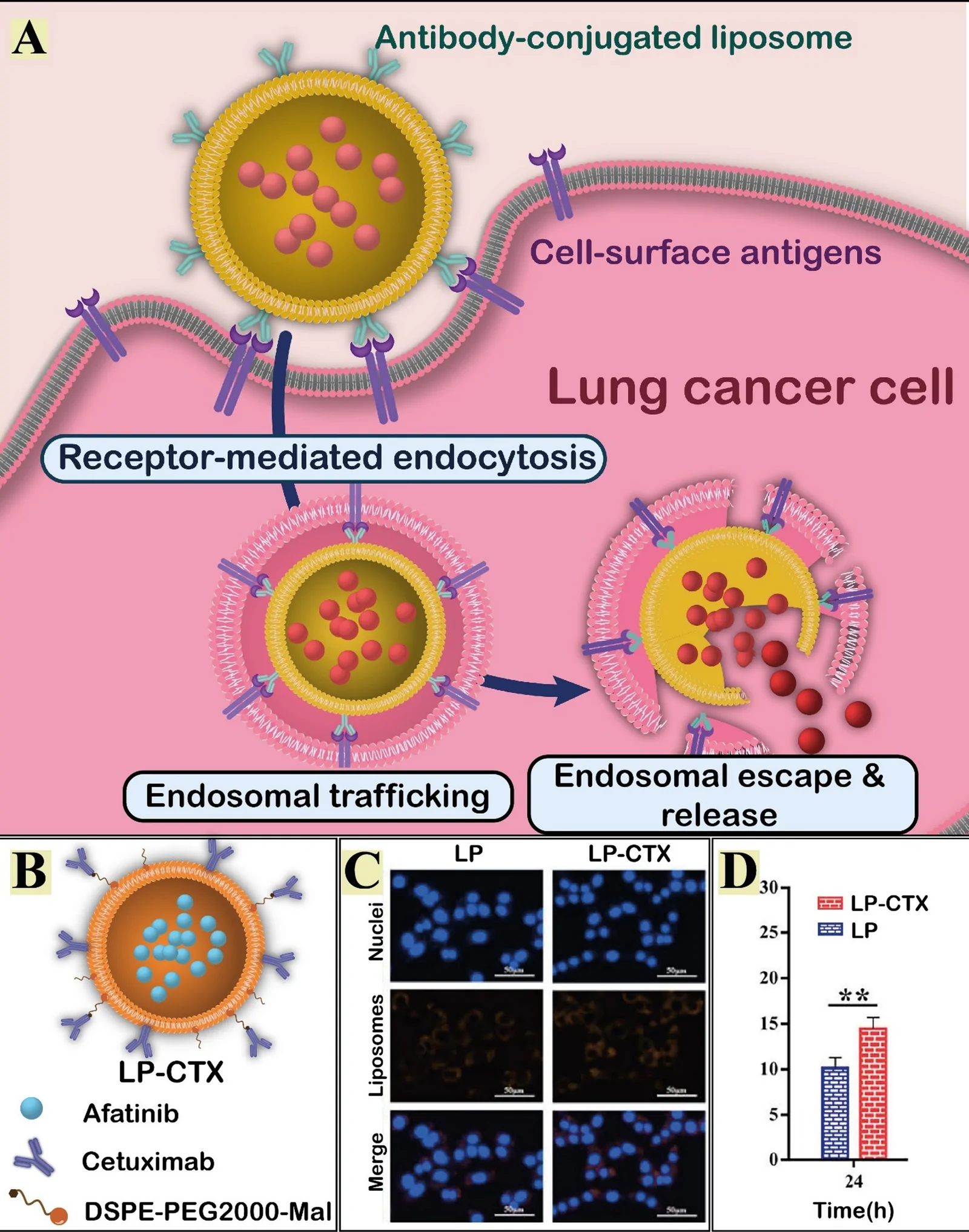
**A** Schematic illustration of antibody-conjugated liposomes (immunoliposomes) targeting cell-surface antigens on lung cancer cells to trigger receptor-mediated endocytosis. After internalization, the liposomes traffic to endosomes, where intra-endosomal destabilization releases the encapsulated therapeutic agent into the cytoplasm to exert anticancer effects. **B** Composition of LP-CTX (cetuximab-conjugated liposomes), incorporating AFT (afatinib) and functionalized with DSPE-PEG2000-Mal (distearoylphosphatidylethanolamine-polyethylene glycol 2000-maleimide) and CTX. **C** Fluorescence microscopy images showing uptake of LP-CTX and LP (non-targeted liposomes without antibody conjugation) in A549 cells at 24 h post-treatment (blue: nuclei; red: liposomes; scale bar = 50 μm). **D** Quantified relative internalization at 24 h, demonstrating significantly higher uptake of LP-CTX in A549 cells (**p < 0.01). Panel (**A**) was created by the authors; panels (**B**-**D**) were adapted and rearranged with permission from ref [170]. Copyright 2019 Elsevier

A highly specific active targeting for tumor sites is to functionalize liposomes with antibodies or antibody fragments, also referred to as immunoliposomes. At the tumor site, antibody-conjugated liposomes undergo receptor-mediated endocytosis, enabling cell-specific uptake (Fig. 3A) [166]. Antibody-mediated targeting partially overcomes passive targeting limitations, such as nonspecific distribution and poor cellular uptake, enabling selective drug delivery to tumors and minimizing systemic toxicity [167]. Additionally, compared to other targeting ligands, immunoliposomes offer superior specificity due to the high affinity and diversity of available antibodies for validated tumor antigens, as well as enhanced avidity from multivalent antibody display on the liposome surface [168]. Targeting EGFR-overexpressing lung tumor cells with anti-EGFR antibodies is one of the well-established treatment strategies in lung cancer therapy [169]. Anti-EGFR immunoliposomes have shown this advantage. Lu et al. fabricated anti-EGFR immunoliposomes by conjugating cetuximab (CTX) to afatinib (AFT)-loaded liposomes (termed LP-CTX) to target EGFR-overexpressing NSCLC cell lines, specifically A549 and H1975. The active targeting enabled the LP-CTX liposomes to achieve greater cellular uptake, compared to unmodified liposomes, by specifically binding to the overexpressed EGFR (Fig. 3B, C, and D) [170]. Although immunoliposomes hold great conceptual promise, their clinical translation remains limited primarily due to restricted tumor penetration and formulation complexity [168].

#### Design principles for clinical translation

Despite preclinical efficacy, the clinical translation of targeted NDDSs is proceeding at a much slower pace. While several actively targeted platforms have reached clinical trials, current FDA-approved NDDSs rely only on passive tumor accumulation [69]. To build a translational foundation for actively targeted platforms, several prerequisites must be addressed:

(i) Overcoming physiological barriers for tumor entry: NPs must first extravasate into the tumor tissue. This can be achieved via the reduction of high tumor interstitial fluid pressure (IFP), transient vascular permeabilization, vascular basement membrane degradation, and fibrotic ECM modulation, or by using active transcytosis strategies, utilizing size-tenable platforms, and employing cell/bacteria-based hitchhiking [171–175].
(ii) Optimizing systemic circulation: Targeted NPs require prolonged circulation for optimal tumor accumulation [175]. Approaches like red blood cell (RBC) hitchhiking (RH) have proven effective [176]. For example, RH-liposomes can increase the accumulation of NPs in the first downstream organ (the first organ supplied by the injected blood vessel) subsequent to intravenous (IV) injection. The lung concentration of RH-liposomes reached approximately 40-fold higher levels compared to non-hitchhiked controls [177].
(iii) Advanced understanding of nano-bio interactions and the tumor pathophysiological environment (TPE): Accounting for NP behavior within the biological environment (e.g., protein corona formation) and intricate TPE complexities, including hypoxia, acidity, and enzymatic disturbances, is essential for a strategic targeted NP design [178].
(iv) Meticulous selection of target antigens on cancer cells: Selecting a ligand solely based on high surface expression is insufficient and may not necessarily result in increased cellular uptake. Furthermore, uptake does not guarantee intracellular delivery, as the cargo may undergo endolysosomal degradation. Therefore, along with high surface expression, a cancer cell ligand should (a) internalize after interacting with the targeting moiety and (b) be linked to an intracellular pathway that evades endolysosomal entrapment [179].

Instead of addressing these necessities by optimizing NPs, targeting moieties, ligand-receptor binding affinities, and ligand densities prior to investigation, many studies in the field of lung cancer remain limited to investigating basic cellular uptake and cytotoxicity following active targeting. Preclinical models often fail to capture the complexity and heterogeneity of human tumors, leading to overly optimistic interpretation of therapeutic performance. Improving the physiological relevance of preclinical lung cancer models is therefore critical. While preclinical systems provide controlled platforms for mechanistic and therapeutic evaluation, they incompletely reproduce human disease architecture. Many studies rely on in vitro systems or subcutaneous xenograft models, which fail to capture organ-specific microenvironmental features [180]. Direct comparative study reports approximately 2.5- to 2.8-fold higher NP deposition in subcutaneous tumors compared with orthotopic lung tumors, attributed to increased vascular leakiness and reduced structural fidelity, thereby exaggerating EPR-like effects. Orthotopic models, in contrast, preserve lung-specific vascular architecture and physiological perfusion patterns, more closely approximating human tumor physiology [181]. However, their broader use remains limited due to technical complexity, higher cost, and challenges in defining clinically relevant endpoints; metastatic progression often occurs late, and animals may succumb to primary tumor burden before dissemination becomes measurable [182]. Genetically engineered mouse models offer improved biological relevance but remain constrained by relatively homogeneous genetic backgrounds that do not reflect the mutational burden and intratumoral heterogeneity characteristic of smoking-associated human lung cancers [183].

Beyond model-dependent bias in NP delivery, biological heterogeneity within tumors and the TME represents an additional major constraint on active targeting strategies [184]. Most active targeting studies target a single ligand, which could ultimately result in treatment ineffectiveness; this necessitates harnessing multi-targeting moieties or applying several NPs with different moieties together [179]. On the other hand, the addition of targeting moieties can overcomplicate the fabrication process, heighten regulatory challenges, hamper scalability and industrialization, and increase production costs [185]. Various ligands, such as sialic acid [150], are not cancer-specific, and targeting these ligands can induce off-target effects [186].

Parameters such as PEG-linker length [187, 188] and targeting moiety surface density [189, 190] can affect targeting ability. Fine-tuning the size of NPs and their physicochemical characteristics can also indirectly improve tumor accumulation and subsequently increase active targeting [191]. Strategies such as the tumor-responsive de-shielding of shielded targeting moieties [192], as well as the incorporation of CPPs [193–195], and cancer homing peptides [196], can also attenuate unwanted off-target cellular interactions, extend circulation time, and enhance targeted tumor cell accumulation. Furthermore, advances in ligand bioinformatics and molecular engineering to enhance targeting selectivity, simplify the design principles, and conduct rigorous safety and regulatory studies will pave the way for applied targeted cancer therapy [197].

#### The protein corona: barrier and implication

A major translational bottleneck for active targeting is the formation of the biomolecular corona. Once an NDDS is administered systemically, NPs are exposed to blood components, leading to the rapid formation of a protein corona, which is more broadly considered part of the “biomolecular corona”. This layer transitions dynamically from a loosely bound, high-abundance “soft corona” to an irreversibly bound, high-affinity “hard corona” [198]. This surface layer also defines NPs’ identity and masks surface-modified ligands, thus compromising ligand-receptor interactions [199].

Experimental evidence shows that ligand masking can be prominent. For instance, transferrin-modified NPs exposed to NSCLC patient plasma lose more than 50% of their receptor-mediated uptake, with the active targeting pathway largely abolished [200]. Ligand masking precludes engineered receptor engagement and redirects NP uptake toward alternative biological pathways, including those mediated by scavenger receptors [201]. Combined with the adsorption of opsonins such as Igs and complement proteins, this biological coating promotes rapid macrophage clearance [202]. This phenomenon directly contributes to a persistent clinical translational gap for actively targeted nanomedicines [198].

**Fig. 4:**
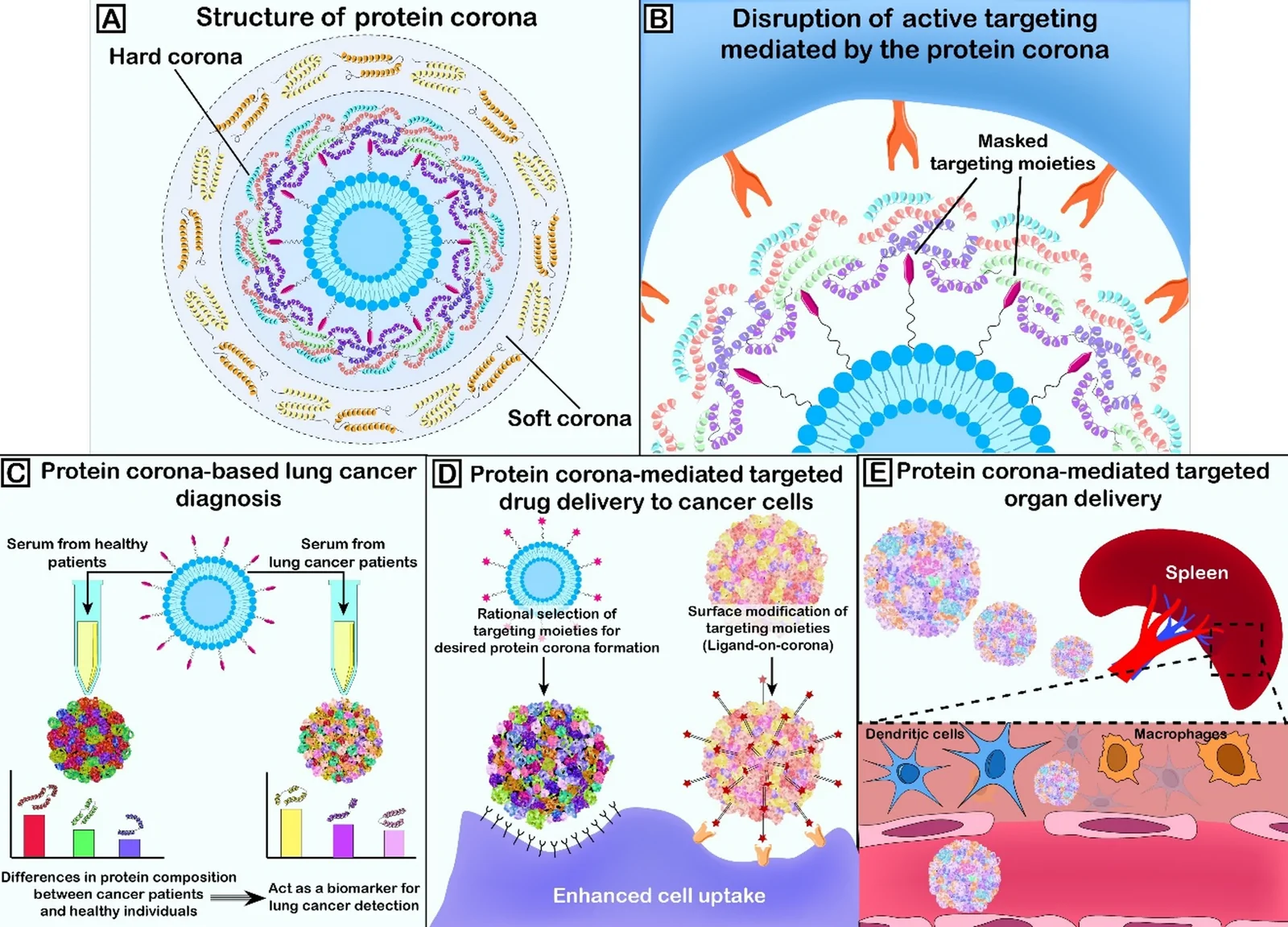
Challenges and opportunities of the protein corona in nanoparticle (NP)-based drug delivery, cancer diagnostics, and targeted therapy. **A** The protein corona forms upon the interaction of serum proteins with NPs, consisting of a loosely bound soft corona and an irreversibly bound hard corona. **B** Corona formation can physically mask surface-engineered targeting moieties, preventing their interaction with cancer cell receptors, thereby impeding active targeting. **C** Analyzing the disease-specific corona formed after exposure of NPs to patient serum enables the differentiation of lung cancer patients from healthy individuals with high sensitivity and specificity. **D** Rational NP design can drive the adsorption of specific endogenous proteins that naturally act as targeting ligands. Additionally, strategies such as attaching targeting moieties to a pre-formed protein corona (the “ligand-on-corona” approach) prevent corona re-formation and preserve targeting capabilities. **E** Exploiting corona-mediated biodistribution enables organ-specific drug delivery. This strategy is particularly effective for delivering immunotherapies and vaccines to secondary lymphoid organs, such as the spleen, which naturally sequester corona-coated NPs

However, the protein corona can also be exploited rather than avoided by recruiting specific endogenous proteins to act as functional interfaces. For example, by harnessing the high affinity of soybean phosphatidylcholine toward apolipoprotein A-I (Apo-AI), an Apo-AI rich protein corona was formed, which facilitated blood-brain barrier (BBB) and glioma targeting [203]. The albumin-binding domain (ABD) was utilized to surface-functionalize liposomes, and resulted in the formation of an albumin-rich protein corona in vivo. In addition to facilitating albumin-mediated cancer cell uptake, this corona effectively prevented the interaction of the nanoplatform with other serum proteins, such as antibodies and complement factors [204]. Optimization of NPs for the formation of organ- or cell-specific protein coronas is another interesting topic. Dextran modification, for instance, resulted in the formation of a specific protein corona that mediated the accumulation of NPs in immune system organs, such as lymph nodes and spleen [205], thereby creating a sophisticated platform for immune activation and vaccine delivery. An additional design concept is the “ligand-on-corona” strategy, in which ligands are conjugated directly to the equilibrated protein corona rather than the bare NP surface, thus preserving receptor accessibility in serum [206]. These results emphasize the rational design of NPs for turning obstacles into stepping stones (Fig. 4).

Furthermore, the formation of the protein corona can be exploited as a diagnostic approach for the detection of lung cancer. Each cancer type exhibits a distinct proteomic profile, resulting in a special protein corona that can be leveraged as a biomarker for cancer detection. In this regard, the IV administration of bare liposomes in in vivo lung cancer (A549) and melanoma (B16-F10) models resulted in the formation of distinct, cancer-specific protein coronas, which consisted of proteins that are hard to detect in serum using conventional methods, such as low-abundance tumor-specific proteins [207]. In another study, the unique biomolecular corona associated with lung cancer allowed a liposomal sensor to discriminate NSCLC patients from healthy individuals with 80% sensitivity and specificity, outperforming routine blood tests [208]. Ren et al. demonstrated that personalized coronas formed on NPs following incubation with the serum of lung cancer patients exhibited idiosyncratic proteomic fingerprints, marked by high levels of complement C1q, which can act as a biomarker for lung cancer detection. Moreover, pre-coating the NPs with C1q activated the classical complement pathway and enhanced macrophage phagocytosis in vitro, thereby providing a potential platform for immunotherapy [209].

However, for the optimal utilization of the protein corona in diagnosis and drug delivery, interpatient variability, such as the presence of comorbid diseases, must not be overlooked. Xu et al. showed that the protein corona composition differs between NSCLC patients and those with comorbid type 2 diabetes; the diabetic comorbidity-specific corona was enriched in clusterin and fibrin. Although both coronas completely abolished transferrin-mediated targeting, the protein corona of comorbid patients was unexpectedly associated with higher cellular uptake and tumor accumulation [210]. This underscores the integral role of tailoring drug delivery systems to the patient-specific proteomic profiles and clinical comorbidities. Furthermore, bridging the clinical translational gap requires treating the biomolecular corona not as a barrier to avoid, but as an inseparable determinant of liposome behavior that dictates applying rational design strategies to harness its potential.

### Overcoming multidrug resistance

In cancer therapy, multidrug resistance (MDR) often arises from the overexpression of transmembrane drug transporters that efflux chemotherapeutic agents out of cancer cells [211]. In lung cancer, this includes ATP-binding cassette (ABC) transporters such as P-glycoprotein (P-gp), breast cancer resistance protein (BCRP), and multidrug resistance protein (MRP), as well as altered expression of solute carrier (SLC) transporters—for example, glucose transporter GLUT1 overactivation, which drives resistance to EGFR TKIs [212]. Elevated expression of MRP1, lung resistance-related protein (LRP), and the excision repair cross-complementing 1 (ERCC1) is associated with Cis-based chemotherapy failure and poor patient prognosis [213].

While transporter overexpression represents the canonical mechanism of MDR, its activity is driven by upstream alterations in epigenetic regulation, which promote transcription of the gene encoding P-gp [214], and by mitochondrial metabolism, which sustains ATP-dependent efflux [215]. MDR can also be further reinforced by dysregulation of apoptotic signaling, which raises the threshold for cell death and enhances survival under chemotherapeutic stress [214]. As reviewed by Li et al., despite decades of research, no therapy targeting these transporters has reached clinical translation due to insufficient potency, off-target toxicity, and lack of predictive biomarkers to identify which patients are likely to respond [212]. To circumvent MDR in lung cancer, researchers have designed liposomal systems that can be categorized into distinct molecular strategies, progressing logically from single-target interventions to highly integrated platforms.

#### Transporter-mediated resistance (efflux pump inhibition)

The most direct approach to reversing acquired MDR involves the physical or competitive inhibition of ABC transporters at the plasma membrane. Some early liposomal designs leveraged repurposed pharmacological agents to achieve broad-spectrum transporter inhibition. For instance, Ju et al. (2017) utilized CPP (PTD)-modified liposomes to co-deliver tamoxifen, a first-generation ABC transporter inhibitor, and paclitaxel (PTX), reporting successful downregulation of ABCB1 (P-gp), ABCC1 (MRP1), and ABCG2 (BCRP) protein expression in resistant lung cancer cells. However, this study was limited to phenotypic outcomes, as it did not elucidate whether this protein suppression occurred at the transcriptional, post-transcriptional, or post-translational level [216].

In another study, researchers utilized DOX-loaded liposomes coated with d-α-tocopheryl polyethylene glycol succinate (TPGS), a surfactant polymer with P-gp inhibitory activity [217]. While the exact kinetics of these pathways were not quantified, this specific design highlights an attempt to exploit distinct biophysical interactions rather than relying on pharmacological blockade. The authors propose several rational mechanisms for MDR reversal in this system. First, through substrate masking, the TPGS coating on liposomes hides the drug so that P-gp does not recognize it as a substrate, thereby avoiding efflux. Additionally, the vitamin E moiety of TPGS on the surfaces of the liposomes may act as a ligand that interacts with receptors on the cell membrane, inducing receptor-mediated endocytosis and promoting the intracellular uptake of drugs.

#### Upstream resistance modulation: organelle and epigenetic targeting

Another strategy uses liposomes designed to bypass or neutralize the cell’s survival signaling entirely. These platforms aim to either directly trigger the intrinsic apoptotic cascade within the organelle space or modulate upstream resistance-regulating pathways.

Jiang et al. exemplified direct organelle targeting: they engineered a pH-responsive liposome functionalized with the pro-apoptotic peptide KLA. Following endosomal escape, facilitated by the peptide’s lysine residues, KLA specifically targets the mitochondrial membrane, inducing a great loss of mitochondrial membrane potential and subsequent cytochrome c release. This forced apoptosis in 65.1% of Taxol‑resistant A549 (A549/Taxol) cells reduced the IC_50_ of PTX by 20-fold compared to free drug, and achieved 86.7% tumor growth inhibition in vivo [218].

A different approach focuses on the upstream regulatory networks that cause resistance. To overcome Cis resistance in lung cancer, Li and colleagues engineered pH-responsive liposomes hybridized with Cis-resistant A549/Cis cancer cell membranes. This biomimetic coating enabled homotypic targeting and enhanced intratumoral accumulation of Cis, thereby mitigating drug resistance (the biomimetic coating strategy is further discussed in the section on biomimetic and hybrid liposomal carriers). In parallel, the NDDS delivered EIP103, a nucleus-targeting antagonist peptide that binds to the epigenetic regulator Zeste homolog 2 (EZH2), which is overexpressed in Cis-resistant NSCLC. The system modulates EZH2-associated transcriptional repression networks, thereby resensitizing tumor cells to Cis and activating caspase-dependent apoptosis [219].

#### Dual and multi-mechanistic targeting platforms

Moving toward more complex architectures, other studies employ liposomes to co-deliver agents that reverse MDR through multiple synergistic, non-competitive mechanisms. Because ABC transporters are strictly ATP-dependent [220], these platforms frequently combine direct transporter inhibition with mitochondrial disruption to deplete cellular ATP and impair efflux activity. For example, Li et al. administered TPGS-modified lonidamine (LND) liposomes alongside TPGS-modified epirubicin liposomes. LND, a mitochondrial disruptor, depletes intracellular ATP—the essential fuel for P-gp efflux pumps. The LND-containing liposomes were further functionalized with dequalinium (DQA), a mitochondria-targeting moiety. In this dual-liposome platform, TPGS directly inhibits ABC transporter-mediated drug efflux, while LND reduces ATP availability, indirectly starving efflux pumps, and triggers mitochondrial apoptosis [221]. In another study, researchers delivered a mitochondrial-targeted liposomal system (TT LP/PTX) incorporating TPGS and TT, an amphipathic polymer conjugate of triphenylphosphine (TPP), to overcome P-gp-mediated drug resistance in NSCLC cells. TT facilitated mitochondrial delivery, while TPGS inhibited P-gp ATPase activity. Mitochondrial accumulation increased reactive oxygen species (ROS), reduced ATP levels, and dissipated mitochondrial membrane potential, ultimately impairing P-gp function and enhancing PTX cytotoxicity in resistant A549/Taxol cells [222].

As the field evolves, it is increasingly moving toward multi-component liposomal platforms that simultaneously address membrane efflux, organelle dysfunction, and microenvironmental resistance. A representative example is the Pluronic P105-hybridized PTX liposome (PPL) system combined with ambroxol. In this design, the liposome serves multiple roles: it delivers PTX, incorporates Pluronic P105 into the lipid bilayer to downregulate P-gp expression, and utilizes a dipalmitoylphosphatidylcholine (DPPC) scaffold that interacts with surfactant protein A (SP-A) for enhanced pulmonary accumulation. Co-administered ambroxol further upregulates SP-A in the lung TME and inhibits cytoprotective autophagy, collectively sensitizing multidrug-resistant NSCLC cells. Such integrated approaches illustrate the potential of rationally designed liposomes to tackle MDR through coordinated multi-pathway modulation [223].

## Liposomal drug delivery for lung cancer therapy: barrier-centric applications

### Chemotherapeutics

The bilayer architecture of liposomes provides a distinct advantage for delivering diverse therapeutic molecules to various tumors, including lung cancer [224]. This versatility accommodates hydrophobic agents, such as paclitaxel (PTX), and hydrophilic drugs, including cisplatin (Cis), doxorubicin (DOX), or 5-fluorouracil [225–227], and allows co-encapsulation of compounds with differing solubility. Recent studies have leveraged liposomes to co-deliver chemotherapeutics with complementary cytotoxic or adjuvant agents. Ghosh et al. co-loaded vincristine and DOX into the aqueous core via an ammonium-ion gradient, enhancing antitumor efficacy without increasing systemic toxicity; cholesterol maintained vesicle integrity and minimized leakage [228]. Similarly, Wang et al. designed a liposomal platform for NSCLC that co-delivers daunorubicin, a hydrophilic cytotoxic actively loaded into the aqueous core, and dioscin, a hydrophobic antimetastatic agent passively incorporated into the bilayer. Surface modification with the neutral hydrophobic CPP, PFVYLI, significantly improved cellular uptake in A549 cells without inducing systemic toxicity, successfully triggering ROS-mediated apoptosis and suppressing invasion, migration, and vasculogenic mimicry [229]. Formulation parameters, such as vesicle size, also influence off-target toxicity and tissue distribution. In PTX liposomes, multilamellar vesicles (~ 200 nm) prevented peripheral neuropathy, whereas smaller unilamellar vesicles (~ 100 nm) caused toxicity comparable to free drug. This reflects size-dependent penetration through dorsal root ganglia capillaries (80–100 nm), highlighting how spatial restriction of liposomal distribution can protect sensory neurons while maintaining antitumor efficacy [230].

Liposomal formulations of several chemotherapeutic agents used in SCLC and NSCLC have advanced to clinical trials. However, according to FDA approval records and the available published literature, no liposomal chemotherapy has been approved by the FDA for lung cancer. Lipusu (liposomal PTX, approved in China for NSCLC) and Onivyde (liposomal irinotecan, evaluated in Phase III for relapsed SCLC) represent the closest attempts at regulatory approval of liposomal chemotherapies for lung cancer. Several factors account for this translational gap. Although liposomal irinotecan (IRI; ONIVYDE^®^) is FDA-approved for patients with metastatic pancreatic adenocarcinoma [231], it has not achieved approval for SCLC. In the phase III RESILIENT trial (NCT03088813), liposomal IRI was compared with topotecan in relapsed SCLC. The primary endpoint of overall survival (OS) was not met (median OS 7.9 vs. 8.3 months; Hazard ratio (HR) 1.11, *P* = 0.31), despite a higher objective response rate (ORR) (44.1% vs. 21.6%) [232]. This dissociation between response and survival may reflect the characteristic biology of SCLC, which is marked by high initial chemosensitivity followed by rapid emergence of resistant clones [233]. One possible explanation is that IRI induced cytoreduction reflected in improved ORR, while resistant subclones with alternative DNA damage response pathways rapidly emerged [234], limiting durable disease control. In oncology practice, OS remains the gold‑standard endpoint for regulatory approval decisions in SCLC [235], the absence of an OS benefit limits its regulatory impact.

A similar pattern is observed with Lipusu^®^, a PTX liposome injection, which has been clinically evaluated in NSCLC and other solid tumors [236]. In a large multicenter randomized trial (NCT02996214), Lipusu plus Cis showed comparable efficacy to gemcitabine (GEM) plus Cis as first-line therapy in advanced squamous NSCLC, with nearly identical progression-free survival (PFS) (5.2 vs. 5.5 months), OS (14.6 vs. 12.5 months), ORR (41.8% vs. 45.9%), and disease control rate (90.3% vs. 88.1%) [237]. Although Lipusu offered better tolerability, reflected by fewer treatment interruptions and discontinuations, it failed to demonstrate superior antitumor efficacy. Lipusu is not FDA-approved for lung cancer in the United States. This exemplifies a recurring translational limitation of liposomal chemotherapies: meaningful improvements in safety and pharmacokinetics are often achieved, yet they rarely translate into clinically significant survival gains [238]. Without clear superiority in efficacy over optimized standard-of-care regimens, the added manufacturing complexity and higher cost of liposomal formulations become difficult to justify for regulatory approval and widespread adoption. Additionally, the study was powered to show superiority of Lipusu plus Cis over GEM plus Cis, rather than against the more relevant solvent-based PTX plus platinum comparator. Consequently, the trial design does not allow attribution of any observed effects to the liposomal formulation itself. A detailed summary of the relevant clinical trials involving liposomal chemotherapies for lung cancer is provided in Table 2.

**Table 2:** Summary of recent clinical trials of liposomal chemotherapies in lung cancer

Condition	Treatment	Study Phase	Country	ID # (clinicaltrials.gov)	Results
SCLC	TPT Liposomes	Phase 1	US	NCT00765973	No published results
SCLC	IRI Liposome	phase 2	China	NCT04727853	No published results
Relapsed SCLC	IRI Hydrochloride Liposome (LY01610)	Phase 2	China	NCT04381910	ORR 32%, DCR 68%; median PFS 4.0 mo, median OS 9.7 mo; 1-year OS 40%; manageable hematologic and GI toxicity [239].
SCLC	PEGylated Liposomal IRI (ONIVYDE^®^) vs. TPT	Phase 3	Multinational	NCT03088813	Primary OS endpoint not met (7.9 vs. 8.3 mo). Similar PFS (4.0 vs. 3.3 mo). Higher ORR with liposomal IRI (44.1% vs. 21.6%). Lower grade ≥ 3 TEAEs vs. TPT [232].
NSCLC, stage 3b-4	Aerosolized Liposomal Camptothecin	Observational	US	NCT00277082	No published results
Recurrent SCLC	Liposomal Lurtotecan (OSI-211)	Phase 2	Multinational	NCT00046787	No published results
NSCLC	Liposomal DTX (ATI-1123)	Phase 1	US	NCT01041235	One NSCLC patient achieved partial response (PR) with time to disease progression of 30 weeks; other NSCLC patients had SD with tumor reductions of 11-19.6% [240].
NSCLC	GEM Liposome (FF-10832) ± Pembrolizumab	Phase 2	US	NCT05318573	Six NSCLC patients treated with a median of 2 cycles. Treatment generally well tolerated. No confirmed responses; some patients achieved SD. Median PFS 6 weeks, median OS 23.3 weeks. Expansion Phase ongoing [241].
SCLC	Liposomal Eribulin (E7389-LF) + Nivolumab	Phase 1b/2	Japan	NCT04078295	Thirty-three evaluable SCLC patients received E7389-LF plus nivolumab. The combination achieved an ORR of 24.2% and a DCR of 75.8%. Median PFS was 3.98 months, and the 6-month OS rate was 90.9%. Treatment-related TEAEs occurred in 97.1% of patients, with grade ≥ 3 events in 82.4%, most commonly neutropenia. No new safety signals were identified [242].
Squamous NSCLC, stage 3b-4	Cis+ Lipusu (PTX liposome) vs. Cis + GEM	Phase 3	China	NCT02996214	In per-protocol set, median PFS was 5.2 vs. 5.5 mo (Lipusu vs. GEM), median OS 14.6 vs. 12.5 mo; ORR 41.8% vs. 45.9%, DCR 90.3% vs. 88.1%. The Lipusu combination demonstrated a more favorable safety profile, with fewer patients requiring treatment interruptions or discontinuation [237].

*SCLC* Small cell lung cancer, *TPT* Topotecan, *IRI* Irinotecan, *ORR* Objective response rate, *DCR* Disease control rate, *PFS* Progression-free survival, *mo* months, *OS* Overall survival, *GI* Gastrointestinal, *TEAEs* Treatment-emergent adverse events, *NSCLC* Non-small cell lung cancer, *DTX* Docetaxel, *PR* Partial response, *Cis* Cisplatin, *GEM* Gemcitabine, *PTX* Paclitaxel, *SD* Stable disease.

### Immunotherapy and cancer vaccines

Survival outcomes in lung cancer, which have been historically abysmal, have continually prompted scientists to search for approaches beyond the long-used surgery-chemotherapy-radiotherapy combination [243]. Cancer immunotherapy has emerged as an increasingly recognized therapeutic pillar in combating malignancies. It works by reinforcing the anticancer immune response, mitigating the immunosuppressive TME, and initiating prophylactic and therapeutic immune memory, thus restraining tumor growth and progression [244]. Generating a potent antitumor immune response is particularly pursued by scientists in lung cancer treatment. The NSCLC TME is profoundly immunosuppressive, characterized by tumor-associated immune cells and immune-inhibitory cytokines that suppress effective immunity. Tumor-infiltrating lymphocytes also face difficulty penetrating the TME due to abnormal vasculature and stromal barriers. Together, these features restrict the efficacy of non-targeted immunotherapeutics, which often fail to sufficiently engage or activate antitumor immunity [245]. Immunotherapy comprises several main arrays, including immune checkpoint blockade (ICB) [246], cancer vaccines [247], and immune-cell reprogramming strategies [248]. Among these, cancer vaccines have frequently relied on nanocarrier systems, particularly lipid nanoparticles (LNPs). These delivery platforms offer structural and functional advantages by protecting antigen and adjuvant components from degradation, enhancing uptake by antigen-presenting cells (APCs) and lymphoid tissues, and enabling co-delivery and controlled release [249, 250]. Lung cancer has been under the spotlight in cancer immunotherapy, and a growing body of clinical and preclinical studies on the liposomal delivery of immunotherapeutics has been conducted to evaluate and potentially enhance these approaches in combating this disease, as detailed in the following sections.

#### Overcoming poor immunogenicity: liposomal cancer vaccines

Cancer vaccines hold great potential in cancer immunotherapy, with liposomes being a widely leveraged carrier [251]. In principle, cancer vaccines consist primarily of antigens that trigger an immune response and adjuvants that enhance nonspecific immunity. Antigens are categorized into tumor-associated antigens (TAAs) and tumor-specific antigens (TSAs) and may be delivered as peptides, proteins, or nucleic acids encoding these antigens, such as DNA or mRNA [252]. Conventional tumor vaccines, however, face several shortcomings. Free peptides, proteins, and nucleic acids are susceptible to enzymatic degradation, and the lack of a protective carrier results in a short in vivo half-life. In addition, without protective nanocarriers, soluble antigens and conventional adjuvants are poorly internalized by DCs and exhibit limited lymph node drainage [253]. Furthermore, the therapeutic benefit of co-delivering antigen and adjuvant to the same APC has motivated scientists to explore liposomes as efficient and versatile nanocarriers for cancer vaccination. Liposomes, as amphiphilic vesicles with a hydrophilic core and lipid bilayer, can encapsulate both water- and lipid-soluble cargos, protect them from degradation, enable targeted delivery, and even act as adjuvants themselves [254].

An important limitation in cancer vaccination is the ineffective delivery of antigenic peptides and immune adjuvants to the same APC, which compromises coordinated T-cell activation [255]. Jacoberger-Foissac et al. engineered dual-epitope liposomes displaying the E7C CD8 tumor epitope and the hemagglutinin A CD4 helper epitope, together with a pattern recognition receptor (PRR) agonist. This platform enabled coordinated antigen presentation and T-cell priming. Using the TC-1 disseminated tumor model, the authors showed that the lower adjuvant dose (2 µg) reduced pulmonary nodules more effectively than a ten-fold higher dose, which the authors attributed to excessive inflammation at the higher dose. Thus, the liposomal platform addressed the co-delivery barrier, but adjuvant dose optimization remains critical [256].

Despite their clinical progress [257], RNA vaccines face major delivery barriers, which researchers have aimed to overcome using liposomal formulations. Because mRNAs are highly negatively charged, cationic liposomes have been widely explored for their complexation and delivery [258]. Additionally, cationic lipids such as 1,2-dioleoyl-3-trimethylammonium-propane (DOTAP) can exert intrinsic immunostimulatory effects, promoting DC activation and T-cell responses, which may further enhance vaccine efficacy [259]. However, particle surface charge strongly influences in vivo behavior, and ionizable or even anionic formulations are increasingly used to improve biodistribution and safety [260]. Efficient loading of mRNA into liposomes can be challenging, and various lipid formulations have been explored to improve encapsulation and delivery efficiency [261]. To overcome this limitation, Ma et al. pre-condensed the mRNA with protamine, a small cationic protein, forming a compact mRNA-protamine complex that enabled efficient liposomal encapsulation. They selected melanoma-associated antigen A1 (MAGE-A1) mRNA because it encodes a highly immunogenic antigen overexpressed in multiple malignancies and implicated in tumor progression, making it a suitable model antigen for evaluating antitumor vaccine efficacy. Their liposomal vaccine elicited MAGE-A1-specific CD8⁺ cytotoxic and CD4⁺ helper T-cell responses and suppressed primary lung tumor growth (from ~ 1807 mm³ in controls to ~ 781 mm³; *p* < 0.001) and bone metastasis in mouse models, indicating its potential as a liposomal mRNA delivery strategy for lung cancer [262].

These advantages have not, however, translated consistently into clinical benefit, as several structural features of late-stage lung cancer trials and the immunobiology of the TME have systematically undermined vaccine efficacy. In contrast to preclinical systems, where immune responses and survival outcomes can be evaluated over short time frames, clinical trials often require longer follow-up to detect treatment effects typical of immunotherapy, such as delayed survival benefits and late separation of survival curves [263]. While immunogenicity can be detected early, OS remains dependent on the extended disease course, prior lines of therapy, and multiple confounding clinical variables [264–266]. Trial design further contributes to limited efficacy signals, as vaccine strategies in lung cancer have often been evaluated in late-stage or heavily pretreated populations [267], which are frequently characterized by an exhausted immune system and tumor-driven immunosuppression, conditions under which vaccine-induced responses are unlikely to translate into durable tumor control [266, 268].

A prominent example is the liposomal MUC1 vaccine L-BLP25 (tecemotide), which incorporates a synthetic MUC1-derived lipopeptide, a glycoprotein overexpressed in NSCLC, together with monophosphoryl lipid A as an adjuvant [269]. Across multiple clinical studies, L-BLP25 demonstrated an acceptable safety profile but did not yield a statistically significant improvement in OS within the overall treated populations [270, 271]. In an updated survival analysis of an earlier Phase IIb trial (NCT00157209), L-BLP25 demonstrated a numerical improvement in median OS compared with best supportive care (17.2 vs. 13.0 months), although this did not reach statistical significance in the intent-to-treat population [272]. In the phase III START trial (NCT00409188), conducted in patients with unresectable stage III NSCLC, the vaccine again failed to improve OS in the overall cohort, with benefit restricted to the subgroup receiving concurrent chemoradiotherapy [273]. A subsequent randomized study in a Japanese population did not reproduce this subgroup signal, including in patients receiving concurrent chemoradiotherapy, confirming a lack of reproducible survival advantage [271]. The initial START failure, together with negative results from the Japanese study, led the sponsor to discontinue the entire tecemotide clinical development program, resulting in the premature termination of the confirmatory START2 (NCT02049151) and INSPIRE (NCT01015443) Phase III trials [274–276].

Two converging factors might explain these results. Patient selection was not guided by biomarkers such as MUC1 expression level or baseline immune status, resulting in substantial population heterogeneity. Furthermore, MUC1 is a shared self-antigen subject to thymic tolerance [277], with clinical studies of MUC1-based vaccines showing inconsistent T-cell responses [278]. This may be particularly consequential in lung cancer subtypes, where high tumor mutational burden generates a rich neoantigen landscape [279], that single-target vaccines are unequipped to exploit [278, 279].

The clinical trial design and therapeutic regimen further influenced outcomes. In major trials, L-BLP25 was administered after or in combination with chemoradiotherapy and positioned as a maintenance therapy. Although concurrent chemoradiotherapy can transiently enhance antigen release and induce lymphopenia that may favor T-cell priming [280], it can also cause prolonged immunosuppression [281]. By the time vaccination was initiated, patients had often received intensive treatment, potentially limiting immune responsiveness. The experience with L-BLP25 and other early cancer vaccines has prompted a reassessment of cancer vaccine design and immunotherapy regimens. Increasingly, the field has favored multicomponent immunomodulatory systems incorporating antigen sources, potent adjuvants, and immune-modulating co-therapies [282].

Current efforts frequently focus on multi-antigen and personalized neoantigen mRNA vaccines delivered via lipid NPs, reflecting a shift from earlier liposomal and peptide-based vaccine platforms [283]. Furthermore, clinical studies across solid tumors routinely evaluate cancer vaccines in combination with ICBs [284–286]. This transition reflects the recognition of two central constraints in lung cancer immunotherapy: pronounced intratumoral heterogeneity and an immunosuppressive TME [287, 288]. While ICB addresses inhibitory signaling within the tumor milieu [289], next-generation vaccine strategies aim to incorporate antigenic diversity and patient-specific tumor profiles [290], to improve the likelihood of durable clinical responses. Despite the mixed clinical outcomes, the versatility of liposomes confers substantial future potential. Liposomal cancer vaccines represent a promising avenue for lung cancer immunotherapy by addressing key limitations of conventional vaccine platforms. In this context, rational design is critical; optimized physicochemical properties, particularly particle sizes in the ~ 10–100 nm range, facilitate efficient lymphatic drainage, LN retention, and APC capture, thereby enabling robust antitumor immune responses in preclinical cancer vaccine models [291]. Looking ahead, through multiepitopic antigen presentation, co-encapsulation of antigens and adjuvants, and optimization of lipid composition to incorporate intrinsic immunostimulatory effects, liposomes may elicit more pronounced antitumor immunity and pave the way for more efficacious, tailored lung cancer immunotherapies.

#### Overcoming poor immunogenicity: liposomal immune adjuvants

Poor tumor immunogenicity drives the induction of immune tolerance, T‑cell anergy, and the selection of immune‑resistant tumor variants [292]. Therefore, immune adjuvant has emerged as another main immunotherapeutic strategy in cancer treatment. However, the main limitation to the effective use of free adjuvants such as CpG oligodeoxynucleotides (CpG ODNs) is that their high potency is counterbalanced by rapid systemic diffusion, poor bioavailability, and off‑target toxicity, which necessitates the use of delivery systems to localize immune activation and reduce side effects [293].

Another immunotherapeutic strategy in lung cancer, as in other solid tumors, is to deliver immune-sensing molecules, such as Toll-like receptor (TLR) agonists and stimulator of interferon genes (STING) agonists, to elicit antitumor immunity [294]. To deliver TLR agonists, which are predominantly negatively charged, Loira-Pastoriza et al. engineered cationic liposomes by incorporating DOTAP and DPPC. While the larger and more complex TLR agonist polycytidylic acid [poly(I: C)] could not be encapsulated efficiently, the researchers successfully delivered the CpG oligonucleotide via inhalation aerosols. Encapsulation of CpG within biocompatible, positively charged liposomes enhanced its stability and targeted delivery to lung immune cells, resulting in over a twofold reduction in tumor cells and improved antitumor efficacy in a mouse model of lung metastasis, outperforming non-encapsulated CpG [295].

STING agonists are another class of immune-sensing immunotherapeutics for lung cancer. By activating the cGAS-STING pathway, they induce type I interferons and proinflammatory cytokines, thereby triggering both innate and adaptive immune responses to promote an antitumor effect [296]. Studies have demonstrated that liposomes are efficient nanocarriers for delivering immunostimulatory molecules, either as monotherapies or in combination with other immunotherapeutic agents, particularly ICBs. Zhu et al. utilized liposomes as nanocarriers to deliver the STING agonist 2′3′-Cyclic GMP-AMP (2′,3′-cGAMP), which was encapsulated in the aqueous core of the liposome. Anti-PDL1 antibodies and CXCR3 antagonists (CXCR3 is a chemokine receptor that regulates immune cell trafficking in the TME) were also co-administered as part of the treatment regimen in a murine lung cancer model. Liposomal delivery ensured cGAMP stability, promoted activation of the cGAS-STING pathway, and modulated the TME, resulting in reduced tumor growth, improved immune cell infiltration, and minimized systemic toxicity [297]. Another study led by Chen et al. demonstrated that liposomes can also be customized to potentiate the delivery of MSA-2, a potent non-nucleotide STING agonist with previously unfavorable bioavailability. By esterifying the carboxyl group of MSA-2, the authors generated esterase-responsive prodrug derivatives that could be efficiently incorporated into the liposomal bilayer. This innovative strategy markedly improved the pharmacokinetic profile of MSA-2, boosted antitumor immunity, achieved complete tumor regression, suppressed lung metastases, prolonged survival, and minimized systemic toxicity in preclinical lung cancer models. This study underscored the necessity of drug-liposome co-engineering to advance STING-based immunotherapies for lung cancer [298].

However, despite encouraging preclinical outcomes, translating liposome-based and other NDDSs for immune adjuvant delivery requires further testing because their biodistribution and interactions with biological barriers, such as clearance by the RES, remain challenging to predict. Additionally, delivering multiple adjuvants alongside vaccine components necessitates careful optimization of formulation parameters and dosing ratios to achieve synergistic antitumor effects while minimizing unwanted toxicity [299]. Furthermore, the systemic diffusion of highly potent free adjuvants (such as TLR7/8 or TLR9 agonists) is known to trigger severe off-target toxicity and systemic cytokine release syndromes [300]. Moreover, premature drug leakage from liposomes during systemic circulation is a recognized limitation of conventional liposomal formulations, as even minor uncontrolled release can lead to off-target toxicity and adverse effects [301].

#### Overcoming heterogeneity and resistance in immune checkpoint blockade

Immune checkpoint blockade (ICB) represents one of the most established and transformative pillars of cancer immunotherapy, particularly for advanced NSCLC and extensive-stage SCLC. ICB therapies, such as pembrolizumab and atezolizumab, have significantly improved OS rates [302]. A systematic review and meta-analysis of 8 studies involving 988 NSCLC patients found that ICBs improved OS in patients with brain metastases (when combined with chemotherapy) and liver metastases (when used alone or with chemotherapy plus anti-vascular endothelial growth factor (VEGF) therapy) [303]. Furthermore, combining chemotherapy with ICBs, as demonstrated in landmark trials like KEYNOTE-189 and RATIONALE 307, further enhances treatment efficacy [304, 305]. However, limitations of ICB therapy persist, including primary and acquired resistance driven by tumor-intrinsic factors (e.g., gene mutations, lack of antigen presentation) and tumor-extrinsic factors (e.g., immunosuppressive TME) [306]. Although the development of immune-related adverse events (irAEs) associated with ICB therapy is often considered a favorable prognostic sign, these events can negatively affect patients’ quality of life and frequently necessitate the discontinuation of therapy [307].

Scientists have primarily used liposomal platforms to deliver immunomodulatory agents, which are often co-administered with systemic ICBs, thus enhancing the therapeutic efficacy of programmed cell death protein 1 (PD1)/programmed death-ligand 1 (PDL1)-based therapies. For example, in a recent retrospective study of 49 patients with advanced NSCLC, the combination of PTX liposome-based chemotherapy and PD1/PDL1 inhibitors, such as camrelizumab and tislelizumab, demonstrated improved outcomes. The median PFS was 9.7 months, and the median OS reached 30.5 months, comparing favorably to historical chemotherapy-alone benchmarks [308].

To address these persisting limitations of conventional ICB therapy, liposomal delivery has emerged as a promising approach to improve the efficacy and safety of ICB therapy through several mechanisms [309]. One rational design involves surface coating of liposomes with ICB antibodies [310]. Studies discussed below demonstrate that liposomal delivery enables multifunctional cancer therapy by presenting the ICB agent on the liposome surface while simultaneously encapsulating other therapeutic agents within the core or the lipid bilayer. For example, Yin et al. developed dual-targeting liposomes by incorporating an anti-PDL1 nanobody (a single-domain antibody fragment that is smaller in size and often exhibits higher affinity than conventional antibodies [311]) and a TfR-binding peptide (T12). These functionalized liposomes, co-loaded with simvastatin (SIM) and gefitinib, efficiently crossed the BBB and specifically accumulated in brain metastases of lung cancer. Rational design of liposomes, such as functionalization with anti-PDL1 nanobodies, enabled the targeted delivery of the TAM reprogramming agent, SIM, to TAMs, thus repolarizing immunosuppressive M2 macrophages to the antitumor M1 phenotype and potentiating the immunomodulatory effects of the therapy. However, while the dual-targeted liposomal formulation prolonged survival compared to free gefitinib and simvastatin (SIM) in combination (19 vs. 29 days), its median survival remained inferior to the positive control, osimertinib (OSI) (29 vs. 33 days) [312]. This study represents a broader translational challenge for nanomedicine: increasingly complex multifunctional nanoplatforms must demonstrate clear therapeutic advantages over established, clinical-standard targeted therapies to justify the profound manufacturing complexity, regulatory burden, and development costs associated with their translation.

**Fig. 5:**
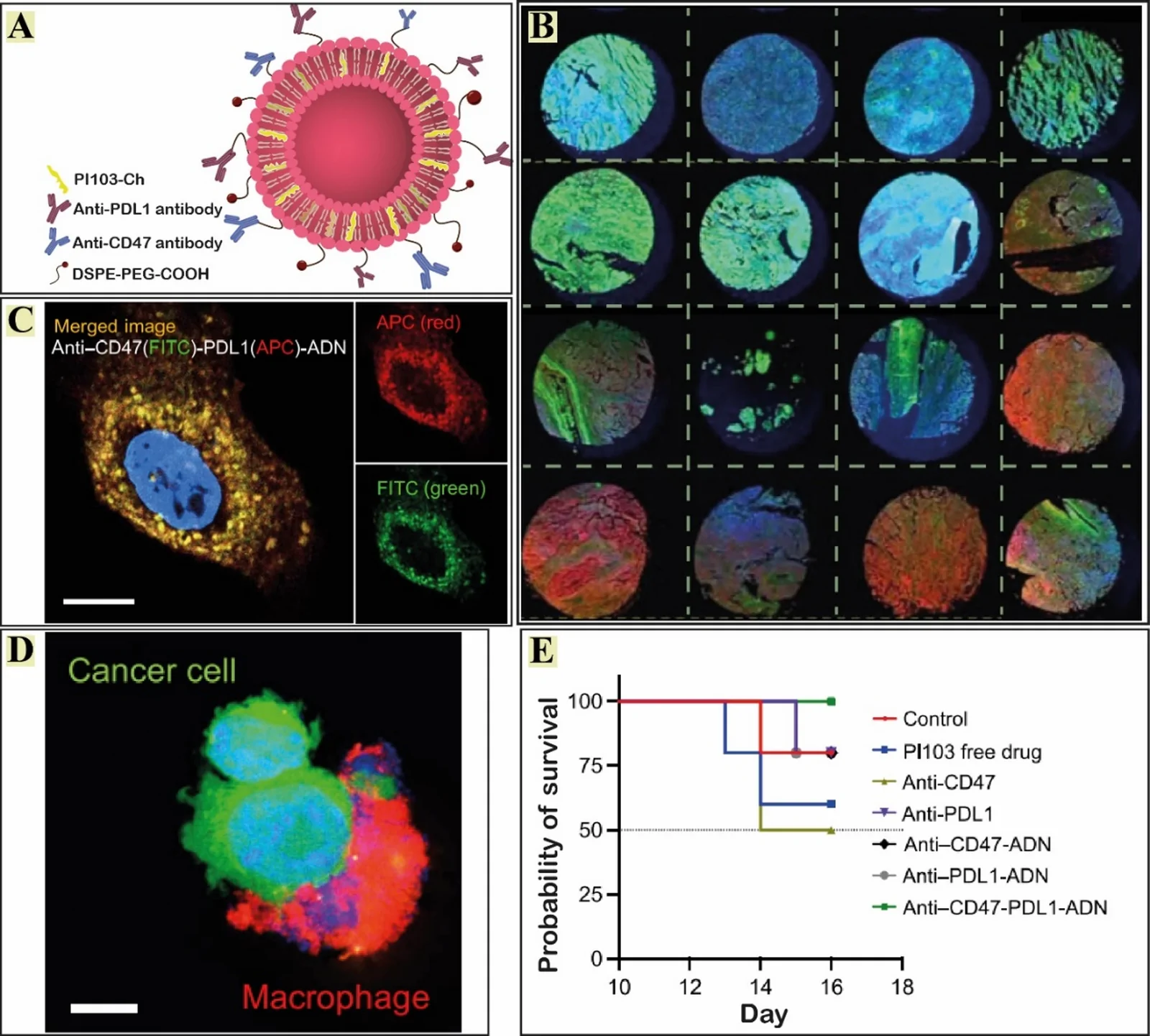
**A** Schematic depiction of a liposomal NP loaded with PI103-Ch, a dual PI3K/mTOR inhibitor, within the lipid bilayer and surface-decorated with both anti-CD47 and anti-PDL1 monoclonal antibodies, referred to as anti-CD47-PDL1-ADN. **B** Immunofluorescence staining of human NSCLC tissue microarray sections using anti-CD47 (green), anti-PDL1 (red), and DAPI nuclear counterstain (blue). Individual cores from different patients illustrate inter-patient variability, with some tumors predominantly expressing CD47 and others primarily expressing PDL1. **C** Confocal microscopy image demonstrating uptake of dual-labeled anti-CD47(FITC)-PD-L1(allophycocyanin [APC])-ADN by A549 human lung adenocarcinoma cells (scale bar, 10 μm). Yellow colocalization confirms the presence of both antibodies on the same NPs. **D** Confocal image showing enhanced phagocytosis of LLC cells by RAW 264.7 macrophages when treated with anti-CD47-PDL1-ADN (scale bar, 10 μm). **E**) Kaplan-Meier survival analysis for tumor-bearing mice in different treatment groups, showing complete (100%) survival at day 15 in the anti-CD47-PDL1-ADN group, in contrast to lower survival rates observed with monospecific ADNs or control treatments. Abbreviations: ADN, antibody-conjugated drug-loaded nanotherapeutics; PI3K, phosphatidylinositol 3-kinase; mTOR, mammalian target of rapamycin. Adapted and rearranged with permission from ref [314]. Copyright © 2024 the Authors. Distributed under the Creative Commons Attribution-NonCommercial 4.0 International License (CC BY-NC 4.0)

Immune checkpoint expression is highly heterogeneous among patients, posing a great barrier to the therapeutic response to ICB therapy [313]. To support this concept, Saha et al. analyzed over 80 patient specimens, confirming that very few tumors expressed both checkpoints at high levels, meaning that most expressed either CD47 or PDL1. In response, they engineered a multifunctional liposomal platform, referred to as antibody-conjugated drug-loaded nanotherapeutics (ADN), which were co-conjugated with anti-CD47 and anti-PDL1 antibodies to simultaneously block innate (CD47-mediated “don’t eat me” signals to macrophages) and adaptive (PDL1–PD1-mediated T-cell suppression) immune checkpoints on NSCLC cells, while delivering PI103-Ch, a cholesterol-conjugated dual phosphatidylinositol 3-kinase (PI3K)/mammalian target of rapamycin (mTOR) inhibitor, directly into tumors (Fig. 5A, B, and C). The dual anti-CD47-PDL1 ADN showed superior in vitro cytotoxicity, phagocytosis induction, and pathway inhibition, with reduced toxicity compared to other treatment groups (Fig. 5D). In vivo, it significantly inhibited tumor growth and achieved 100% survival in two complementary syngeneic models reflecting real-world differential immune checkpoint expression (Lewis lung carcinoma (LLC) with high CD47 and low PDL1 expression; KLN-205 with low CD47 and high PDL1 expression), a finding that may help address clinically relevant intertumoral heterogeneity and highlight the potential of this multimodal strategy for NSCLC (Fig. 5E) [314].

### Phytotherapy

#### Phytochemical-loaded liposomes: overcoming bioavailability and delivery barriers

Phytochemicals (PhCs) have received considerable attention in cancer research due to their pleiotropic nature, which modulates diverse cellular pathways while grappling with a multitude of oncogenic mechanisms to confront cancer [315–319]. Suboptimal efficacy of conventional oncotherapy has encouraged researchers to explore alternative treatment lines, such as PhCs, to act as therapeutic or palliative options. However, the clinical application of PhCs is hampered by their poor intestinal absorption, rapid systemic clearance, instability, unsatisfactory cellular uptake, and low therapeutic potency [320–322], which warrants strategies to overcome these challenges. High doses of PhCs might not be safe and well tolerated, and generally cannot be administered to overcome the low bioavailability of these compounds, which underscores the necessity of utilizing a DDS to circumvent this challenge [320]. Regarding these hurdles, liposomes emerge as a well-suited platform for encapsulating PhCs, incorporating hydrophobic (e.g., terpenoids [323] and flavonoids [324]), hydrophilic (e.g., glycosides [325]), and amphiphilic (e.g., tannins [326] and saponins [327]) PhCs, thereby improving their bioavailability.

In the context of lung cancer, researchers harnessed various natural compounds (Table 3), with a focus on curcumin (CUR) and quercetin (QCT). CUR is a polyphenolic compound found in *Curcuma longa* (turmeric) [328], with a multitude of therapeutic potentials [329–333]. In lung cancer, the liposomal formulation of CUR attenuated epithelial cell adhesion molecule (EpCAM) and estrogen receptor alpha, which pertain to lung cancer cell survival and invasion [334], respectively. Moreover, the formulation repressed A549 cell proliferation, colony formation, and migration [335]. CUR and piperine, one of the main phytoconstituents extracted from *Piper nigrum* (black pepper) [336], were co-encapsulated inside liposomes and then surface-functionalized with the tumor-homing peptide, iRGD. The surface-modified preparation demonstrated superior results in vitro and in vivo, as indicated by increased tumor accumulation and internalization, inhibited tumor cell proliferation, and consequent tumor size reduction, compared to unmodified counterparts [337]. In another study, researchers observed that iRGD-modified liposomes co-encapsulated with CUR and piperine repressed the VEGFR2/P38/MK2 signaling pathway, which is implicated in cell migration and angiogenesis [338], thereby attenuating neovascularization and tumor growth [339]. Co-treatment with CUR and the chemotherapeutic agent gefitinib using a liposomal platform exhibited augmented cell internalization and lung cancer growth inhibition [340].

**Table 3:** Liposome-encapsulated phytochemicals investigated for lung cancer therapy

Phytochemical name	Source	Study type	Fabrication method	Surface modification (s)/coating	Study result(s)	Ref.
6-Gingerol (6G)	One of the phenolic compounds of *Zingiber officinale* (ginger) [348].	In vitro and in vivo	Thin film hydration method	Unmodified	6G liposomes (EE of 97.2%) exhibited potent anti-neoplastic, anti-migratory, and apoptosis-inducing effects against A549 cells. In a tumor-bearing animal model, liposomal 6G hindered weight loss and preserved the lung architecture, akin to normal lung tissue.	[349]
		In vitro	Thin film hydration method	PEGylated	6G co-encapsulated with PTX (EE: 95.20 ± 1.78% for 6G; 79.69 ± 2.3% for PTX) induced apoptosis and cell cycle arrest in lung cancer cells, alongside sustained release of the cargoes.	[350]
Baicalin (BA)	Fractionated from the roots of *Scutellaria baicalensis* [351].	In vivo	Effervescent dispersion and lyophilization (freeze-drying) method	Unmodified	BA liposomes reached desirable properties, such as EE of 90.47 ± 0.93%, particle size of 131.7 ± 11.7 nm, excellent shelf-life and stability (up to 12 months), and following IV administration, reached the highest concentration in the lungs, compared to other organs and free BA, in addition to no toxicity toward the lungs. The formulation elicited superior anti-tumor properties and the longest survival span (25.90 ± 0.53 days vs. 17.30 ± 0.47 days) compared to the BA solution in the orthotopic lung cancer model.	[352]
		In vitro and in vivo	Thin film hydration method	Unmodified	Co-encapsulated BA and CUR liposomes (EE of 94.06% for BA and 97.23% for CUR) improved the bioavailability and outperformed each compound alone (67.8% and 51.9% higher than liposomal CUR and BA, respectively) in cytotoxicity against A549 cells. Resulted in a reduction in tumor size compared to PBS.	[353]
Epigallocatechin gallate (EGCG)	Found in green tea [354].	In vitro	Ethanol injection calcium acetate method	Modified with HA and ε-poly-L-lysine	Modification had no negative effect on EE (94.71% for modified and 94.71 ± 0.03% for unmodified), decreased the liposome size (159.1 ± 4.2 nm vs. 165.4 ± 1.4 nm), increased the EGCG’s overtime retention and cellular uptake compared to unmodified liposomal EGCG, in addition to eliciting the highest cytotoxicity compared to free EGCG and unmodified preparation.	[355]
Berberine (BBR)	A natural alkaloid fractionated from barberry (*Berberis vulgaris)* [356].	In vitro	Thin film hydration method	Coated with conjugated chitosan (CS)-HA	Liposomes co-loaded with BBR (EE of 21.0%) and DOX (EE of 64.0%). Particle size increased sequentially from 312 nm (blank) to 337 nm (BBR-loaded), 362 nm (co-loaded), and 690 nm post-coating. The formulation achieved higher cellular uptake and cytotoxicity toward lung cancer cells, presumably mediated by targeting CD44 receptors on A549 cells.	[357]
Magnolol (MAG)	Fractionated from *Magnolia officinalis* [358].	In vitro and in vivo	Thin film dispersion combined with the pH gradient method	Modified with chondroitin sulfate	(2:1) ratio of BBR (EE of 78.92%) to MAG (EE of 91.72%) was encapsulated inside liposomes, which was observed to induce the highest synergistic antineoplastic effect. The formulation maintained the plasma concentration of BBR and MAG, achieved enhanced blood stability and circulation time, attenuated side effects, and reached the highest tumor accumulation and the lowest tumor size.	[359]
Resveratrol (RSV)	Isolated from grapes, peanuts, and red wine [360].	In vitro and in vivo	Thin film hydration method	Unmodified	RSV liposome (EE of 74.15%) attenuated cell proliferation and migration, and down-modulated IL-1β and IL-18 at the tumor site and systemically, thereby balanced TME’s cell disequilibrium, which was hypothesized to be correlated with RSV’s ability to repress TLR4/NLRP3 inflammasome-associated genes, ultimately leading to apoptosis and impeding LLC tumor growth.	[361]
Apigenin (API)	Found in chamomile, parsley, oranges, and garlic [362].	In vitro and in vivo	Thin film hydration method	Coated with a Vit E derivative, TPGS to act as a solubilizer and MDR-reducer	Liposomal API achieved higher cellular uptake compared to the free drug. Co-treatment with liposomal API alongside the anticancer drug tyroservatide, outpaced each treatment group alone in suppressing lung cancer cell viability, in addition to the robust antitumor effect of the combination observed in the mouse model.	[363]

*CUR* Curcumin, *DOX* Doxorubicin, *HA* Hyaluronic acid, *IL* Interleukin, *PTX* Paclitaxel, *TME* Tumor microenvironment

QCT is a flavonol found in various fruits and vegetables (e.g., apples, berries, dill, fennel, onions, okra, and coriander [341]). However, it suffers from negligible bioavailability (< 10%) [342]. To overcome this, RGD-modified liposomes encapsulating QCT achieved an EE of approximately 90% and a size of 93.4 ± 7.2 nm, which was deemed suitable for EPR-mediated tumor accumulation. Apart from passive targeting, RGD-mediated active targeting further reinforced tumor accumulation and subsequent antitumor effects [343]. Jenitha et al. explored the impact of the molar ratio of liposomal QCT composition on size, EE, and cytotoxicity against lung cancer cells. The results indicated that a higher phospholipid to cholesterol ratio (7:2:1 vs. 5:4:1 for phospholipid: cholesterol: QCT ) resulted in a reduction in size, an increment in EE (90.96 ± 0.50% vs. 80.74 ± 0.39%, *p* ≤ 0.01), and enhanced toxicity toward A549 cells [344]. This emphasizes the importance of structural composition, which should not be overlooked.

In another study, the effectiveness of QCT liposomes (Lipo-Que) was compared to a liposomal formulation of a chemotherapeutic agent, sorafenib (Lipo-Sorafenib). Similar to the previous study, liposomes with a 7:2:1 molar ratio were fabricated and administered intravenously in a benzo [a] pyrene-induced lung cancer murine model. Lipo-Que outperformed free QCT in treating malignant lung nodules; however, the therapeutic efficacy of Lipo-Que (79.29 ± 0.55%) was comparable to Lipo-Sorafenib (84.96 ± 0.56%), although Lipo-Sorafenib showed superior yet statistically insignificant results. Both Lipo-Sorafenib and Lipo-Que exhibited similar therapeutic outcomes in treating liver metastasis, as no metastatic nodules were found in liver tissue [345]. This is an important result, indicating the head-to-head potential of PhCs to combat cancer compared to chemotherapeutic drugs.

In a study by Al-Samydai et al., unlike QCT-loaded liposomes that showed no detectable cytotoxicity against A549 cells, co-loading of QCT with gallic acid (GA; a phenolic acid abundant in various fruits and vegetables [346]) resulted in amplified and synergistic cytotoxicity. Moreover, GA exhibited superior cytotoxicity in both free and encapsulated forms, compared to QCT, which was assumed to be associated with the acidic nature of GA that can elicit robust apoptosis [347].

#### Clinical translation and industrial scaling of phytochemicals: challenges and opportunities

**Fig. 6:**
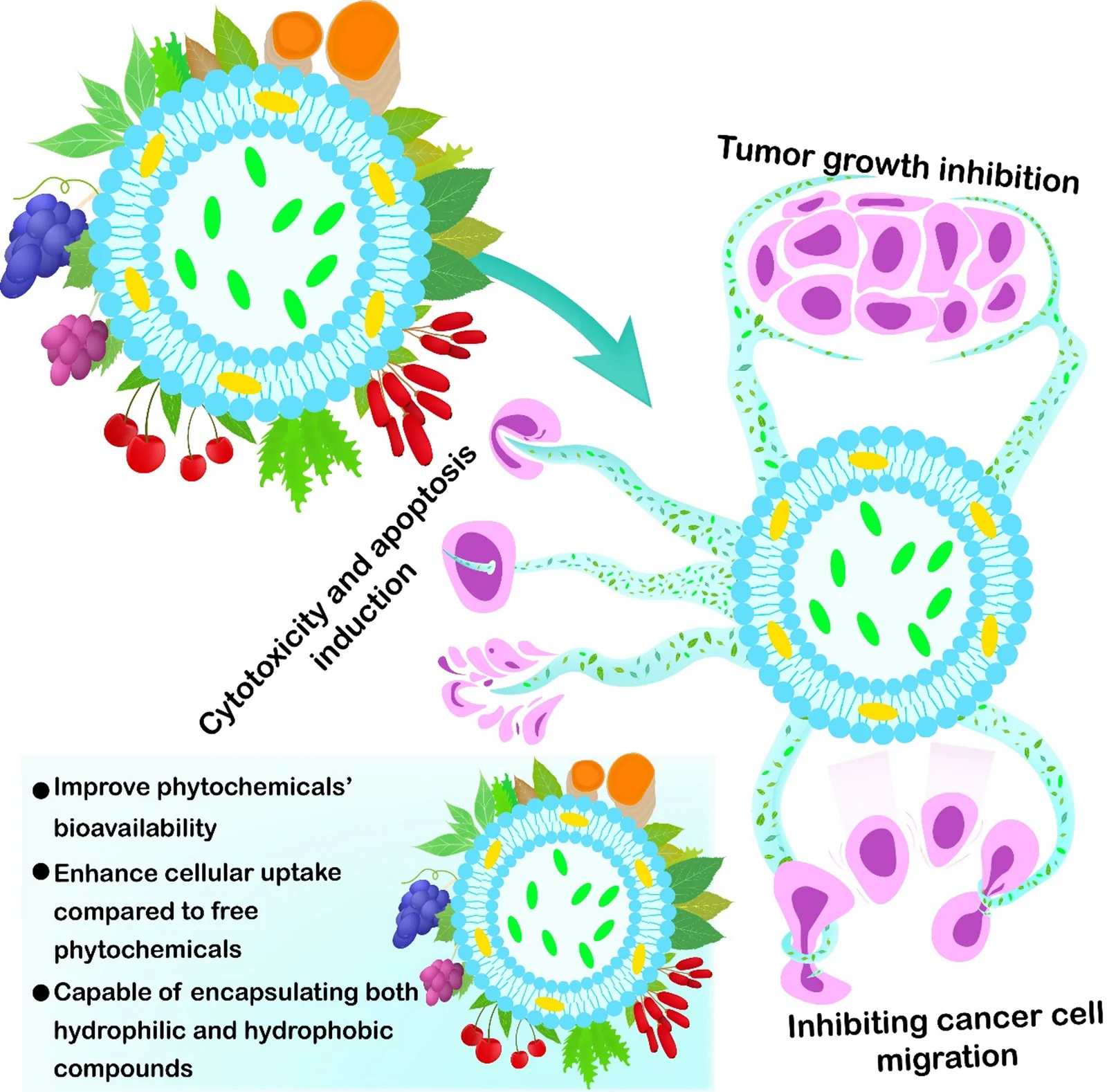
Pleiotropic effects of phytochemicals in lung cancer oncotherapy. By enhancing bioavailability and cellular uptake, liposomal platforms have augmented the therapeutic potential of natural compounds

**Fig. 7:**
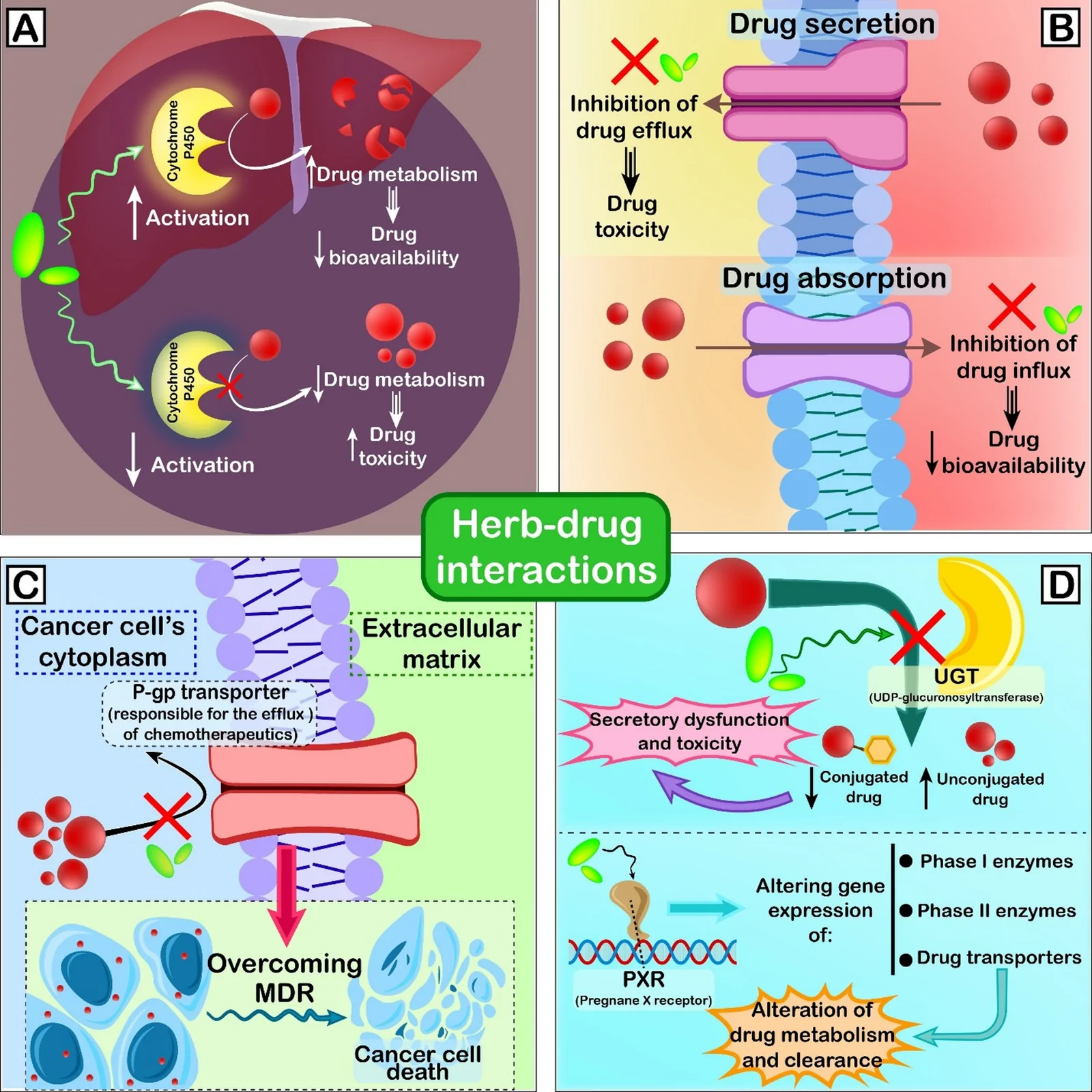
Underlying mechanisms of phytochemical (PhC)-mediated herb-drug interaction (HDI). **A** PhCs can alter the effective concentration of drugs by affecting the activity of Phase I metabolic enzymes (e.g., cytochrome P450). Activation of these enzymes accelerates drug metabolism, thereby attenuating drug bioavailability. However, inhibition of these enzymes impairs drug metabolism and leads to drug toxicity. **B** By affecting drug transporters responsible for absorption and excretion, PhCs can either reduce bioavailability by restricting drug absorption or induce toxicity by impairing excretion. **C** HDIs can be exploited to overcome MDR. Inhibition of chemotherapeutic drug efflux pumps on the surface of cancer cells (e.g., P-gp) prevents drug efflux, leading to intracellular drug accumulation and increased cytotoxicity. **D** PhCs can interfere with drug glucuronidation by affecting Phase II metabolic enzymes such as UGT, thereby disrupting drug clearance; also, by affecting the PXR, PhCs can alter the gene expression of transporters and Phase I and II metabolizing enzymes, which affects the metabolism and excretion of drugs

Despite all the listed advantages brought about by natural compounds and the capability of liposomes in augmenting their therapeutic potential (Fig. 6), the future of natural-based oncotherapy is facing several challenges. One major bottleneck in applying PhCs alongside routine therapeutic regimens lies in their inherent propensity to induce herb-drug interactions (HDIs) [364], which can jeopardize the patient’s health and therapeutic outcome. Herbal formulations can induce HDIs through:

1. Boosting/suppressing the function of Phase I metabolizing enzymes (main driver of HDI): through two mechanisms: (a) by augmenting the activity of the cytochrome P450 (CYP) enzyme family (e.g., hyperforin, the active constituent of *Hypericum perforatum*), the effective drug concentration can fall below the therapeutic threshold. (b) By inhibiting the CYP family (e.g., grapefruit juice, curcumin, and BBR), the systemic drug concentration can reach toxic levels [365] (Fig. 7A).
2. Interacting with two types of drug transporters that are responsible for drug absorption and secretion (Fig. 7B). Although it can result in a synergistic effect and prevent MDR (by improving oncotherapeutic drug bioavailability and preventing drug efflux [366]) (Fig. 7C), the risk of drug adverse effects and toxicity can be heightened due to up-modulation of absorption or reduction in secretion. On the other hand, by impeding absorption or increasing secretion, the drug bioavailability can fall below the therapeutic window and lose its effectiveness [367].
3. Interaction with Phase II metabolizing enzymes, e.g., UDP-glucuronosyltransferase (UGT) [368], and alteration in pregnane X receptor (PXR) nuclear factor that regulates drug transporters, Phase I, and II metabolizing enzyme expression can influence drug metabolism and clearance [369] (Fig. 7D).

Cancer patients, especially elderly populations, often take several drugs [370], predisposing them to an elevated risk of HDIs. To overcome these challenges, it is imperative that future studies evaluate and report the capability of NDDSs, such as liposomes, to circumvent HDI by attenuating off-target PhC interactions, reducing hepatic concentration and metabolism, and more importantly, by leveraging targeted delivery of PhCs, it can result in precise alteration in drug transporters, such as inhibiting P-gp at the cellular level in cancer cells, which can prevail over MDR [371].

Another obstacle in the clinical translation of natural compounds is the need for a secure source to ensure a constant supply of PhCs. Extracting PhCs only from plants is influenced by circumstances such as climate change and global disasters [372], threatening a sustainable supply of herbal resources. Moreover, large-scale cultivation and harvesting of plants can result in deforestation, waste of water, and weaken the soil [373]. Apart from these unpredictable and detrimental elements, there are modifiable factors such as light [374], plant growth medium composition [375, 376], fertilization [377, 378], and farming techniques [379, 380] demonstrated to affect PhCs biosynthesis, which changes from plant to plant [381], and can be optimized to maximize PhCs yield. Harvesting methods, extraction techniques, and stockpiling parameters can also affect PhCs content [382].

To circumvent the obstacles in providing high-value and expensive PhCs for clinical applications, especially those with complex structures, researchers can leverage the potential of synthetic biology to produce herbal constituents [383, 384]. By incorporating plant biosynthesis genes into yeasts (e.g., *Saccharomyces cerevisiae*) and bacteria (e.g., *Escherichia coli*), researchers have synthesized various PhCs (e.g., RSV, CUR, and BBR) or their derivatives [385–388]. By leveraging synthetic biology in microorganisms, researchers and pharmaceutical scientists can produce PhCs at laboratory or industrial scales, without being affected by environmental factors such as seasonal and daily changes in light, temperature, weather, and humidity. This is presumed to considerably decrease manufacturing costs and increase the production rate. However, to optimize and boost production yields, plant biosynthetic cascades or alternative synthetic routes, strategies to enhance enzymatic productivity and selectivity, and approaches in scale-up production need to be elucidated, which indeed demands tremendous multi-disciplinary expertise [383].

Another reasonable approach is to focus on PhCs that meet two key criteria: possess numerous supply resources and can be synthesized at an industrial scale. To the best of our knowledge, one of the candidates is lycopene, a carotenoid present in tomatoes and various bacteria and fungi, which can be obtained using synthetic biology in *E. coli* and *S. cerevisiae* [389]. For industrial production, lycopene can be chemically synthesized harnessing petrochemicals [390], extracted from tomato waste [391], and produced by natural lycopene-stockpiling microorganisms (e.g., *Blakeslea trispora* fungi) [389], which underscores the large and accessible sources for streamlining a more tangible clinical translation. Another applied option, especially if future research can reduce its final cost, is astaxanthin (AST), a carotenoid present in algae and yeasts. *Haematococcus pluvialis* is a microalga that intracellularly stores AST (~ 4–7 g in 100 g of dry *H. pluvialis*) [392, 393]. AST is regarded as one of the most potent antioxidants/anti-inflammatory agents, with one of the highest oxygen radical absorbance capacity (ORAC) scores, 100- to 500-fold higher than that of vitamin E (⍺-tocopherol) and 10-fold higher free radical scavenging activity than other carotenoids (α-carotene, β-carotene, lycopene, and lutein) [394]. Due to structural differences, synthetic AST has attenuated antioxidant potency (14- to 55-fold) compared to natural AST [395]. Apart from the high cost of natural top-grade AST, which indeed demands broadening of the current landscape of natural AST aquaculture at an industrial scale, it suffers from low bioavailability. Numerous studies have strived to enhance the cultivation yield, attenuate production cost, design large-scale cultivation settings, and improve the bioavailability of AST [396–404]. By advancing NDDSs, such as liposomes, the bioavailability of AST following oral or IV administration could be increased.

Based on trends in conducted clinical trials, plant-derived remedies and their representative PhCs have been frequently harnessed to alleviate symptoms (nausea and vomiting [405], fatigue [406, 407]) or oncotherapy side effects (e.g., chemo-induced cardiotoxicity [408], radiotherapy-mediated dermatitis [409]), rather than acting as the main therapeutic regimen, which raises the question of whether they can transcend supportive care or remain merely palliative. However, several established chemotherapeutic drugs, such as vincristine, paclitaxel, topotecan, and irinotecan, are isolated from medicinal plants [410], highlighting the importance of chemical exploration and engineering of herbal compounds for oncotherapy, and reflecting that PhCs can offer far greater clinical utility than serving as a mere palliative strategy.

Another impeding hurdle in the clinical translation of PhCs is that, unlike established drugs, the lack of strict, universally approved regulatory and standardization protocols for dosage and quality of herbal-based remedies, in addition to their inherent diversity in chemical concentrations, can lead to adverse, unpredictable drug reactions or suboptimal therapeutic effects. Consequently, extensive preclinical, clinical, systematic review, and meta-analysis studies are required for a safe clinical translation [382, 411].

The ability to reshape the gut microbiota is another arrow in the quiver of natural products [412–414]. Gut and lung microbiome dysbiosis has been found to correlate with lung cancer via disrupting metabolic homeostasis and the immune system, and eliciting pro-inflammatory conditions [415], in addition to mediating oncotherapy resistance [416]. Nanomedicine, especially microbiota-targeting NDDSs, serves as an amplifier of therapeutic efficacy for rebalancing the gut microbiota to confront cancer [417, 418]. We encourage researchers to harness liposomes and other NDDSs, with a higher focus on microbiota-targeted/responsive delivery, intestine-retentive platforms, and pH-mediated and colon-specific drug release [419–422], for altering the microbiome, alone or in combination with immunotherapy and chemotherapy.

**Fig. 8:**
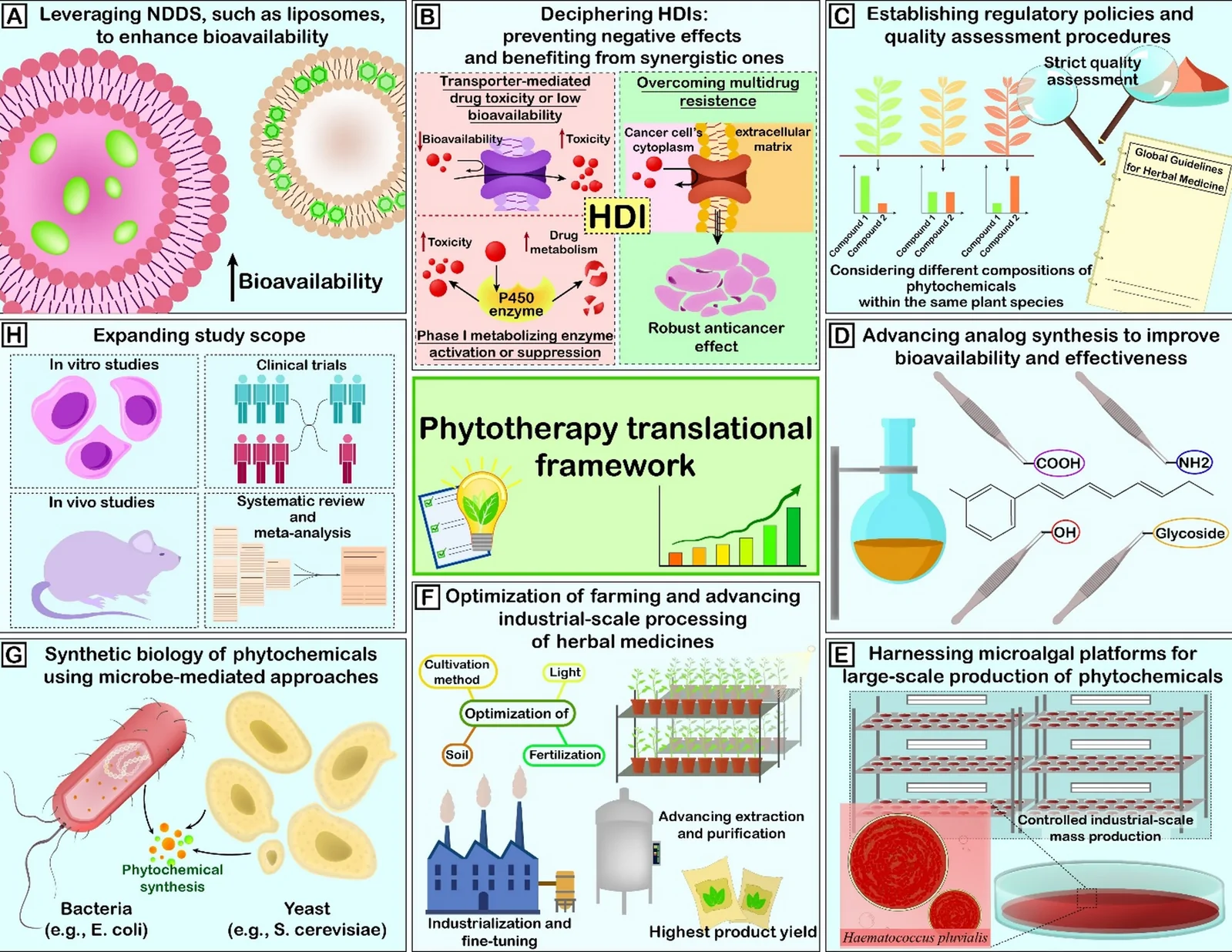
Recommendations for the successful bench-to-bedside translation of phytochemicals (PhCs) in cancer therapy. **A** Application of NDDSs, including liposomes, to improve the bioavailability and stability of plant-derived compounds. **B** Implementation of rigorous herb-drug interaction (HDI) studies to identify potential adverse interactions and exploit synergistic effects with conventional anticancer therapies. **C** Establishment of robust quality control and monitoring frameworks to standardize plant-derived products and minimize variability in PhC composition. **D** Development and screening of chemical analogue libraries to identify novel derivatives with improved pharmacokinetic properties and greater therapeutic efficacy. **E** Utilization of microalgae-based production systems for the scalable and sustainable biosynthesis of rare PhCs. **F** Optimization of cultivation conditions and downstream processing strategies to improve product quality, consistency, and yield. **G** Application of microbial synthetic biology approaches as sustainable and programmable platforms for PhC production. **H** Expansion of research efforts from mechanistic in vitro studies and in vivo validation to well-designed clinical trials, ultimately generating high-level evidence through systematic reviews and meta-analyses to facilitate clinical translation

At the end, it is recommended that researchers focus more on in vivo studies, analog fabrication, and chemical engineering of PhCs for improving their potency [423], and to evaluate the potential of liposomal NDDSs in boosting the bioavailability, hampering harmful HDIs, and enhancing therapeutic efficiency. Moreover, a head-to-head comparison of PhCs with chemotherapeutic drugs (as studied by Jenitha et al. [345]) can further authenticate PhCs’ place amongst cancer treatments. Advancing the landscape of manufacturing PhCs via synthetic biology or harnessing microorganisms at an industrial scale can underpin a strong base to provide PhCs for an accessible and cost-effective clinical application. Moreover, strict policies regarding herbal-based drug quality assessment, manufacturing and industrialization, and market supervision [424] should be established to pave the way for standardization and clinical translation (Fig. 8).

### Nucleic acid-based therapies

Nucleic acid-based approaches are another ever-expanding sector of oncotherapy. These strategies function either by activating or suppressing gene expression [425], or by introducing a specific gene sequence encoding tumor cell proteins for induction of an immune response (as discussed earlier in the cancer vaccine section) [251]. However, conventional gene therapy faces obstacles such as low transfection efficiency (TE) [426], peripheral disintegration by nucleases, lack of specific targeting, blood clearance, and intracellular degradation via the endolysosomal route [427, 428]. NDDSs have garnered tremendous attention by providing protection against enzymatic degradation and increasing TE through targeted delivery and augmented cellular internalization.

Small interfering RNA (siRNA) is a noncoding RNA that is deemed a powerful tool in combating various gene-related diseases and is based on the disintegration of messenger RNA (mRNA), thus silencing translation. However, non-encapsulated siRNA is prone to degradation and induction of undesired off-target effects [429], alongside diminished cellular entry and transfection [430]. Liposomal NDDSs can address all these obstacles, as they enhance cellular uptake, protect siRNA from degradation, and minimize off-target toxicities via passive or active targeting.

In lung cancer models, a cationic liposome was chosen to form an siRNA lipoplex for silencing survivin, a protein overexpressed in cancers that inhibits apoptosis [431]. Administration of the siRNA-lipoplex in A549 cells down-modulated survivin, escalated apoptosis, and induced cell cycle arrest [432]. Similarly, administration of cytochrome P4501A1 (CYP1A1) siRNA escalated apoptosis and repressed colony formation in vitro and hindered tumor growth in vivo [433].

Harnessing active targeting, Shi et al. used CTX to fabricate TGF-β1 siRNA-loaded immunoliposomes and evaluated cellular uptake kinetics and cytotoxicity. The results were indicative of boosted cellular uptake and anti-proliferative effects in vitro, and noticeable tumor accumulation and size reduction in vivo [434]. Compared to non-encapsulated siRNA, transferrin-functionalized liposome-loaded human antigen R (HUR; a protein overexpressed in a multitude of cancer types that contributes to tumor cell proliferation and invasion [435]) siRNA showcased potent tumor-homing behavior, HUR repression, tumor growth inhibition, and anti-metastatic properties [436]. In a more sophisticated study, liposomal co-delivery of VEGF siRNA and etoposide remarkably hindered angiogenesis and synergistically repressed tumor growth [437]. Similarly, co-encapsulated PTX and survivin siRNA liposomes quenched the survivin level and achieved superior cell uptake and cytotoxicity [438].

Similar to siRNA, microRNA (miRNA) is a noncoding RNA sequence that can target and decompose mRNA, thus silencing gene expression [439]. The diagnostic and prognostic potential of miRNAs has been the subject of numerous investigations [440–444]. Despite their potential therapeutic capabilities, they suffer from a short half-life and may instigate off-target side effects, such as gene silencing in healthy tissues [445]. To cope with these challenges, researchers harnessed NDDSs, such as liposomes. To this end, a cationic liposome was fabricated for the delivery of miR-143, a miRNA that represses tumor progression [446], to tumor sites, and when administered, remarkably repressed tumor growth and metastasis, compared to the chemotherapeutic drug, Cis [447]. By advancing the design through surface modification, a folate-functionalized liposome was tailored to co-encapsulate Cis alongside miR-219a-5p [162], an miRNA that showed potential in circumventing Cis resistance [448]. The liposomal formulation favorably protected the miRNA, enhanced cellular uptake, overcame Cis resistance, repressed tumor cell migration, and incited apoptosis [162]. In another innovative study, a dual-peptide-functionalized liposome for co-delivery of miR-34a, a tumor- and cancer stem cell-suppressive miRNA [449], and the gap junction rebalancing molecule, all-trans retinoic acid (ATRA), was designed. The in vitro assay demonstrated remarkable intracellular accumulation and cytotoxicity that were consistent with in vivo results of reinforced tumor accumulation and tumor size reduction. Researchers believed that the ATRA-mediated increase in gap junction proteins facilitated the intercellular transport of miR-34a and augmented therapeutic potential [450].

Short hairpin RNA (shRNA) is another tool for repressing gene expression [451]. Chowdhury et al. designed a liposomal formulation to co-deliver docetaxel (DTX), loaded within the liposomal bilayer, together with a PFKFB3-targeting shRNA plasmid, electrostatically complexed onto the positively charged liposomal surface. Silencing PFKFB3, an overexpressed glycolytic regulator in NSCLC, obviated the metabolic support required for tumor growth and was also associated with reduced P-gp activity. As a result, combining DTX with PFKFB3 knockdown produced markedly enhanced cytotoxicity in A549 and H460 cells compared to single-agent treatments [452].

Despite the potential of liposomes in improving delivery and protecting nucleic acids in circulation, several obstacles remain unresolved. The gene silencing effect of post-transcriptional nucleic acids is not permanent, because they exclusively affect the mRNA, not the encoding DNA. For example, siRNA silences genes merely for days to weeks [453], which may not be suitable for a chronic condition, such as cancer. Cationic liposomes, which are widely used in gene delivery, are prone to react with serum proteins and form a protein corona [454], which can negatively affect the delivery. Endolysosomal degradation is another obstacle in nucleic acid delivery [455], and applicable strategies must be taken into account for evading this pathway. Furthermore, extensive intratumor heterogeneity in human cancer gene expression is another major challenge [456], which can create uncertainties regarding the effectiveness of the single-gene-silencing strategy and warrants personalized design of gene silencing approaches. To transcend these intertwined limitations, it is imperative for future research to rationally design NDDSs to minimize or take advantage of nano-bio interactions, evade endolysosomal degradation, and circumvent intratumoral genetic heterogeneity.

### Phototherapy

Lung cancer, long perceived as a hard-to-treat malignancy, has benefited from continual therapeutic innovations [457]. Yet, MDR [458], coupled with the notoriously hard-to-access tumor niches, particularly those nestled within the upper bronchi, continues to challenge conventional modalities and has prompted scientists to explore multi-modal, locally activatable treatments. One such co-therapy is the use of light to battle cancer. This approach is particularly useful for endobronchial or centrally located lung cancers, where the controlled delivery of light is feasible [459]. In this strategy, specific wavelengths of light are used to activate the photosensitizers (PSs) or the photothermal agents (PTAs), inducing targeted tumor destruction through ROS generation or photothermal heating, respectively [460]. To date, several PSs and PTAs have been approved for lung cancer [461, 462]. However, conventional phototherapies face numerous shortcomings, including limited stability, off-target thermal damage, and rapid degradation in systemic circulation. Moreover, the hypoxic environment characteristic of lung tumors compromises the efficacy of oxygen-dependent photodynamic therapy (PDT) [463]. Such limitations have led to the application of NDDSs to enhance phototherapy performance [464].

Among these nano-platforms, liposomes offer several advantages that make PTT and PDT more favorable. In this section, we introduce the advantages of liposome-enabled photothermal and photodynamic lung cancer therapy. Liposomal delivery can improve the stability of PSs and PTAs in systemic circulation and enable preferential tumor accumulation via the EPR effect or ligand-directed active targeting. Liposomes can be engineered to release their payload in response to light irradiation, enabling spatiotemporal drug delivery at the target site. Moreover, the co-delivery capacity endowed by liposomes has enabled the engineering of formulations capable of targeting mitochondria, further consolidating the efficacy of phototherapy.

#### Photothermal therapy

**Fig. 9:**
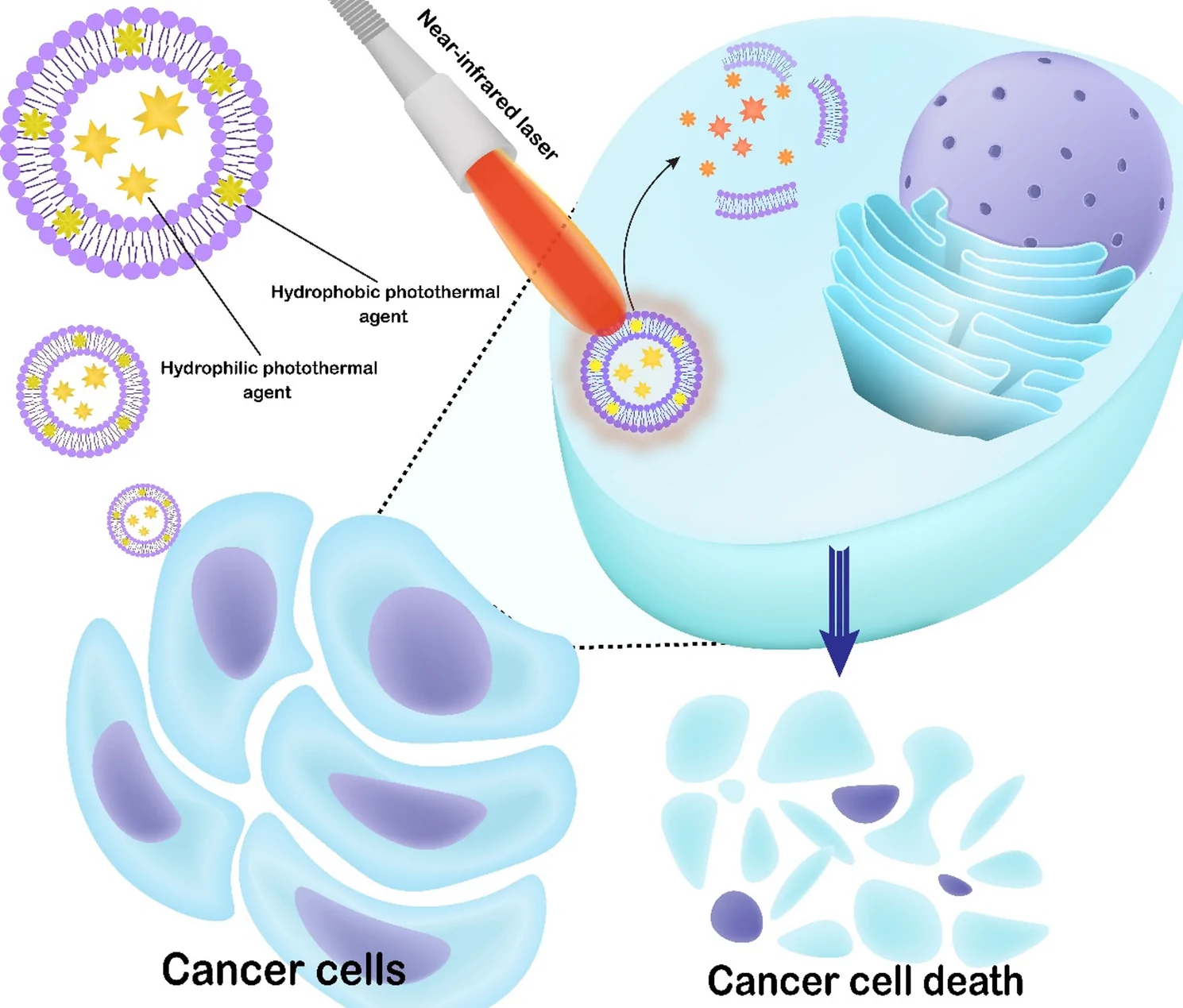
Mechanism of liposome-assisted PTT. Liposomes can enhance cellular uptake and delivery of PTAs, and upon cellular entry, PTAs absorb near-infrared (NIR) light and convert it to heat, leading to hyperthermia-induced cell death

Photothermal therapy (PTT) is a non-invasive stratagem in cancer therapy that eliminates malignant cells by means of heat. This method is based on leveraging PTAs that can emit absorbed light as heat, thus killing tumor cells (Fig. 9). When coupled with nano-platforms and active targeting, it will selectively kindle tumor tissues, leaving normal and healthy cells unharmed [465]. Moreover, the addition of PTA agents to nano-platforms can bestow the ability of stimuli-responsive behavior, instigating endolysosomal escape─ a system that disintegrates endocytosed molecules, acting as a major obstacle in drug delivery [466]─ and abrupt drug release in the desired milieu [467].

Regarding lung cancer, researchers scrutinized the anticancer capabilities of PTT. To enhance tumor accumulation and attenuate the unwanted toxic effects, DOX and indocyanine green (ICG; a dye with photo-responsive behavior [468]) co-loaded liposomes were fabricated (EE of 52.2% for DOX and 67.1% for ICG). In vivo administration of this formulation in an A549 model achieved tumor accumulation and, after photo-irradiation, demonstrated tumor size reduction. However, the absence of tumor targeting resulted in unfavorable liver and intestine accumulation [469]. In a 3D lung cancer cell model, gold NPs (GNPs) and pheophorbide A co-loaded liposomes were able to induce PTT and apoptosis through activation of proapoptotic proteins, e.g., BCL-2-Associated X Protein (BAX) and caspase-9 [470]. Liposome-integrated boron-fluorine-dipyrrole (BODIPY) dye and surface-affixed small GNP rectified the limitations of this compound, such as poor solubility and aggregation, yielded standout PTT-properties as a result of dual-agent packing, and showcased laser-responsive drug release capabilities and promoted the PTT-mediated cell apoptosis in an NSCLC cell line [471].

In an A549 cell line model, by leveraging the EGFR-targeting ability of the GE11 peptide, a GE11-modified liposome was tailored and co-encapsulated with CUR and ICG. This formulation achieved precise targeting and, under NIR irradiation, elicited hyperthermia-induced cancer cell death [472]. Patel et al. artfully depicted the potential of nanotechnology by designing a dual folate and GE11-modified IR780 dye-loaded thermo-sensitive liposome, which wrapped gold nanorods coated with a DOX-integrated mesoporous silica shell. This NP demonstrated boosted targeted delivery to A549 cells and, upon cellular uptake, instigated laser-triggered endosomal escape and DOX release. Furthermore, in the presence of laser irradiation, the formulation induced heightened ROS production, glutathione (GSH) depletion, and, collectively, the combined chemo-PTT resulted in 11-fold higher toxicity than free DOX [473].

#### Photodynamic therapy

**Fig. 10:**
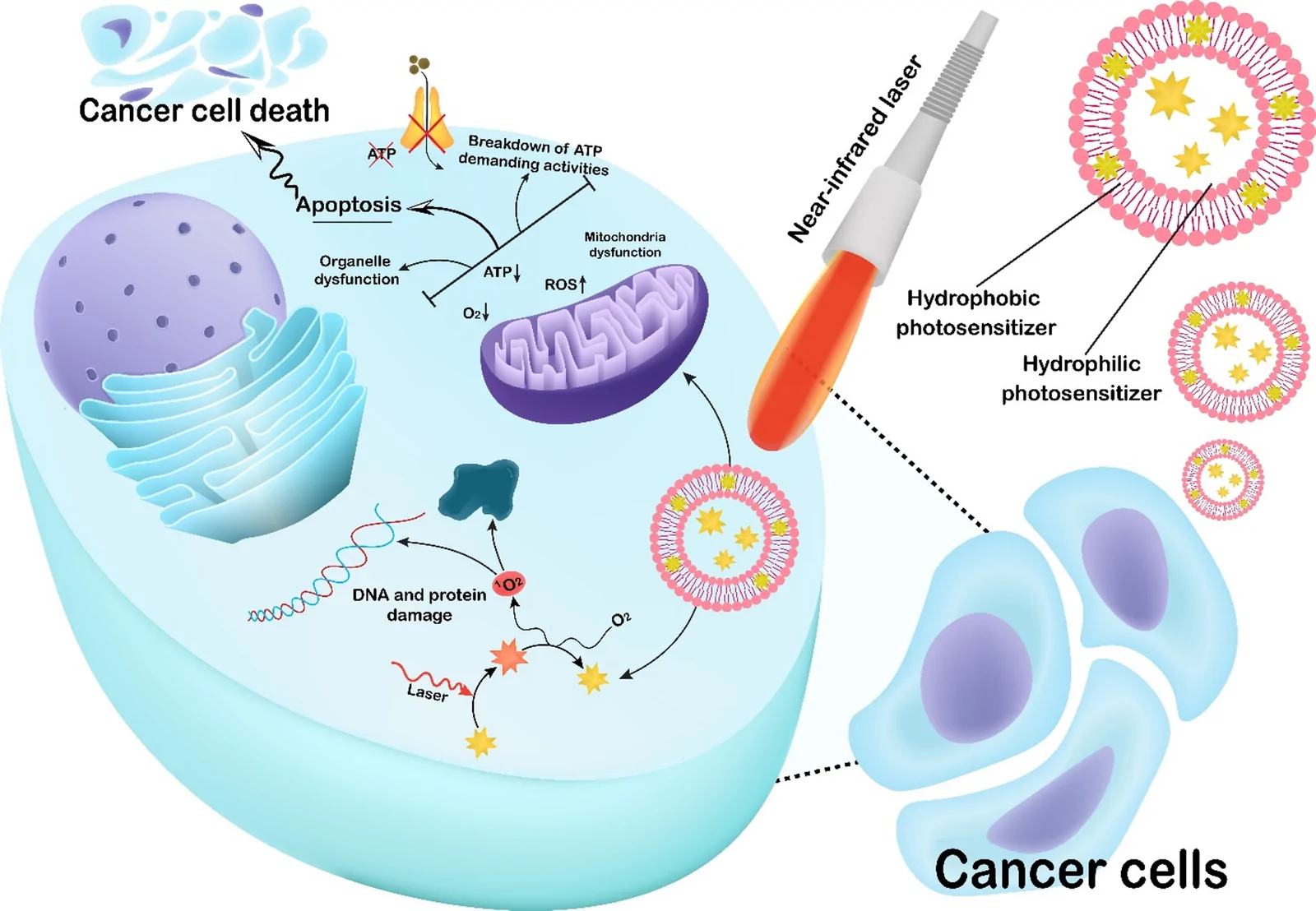
Mechanism of liposome-assisted PDT. Following cellular uptake, the PS is excited by NIR light, generating ROS, which can damage DNA and proteins. Moreover, PDT consumes O_2_, thus attenuating intracellular oxygen and inducing mitochondrial dysfunction, which results in diminished ATP synthesis, ultimately impairing ATP-demanding activities (e.g., membrane pumps). These changes lead to homeostasis imbalance, eventually triggering cell death

Similar to PTT, PDT is a non-invasive method that relies on light to eliminate cancer cells. The PDT cytotoxic effect is mediated through an oxygen-dependent pathway that is based on utilizing a PS that, following light absorption, produces ROS [474], which damages DNA, proteins, and cellular organelles (Fig. 10) [475]. PSs can also instigate dysfunction in mitochondrial respiration via depleting intracellular oxygen, resulting in attenuation of ATP levels and breakdown of ATP-demanding activities. Eventually, these changes can lead to cell apoptosis.

**Fig. 11:**
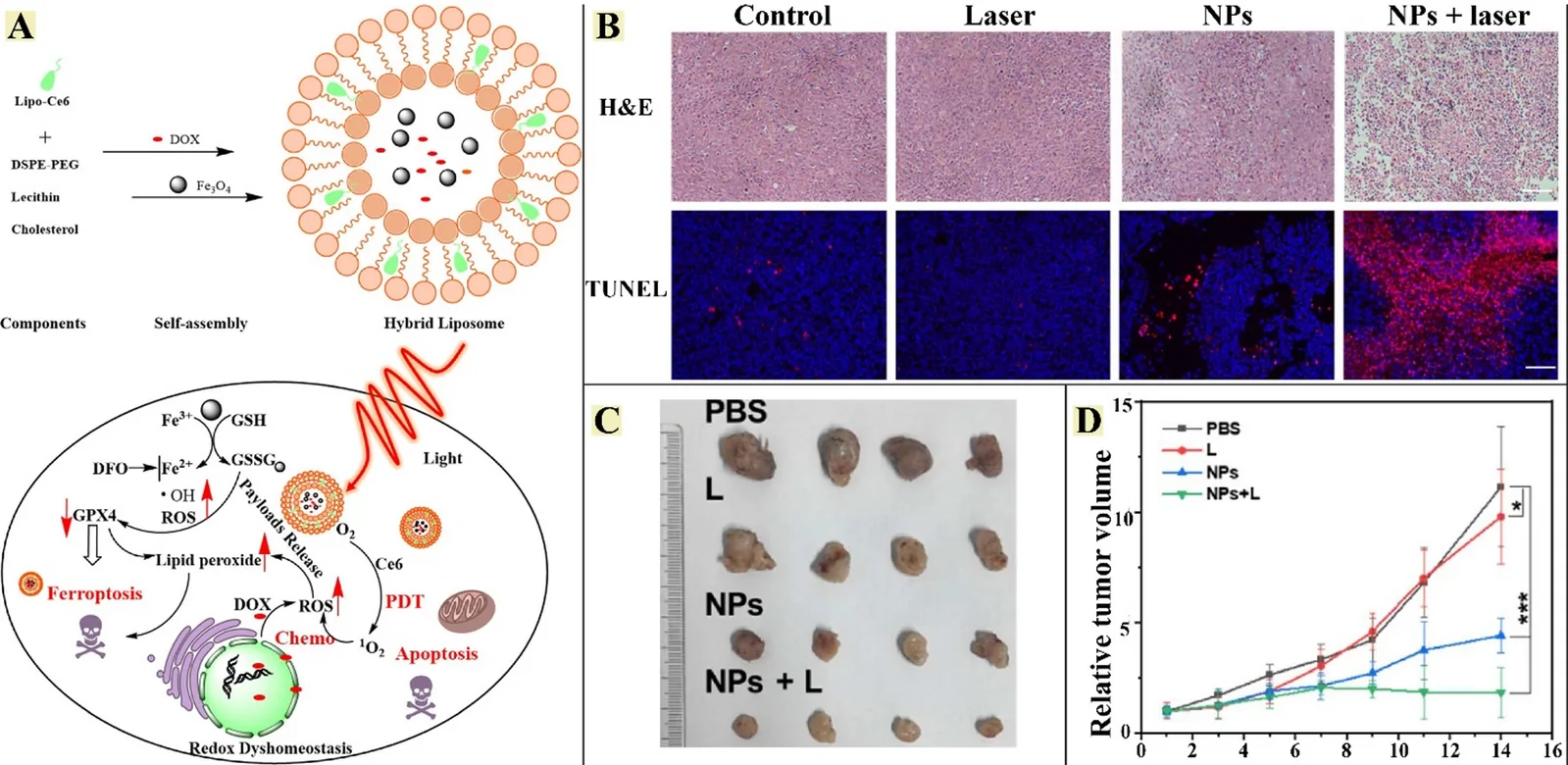
**A** A schematic illustration of the composition of the multifaceted liposome and the mechanism of PDT/ferroptosis/ chemotherapy induced by the composition. **B** H&E and TUNEL staining of the tumor tissue in different groups. TUNEL staining indicates heightened apoptosis in the NP + laser group. **C** Photograph of tumor tissue size and (**D**) Tumor growth rate in different groups. Reproduced with permission from ref [476]. Distributed under the terms of the Creative Commons Attribution-NonCommercial 3.0 Unported License

Transcending the conventional approach, Huang et al. fabricated a multifunctional Chlorin e6 (Ce6)-lipid conjugate-incorporated hybrid liposomal platform co-encapsulated with DOX and the ferroptosis inducer Fe3O4. Ce6-lipid conjugation reduced Ce6 release, mollified the Ce6 aggregation, and subsequently boosted singlet oxygen production. Upon light application, the liposomal formulation achieved remarkable outcomes in inhibiting tumor growth in an LLC-bearing animal model (Fig. 11). The results of in vitro assessment showcased synergistic ROS production via Ce6-mediated PDT, Fe3O4-orchestrated Fenton reaction and ferroptosis induction, and DOX’s ability to increase ROS production, ultimately augmenting the therapeutic potential of this multifaceted nanoplatform [476].

As mentioned before, mitochondria play a cardinal role in PDT and have garnered the attention of researchers as targets to boost PDT efficiency. Mitochondria-targeted PSs could outperform non-targeting ones in inducing cancer cell death through heightened ROS production and mitochondria-mediated apoptosis [477, 478].

By leveraging the high propensity of triphenylphosphine (TPP; a positively charged molecule) to accumulate in mitochondria, a TPP-modified liposome was designed to encapsulate two hydrophobic molecules, lonidamine (LND)─ an anticancer molecule blessed with the ability to boost PTT and PDT potential [479]─ and IR780 in the liposome bilayer. Laser application on the mitochondria-targeted formulation resulted in a rise in temperature, which incited liposomal membrane disintegration and LND release. Moreover, the IR780 resulted in PDT through the release of ROS, collectively inducing mitochondrial dysfunction in vitro and in an animal model of LLC [480]. Elsewhere, a mitochondria-affixing PS, Cy-Br, and PTX-impregnated liposome was designed to sophisticatedly intertwine PDT and chemotherapy. After A549 cell uptake, the Cy-Br-orchestrated PDT resulted in mitochondrial dysfunction and ATP attenuation, leading to down-modulation of P-gp functionality, thus promoting chemotherapeutic potential [481].

In a forward-looking study, researchers integrated protoporphyrin IX (PpIX), a hydrophobic PS with high propensity toward unsaturated phospholipids of the cell plasma membrane, into Cis-incorporated liposomes. Following in vitro chemo-PDT, the fabricated NDDS remarkably attenuated Cis-resistant A549 cell viability, compared to Cis liposome alone. The underlying mechanism of boosted chemo-toxicity was pertained to the laser-provoked PDT by plasma membrane-attached PpIX, interrupting the P-gp efflux system [482].

Phytomedicines can be leveraged as PSs [483]. Curcuminoid-encapsulated liposomes enhanced the bioavailability of the cargo and, following administration to A549 cells under blue light, produced ROS, escalated caspase-3 level, and eventually resulted in apoptotic cancer cell death [484]. To tackle the low bioavailability of BBR, liposomal BBR was fabricated, and when administered alongside laser irradiation against A549 3D cell spheroids showcased remarkable growth regression and anti-colony formation behavior, as it reduced the spheroid diameter by approximately 50% [485]. Co-encapsulated GNPs and BBR liposomes were able to exert augmented synergic PDT by BBR and GNPs in a 3D spherical lung cancer model. The designed formulation reached the highest growth inhibitory properties, although the abrupt and accelerated release of the loaded compounds can endanger storage ability and clinical translation [486].

In the study of Lehmann et al., nebulized CUR-loaded liposomal formulations incorporated with different tetraether lipids (TELs) (DPPC: TEL) were tested and compared with cholesterol (DPPC: Chol) or DOPE (DPPC: DOPE) incorporated formulations for their characteristics and PDT efficacy, to propose a candidate for a nebulizable liposomal PDT-inducer in lung cancer pulmonary delivery. Incorporation of TELs into DPPC: TEL resulted in the highest degree of membrane stability, attenuated liposomal sensitivity to oxidation, and conferred the highest EE and loading capacity compared to peers. DPPC: TEL exhibited remarkable cell accumulation and, upon incubation with A549 cells, instigated noticeable cell death [487].

Despite the allure of PDT and PTT in preclinical studies and the potential of liposomes in augmenting therapeutic efficacy and overcoming some of the main challenges inherent to phototherapy, namely poor bioavailability and suboptimal tumor accumulation [488], several issues remain unresolved in the way of the future applicability and clinical translation of phototherapy for cancer treatment. The main concerns and proposed solutions are:

1. Hindered phototherapeutic efficacy owing to inadequate tumor photo-penetration and tissue light scattering: Even with the use of the second near-infrared window (NIR-II) that leverages longer wavelengths to achieve deeper tissue penetration than the NIR-I window (1000–1350 nm vs. 700–1000 nm) [489], it can merely increase the depth by a few millimeters. Ultradeep phototherapy strategies such as harnessing optical fiber-assisted PTT, expanding laser spot size, and methods for skin tissue optical clearance to attenuate tissue light scattering can enable deeper penetration of light and enhance phototherapy efficiency [490]. Furthermore, replacing light with ionizing radiation, such as X-rays and γ-rays, can provide a highly penetrative source of energy, which is referred to as radiodynamic therapy [491]. Given that radiotherapy is one of the established treatment lines in lung cancer [492], patients can benefit from a platform that enables simultaneous and targeted radiotherapy augmentation (radiosensitization) and radiodynamic therapy via a multifunctional, tumor-targeting NDDS [493].
2. Tumor thermal resistance mechanisms: tumor cells develop thermal resistance via mechanisms such as heat shock protein (HSP) upregulation, especially when subjected to extreme heating. Researchers proposed approaches such as the combination of mild-temperature PTT with HSP inhibition or tumor starvation strategies to boost treatment efficacy [494].
3. Tumor hypoxic environment: due to imperfect and rapid vascularization, tumors suffer from hypoxia, which can incapacitate oxygen-dependent PDT. Researchers observed that by harnessing approaches such as oxygen delivery to tumor tissue, in situ oxygen production, inhibition of mitochondrial respiration, rectification of tumor abnormal vascularization, and engineering PSs to consume less oxygen, the hypoxia can be attenuated [495–497].

Considering these challenges and the insights needed to overcome them prior to study design is essential for improving therapeutic outcomes and addressing the limitations that hinder cancer phototherapy. Moreover, we recommend that researchers conduct more in vivo studies, due to the inability of in vitro studies to evaluate the drug disposition, compare the tumor accumulation of NPs and free drugs, or assess NPs’ capability to successfully surmount hypoxia to maximize PDT efficiency. We also recommend a greater focus on the application of alternative agents, such as PhCs, due to their pleiotropic behaviors, acting not only as PSs [498], but also possessing numerous antitumor properties. Furthermore, broadening the research scale by expanding the scope of translational studies that bridge the gaps between preclinical and clinical stages can facilitate clinical translation of photo-based oncotherapy.

## Inhalation-based liposomal delivery

Similar to other cancers, there are several routes for lung cancer drug delivery [499, 500]. However, unlike other types of cancer, the lungs benefit from a distinctive route, the pulmonary pathway. In light of this local non-invasive delivery method, drugs can be delivered directly to the lungs; therefore, they can reach higher concentrations while reducing unwanted systemic toxicity [501, 502]. Leveraging nanotechnology is an alternative way to lower adverse effects and drug toxicity, increase drug bioavailability, enable targeted drug delivery, and maximize anticancer activity [503, 504].

To achieve efficient pulmonary delivery, it is imperative to have a grasp of the anatomical features of lung cancer, alongside physicochemical properties that drugs and carriers must possess to reach the desired sites. Based on their location, lung malignant masses are subcategorized into two groups. Central masses, which refer to tumors that are located less than 2 cm from large airway compartments, such as the primary and secondary bronchi, and peripheral masses, which are tumors that are closer to smaller airways and subsegmental regions [505]. Importantly, it has been observed that particles with a mean mass aerodynamic diameter (MMAD) of 1 to 5 μm are deemed suitable for permeable and deep pulmonary delivery, as particles exceeding 5 μm can become entrapped in the upper airway and mouth, while particles smaller than 1 μm are at risk of being expelled during exhalation [506]. Thus, it can be concluded that based on the location of the lung cancer mass, the NDDS can be downsized or upsized to maximize drug localization.

As a well-established nanocarrier, liposomes offer superb properties in drug delivery via the pulmonary route, as their building blocks are structurally similar to pulmonary surfactants, thereby facilitating efficient drug delivery [507]. Inhalable liposomal formulations of antibiotics [508], antifungals [509], and antifibrotics [510, 511] have been evaluated and have showcased the remarkable potential of liposomes as candidates in pulmonary delivery.

Designed to deliver chemotherapeutics to the lungs, researchers developed an Osimertinib (OSI)-loaded liposome with desirable aerosolization, with an MMAD of 4 μm and a fine particle fraction (FPF; defined as the percentage of an inhaled dose that consists of tiny particles small enough to reach deep into the lungs [512]) of 82%. In this study, researchers also observed that compared to passive drug loading, active (remote) drug loading of the liposomes not only enhanced EE, but also instigated sustained drug release, as actively-loaded liposomes releasing only 25% of OSI in the first 2 h, and then exhibited slow release over 72 h, whilst passively-loaded liposomes abruptly released 80% of the cargo in the first 2 h [513]. This slow and sustained release can protect healthy lung tissue from chemotherapy-associated toxicity.

One strategy to fabricate appropriate vehicles for pulmonary delivery is through dry powdering. Unlike sprays and aerosols, dry powder formulations offer improved stability, ease of use, and dosage metering [514]. Liposomes are one of the NDDSs capable of being dry powdered [515]. In the study by Patel et al., a liposomal dry powder of sorafenib tosylate was fabricated through the spray-drying method. The assessment of these fabricated particles showcased an MMAD of 3.15 ± 0.2 μm, an FPF of 83.7 ± 0.09%, and a dispersion of 85 ± 0.1%, indicating desirable properties to be applied as an inhalation-based carrier [516]. It has been observed that although dry powder preparation of liposomal formulations can alter pharmacodynamic and pharmacokinetic profiles, such as particle size, PDI, and active targeting, they showed comparable antitumor activity compared to the original liposomal formulation, and remarkable tumor tissue accumulation and lower normal tissue accumulation compared to IV administration [517].

Surface functionalization has also been employed to enhance drug accumulation and cell uptake following pulmonary administration. Designed inhalable anti-EGFR antibody (CTX)-conjugated OSI-loaded immunoliposomes demonstrated boosted drug delivery, efficacy, and cytotoxicity against H1975 cells, as well as remarkable mitigation of tumor cell migration and colonization, all of which were assumed to be attributed to EGFR targeting [518]. A folate-modified liposomal formulation encapsulating rapamycin was tailored and demonstrated high lung retention, accompanied by substantial antitumor activity and survival benefits [519], which were attributed to active targeting using folate moieties. Innovatively, inhalable spray-freeze-dried particles containing hyaluronic acid (HA)-coated siRNA-loaded liposome-protamine-DNA complexes demonstrated decreased toxicity toward normal human cells, followed by significant gene silencing in A549 lung cancer cells [520].

PhCs were also incorporated inside liposomes and applied via inhalatory delivery. CUR liposomal dry powder was formulated to enhance CUR delivery. The MMAD and FPF of the aforementioned particles were 5.817 ± 0.06 μm and 46.71 ± 5.23%, respectively, and despite successful deployment, there remains room for improvement in FPF and MMAD values. The formulation demonstrated remarkable cytotoxicity against lung cancer cell lines, whilst showcasing negligible toxicity against normal cells (BEAS-2B bronchial cells). Intratracheal administration of the liposomal formulation in an animal model of lung cancer outperformed free CUR and GEM, both in antitumor and anti-inflammatory effects [521]. Elsewhere, researchers employed proliposomes for pulmonary delivery of CUR. Proliposomes are the dehydrated liposome precursors that facilitate long-term storage and stability, and upon hydration, they form liposomes [522]. CUR proliposomes were fabricated and incorporated into a dry powder. The formulation achieved an MMAD of 2.10–3.18 μm and an FPF of 29.33–54.35%, both of which were better than those of unformulated CUR powder. Lung pharmacokinetics revealed faster uptake of the formulation, which was attributed to the lower MMAD of the fabricated NDDS [523].

**Fig. 12:**
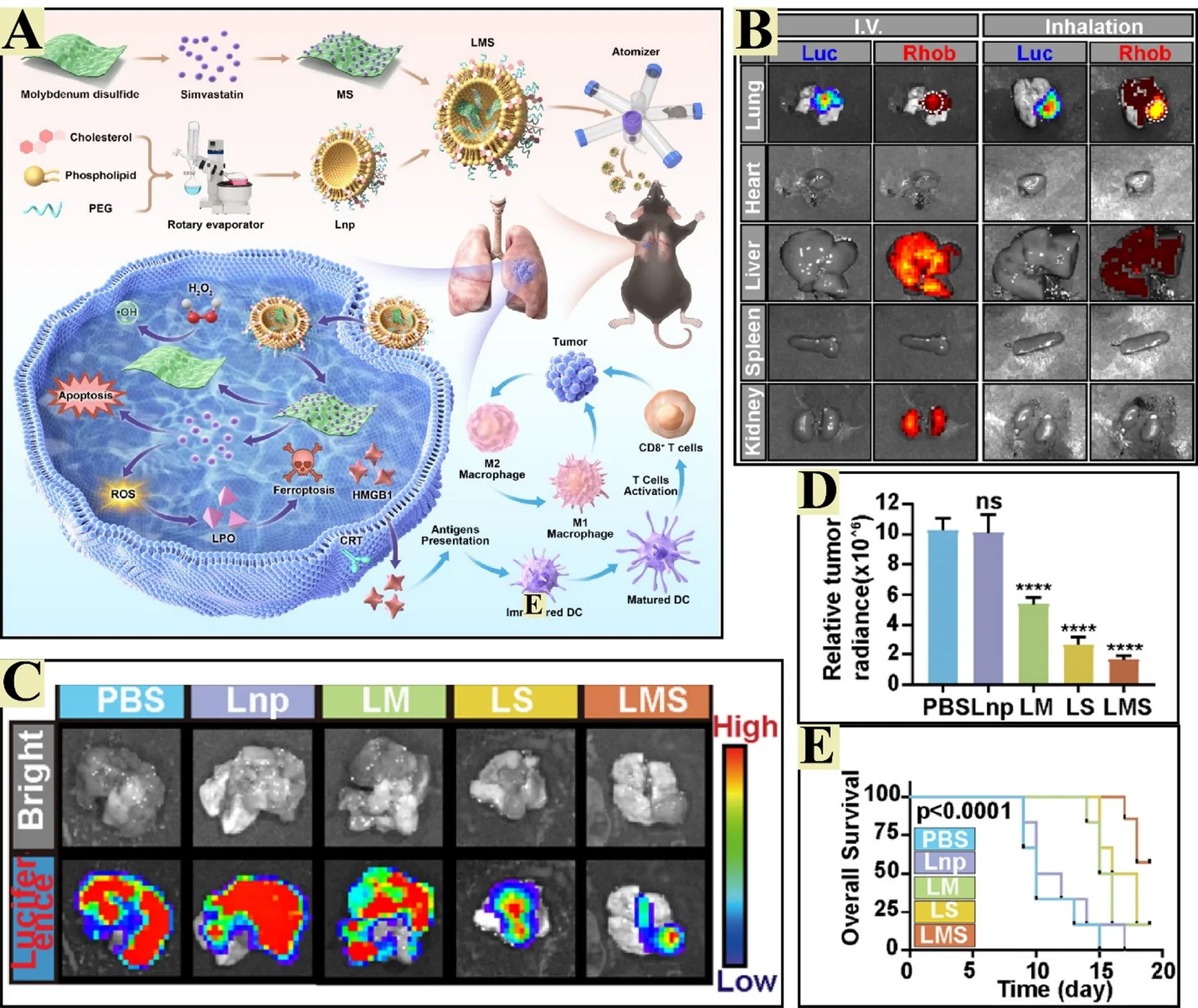
Inhalable liposomal platform for lung cancer immunotherapy. **A** A schematic view illustrating the fabrication of SIM-loaded MoS₂ nanosheets and subsequent encapsulation in a liposome. The final NDDS (LMS) was administered via the pulmonary route in orthotopic tumor-bearing mice, and following cellular internalization, it instigated ROS production and LPO, thus inducing apoptosis and ferroptosis, leading to DAMPs release from injured cells and provoking the immune response. **B** Comparison between distributions of LMS in IV vs. inhalatory administration. In inhalatory administration, off-target organ distribution of LMS was attenuated, whilst lung accumulation was heightened. **C** Ex-vivo bioluminescence images and (**D**) signal intensity chart of tumor-bearing lungs excised after treatment. **E** Survival curves of mice from different treatment groups. Reprinted with permission from reference [524]. Copyright © 2025 The Authors. Distributed under the Creative Commons Attribution-NonCommercial 4.0 International License (CC BY-NC 4.0).

Innovatively, with the aim of circumventing the immunosuppressive TME through reinforcing ferroptosis, a multifunctional NDDS was fabricated for pulmonary delivery. First, a SIM-loaded MoS₂ nanosheet was developed and encapsulated inside a liposome to improve drug delivery. SIM-mediated ROS production can promote lipid peroxidation (LPO) and incite ferroptosis, which ultimately calls forth DAMPs release and DC maturation, induces T-cell responses and overcomes immunosuppression. Moreover, the MoS₂ nanosheet can escalate ROS levels, thus aggravating oxidative stress. Pulmonary-administered liposomal formulation remarkably repressed tumor growth and extended OS (Fig. 12) [524].

**Fig. 13:**
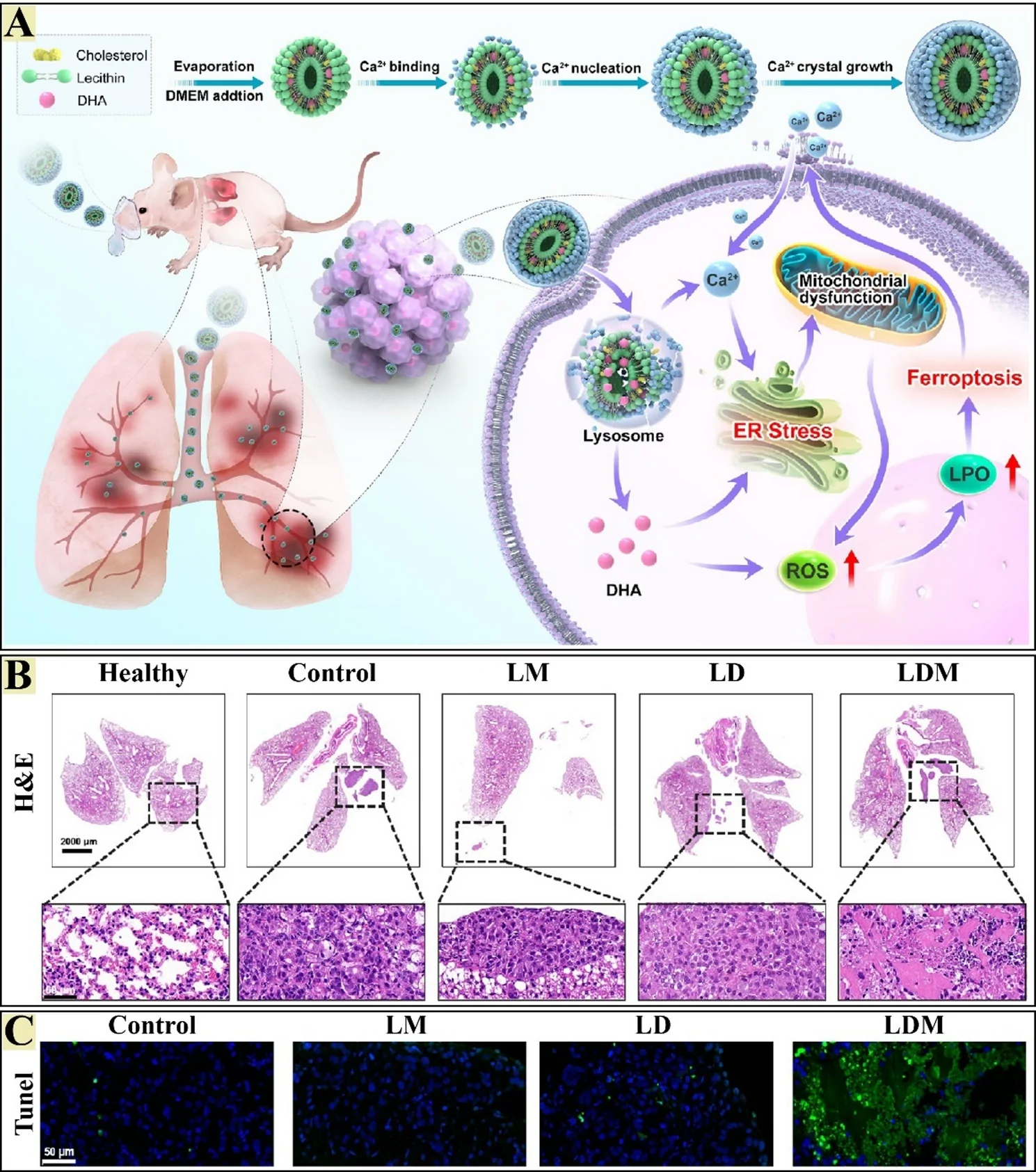
**A** A schematic illustration depicting the preparation of DHA-loaded liposomes that were surface-ornamented with calcium phosphate. When administered pulmonarily in an orthotopic lung cancer animal model, the liposomal formulation could accumulate in the lungs, and following cellular uptake, through Ca^2+^ and DHA-mediated increases in ROS and LPO, enhanced cellular ferroptosis and mitochondrial dysfunction, resulting in cancer cell death. **B** Histopathological evaluation of H&E-stained lungs demonstrates tumor regression in the LDM group, compared to other treatment groups. **C** TUNEL staining demonstrates the highest rate of apoptosis in the LDM group. LM, blank biomineralized liposome; LD, DHA-loaded liposome; Reprinted with permission from reference [525]. Copyright 2023 American Chemical Society

Dihydroartemisinin (DHA) is a derivative of artemisinin, which is isolated from *Artemisia annua*, and is known for its potent anticancer activity. Fu et al. fabricated an inhalable biomineralized liposome (LDM) for the co-delivery of DHA and surface-crystallized calcium phosphate to potently exert ferroptosis-mediated lung cancer therapy. The formulation showcased remarkable lung accumulation compared to the IV route, and demonstrated striking antitumor activity through reinforced ferroptosis induced by DHA and Ca^2+^ (Fig. 13) [525].

Several key factors can influence pulmonary drug delivery. Factors such as phospholipid composition and cholesterol concentration were correlated with higher quality of spray-dried powdered liposomes [526]. Liposomal composition also alters fluidity, which can impact the intrapulmonary behavior of liposomes, as lower fluidity was shown to reinforce mucus penetration, while higher fluidity can attenuate macrophage uptake [527]. Another contributing factor is the hydrophilicity/hydrophobicity balance, which affects pulmonary pharmacokinetics, as higher hydrophilicity is correlated with extended lung retention of inhaled liposomes [528]. As another factor affecting this, the role of surface charge remains controversial and requires further investigation [529, 530]. Therefore, it is imperative to meticulously equilibrate the contributing factors and to pursue more in-depth and innovative investigations.

**Fig. 14:**
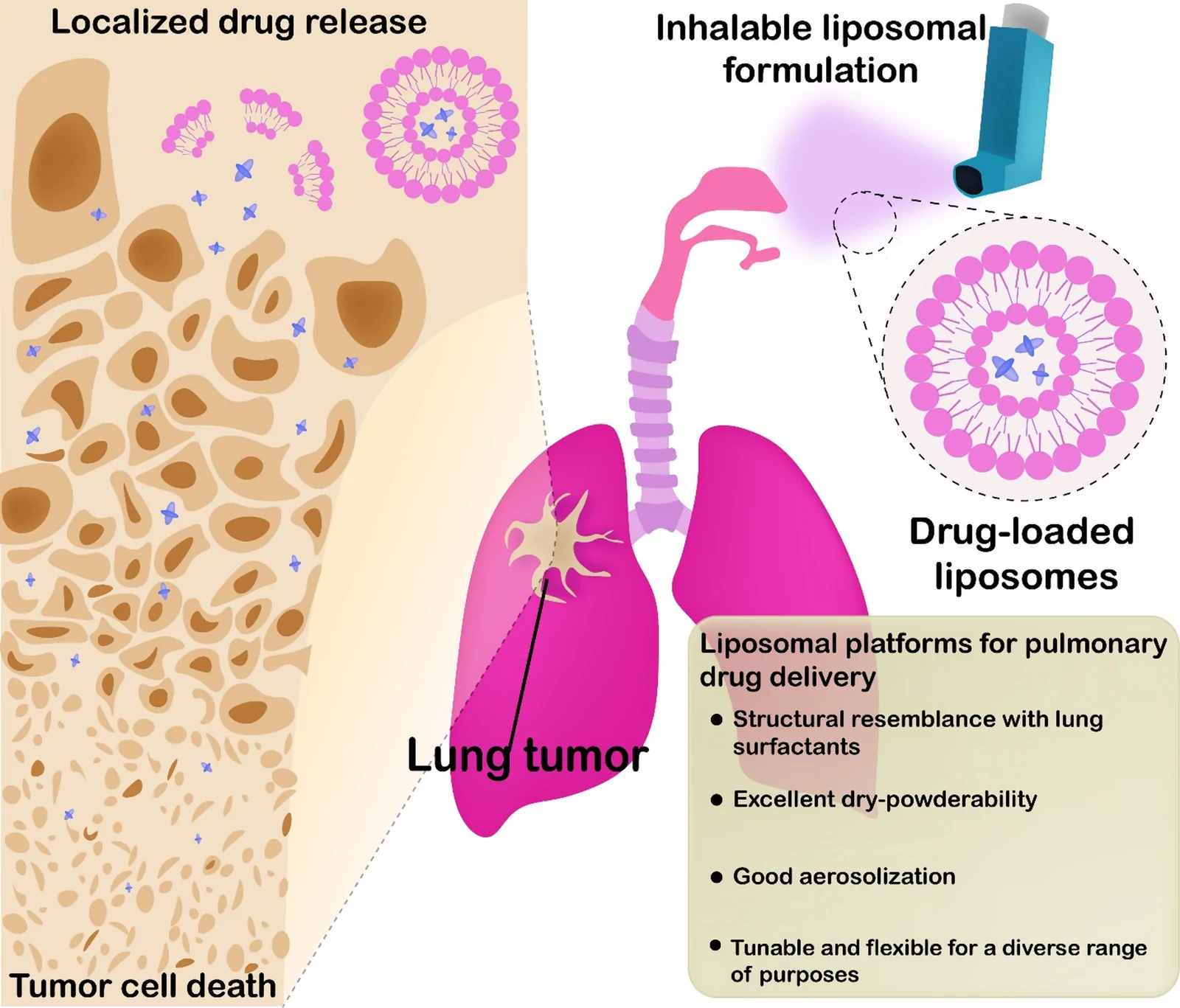
Localized pulmonary delivery harnessing liposomes in combating lung cancer

It is noteworthy that, although there are a handful of merits to the pulmonary delivery of NPs, including liposomes (Fig. 14) [531], there are also several challenges in inhalation-based drug delivery: (i) Mucociliary clearance: NPs can be entrapped in respiratory tract mucus and transferred outside the lungs; (ii) Alveolar macrophage uptake: clears particles in alveoli, preferably those sized between 500 and 5000 nm [532]; (iii) Inhalation based drug delivery can be accompanied with the risk of lung toxicity due to delivery of high concentration of drugs [533], and this toxicity can be heightened if the pulmonary-administered NPs deposit unevenly; (iv) Patient comorbidities, non-adherence or mal-usage of inhalation devices can impact the treatment outcomes [532, 534, 535]; (v) Safety concerns regarding the sterility of the nanomaterial fabrication process, as these materials are difficult and costly to be sterilized; (vi) Cellular and animal experimental models might not be able to completely reflect drug potency, and the heterogeneous and uneven nature of lung cancer may interfere with the interpretation of the efficacy of nanocarriers in lung cancer treatment [536].

We encourage researchers to broaden the scope of their studies and evaluate the ability of liposomes in overcoming the aforementioned challenges. Meanwhile, by harnessing and combining leading-edge drug delivery techniques such as active targeting with mucopenetrating [537], chemotaxis-mediated [538], target-triggered [539], and controlled-release strategies [540], researchers can advance the tumor accumulation of NDDSs, attenuate the pulmonary toxicity of chemotherapeutics, which acts as a major bottleneck in inhalation chemotherapy [541], and reinforce lung cancer inhalable oncotherapy.

## Forward-looking liposomal strategies for lung cancer

### Biomimetic and hybrid liposomes

#### Mechanistic rationale and biomimetic architecture

Several inherent shortcomings of conventional NDDSs, particularly liposomes, have motivated researchers to explore biomimetic approaches. Bare liposomes typically suffer from limited storage shelf-life and poor stability in biological fluids [542]. Although PEGylation has long been the gold standard surface coating, the induction of anti‑PEG antibodies and the ABC phenomenon have led the FDA to recommend screening for anti‑PEG antibodies in clinical trials, imposing an additional regulatory burden [543]. Additionally, PEGylation does not fully resolve the challenges associated with protein corona adsorption [544]. In this regard, coating liposomes with natural cell-derived membranes offers several advantages over conventional synthetic surface engineering strategies. In this section, we explain that these advantages can broadly be divided into two mechanistically distinct categories: general biomimetic properties conferred by membrane cloaking itself, and cell-specific functionalities inherited from the donor cell type [545].

Utilizing whole-cell membranes as coatings provides universal engineering advantages over traditional synthetic platforms. This strategy obviates the need for extensive sequential chemical ligand conjugation, enabling the simultaneous transfer of multiple bioactive motifs to the NP surface in a single fabrication step. This whole-membrane derivation generally preserves the baseline physicochemical characteristics and drug-loading capacity of the underlying liposomal core. Furthermore, the fluid and biologically derived nature of cellular membranes may improve interfacial compatibility within biological milieus relative to purely synthetic coatings [546].

Beyond these structural benefits, membrane-coated NPs inherit the donor cell’s complex glycocalyx, which grants unique cell-specific advantages in vivo. One of the earliest examples is the use of erythrocyte (RBC) membranes [547], which introduce high surface densities of CD47 “don’t eat me” signals that actively inhibit macrophage-mediated phagocytosis and prolong systemic circulation; subsequently, recent evidence supports that this prolonged circulation is also attributed to the membrane’s ability to actively suppress immune opsonin adsorption within the protein corona [548]. Macrophage-derived membranes also retain surface CD47 for immune evasion and display CCR2 and α₄β₁ integrins for chemotactic homing to tumor sites. By inheriting native adhesion molecules such as intercellular adhesion molecule-1 (ICAM-1) and vascular cell adhesion molecule-1 (VCAM-1), these biomimetic platforms actively cross endothelial barriers to accumulate in the TME [549], offering a versatile alternative to conventional polymer-based stealth coatings. Similarly, platelet-derived membrane coatings enable liposomes to target circulating tumor cells (CTCs) and actively inhibit their seeding into the premetastatic niche, a process mediated by the preservation of native platelet adhesion machinery via P-selectin/CD44 binding [550].

Cancer cell-derived membrane coatings enable homotypic targeting, defined as the preferential accumulation of nanocarriers in tumors of the same cellular origin. This phenomenon is commonly attributed to the function of native membrane components, including adhesion-related proteins such as integrins and cadherins, which mediate cell-cell and cell-matrix interactions [551]. In a lung cancer model, Jiang et al. demonstrated that A549 cell membrane-hybridized liposomes showed markedly higher uptake in homologous A549 cells, whereas uptake by normal alveolar epithelial cells was minimal [552]. However, the specific molecular determinants of NP homing in lung cancer models remain incompletely defined and require further investigation.

Translating these biomimetic concepts into practical designs requires careful consideration of NP architecture. While the interfacial coating of solid NPs (e.g., PLGA, silica, or gold) with cell membranes typically yields distinct core-shell architectures, the structural outcome when using liposomes may differ. Whereas some studies report concentric core-shell encapsulation, others demonstrate that membrane fusion predominates due to lipid bilayer homology, ultimately generating chimeric hybrid vesicles [553]. When employing solid cores, the fabrication process, typically driven by mechanical co-extrusion, sonication, or microfluidic electroporation, relies on the physical disruption and subsequent self-assembly of the isolated cell membrane vesicles (CMVs) around the rigid template [554, 555]. This results in a distinct core-shell architecture, where the solid NP dictates the final size, shape, and mechanical rigidity, while the biological membrane physically wraps the surface, stabilized by noncovalent forces [556]. However, in a recent review, Li et al. discuss how using fluid liposomes as a substrate yields a fundamentally different biomimetic architecture than the classic core-shell model [557]. Fabrication requires external energy, via co-extrusion shear, sonication, or freeze-thaw cycling, to transiently fragment the bilayers. Because both synthetic liposomes and natural cell-derived vesicle membranes consist of fluid phospholipid bilayers, these disrupted fragments can spontaneously reassemble and hybridize into hybrid vesicles [558]. The lipid composition of these hybrid vesicles directly dictates their functional behavior; incorporating cone-shaped helper lipids like DOPE introduces curvature stress that facilitates rapid membrane fusion and direct cytosolic cargo delivery by transiently altering the local lipid packing and fluidity of the target biological membrane [559]. Nevertheless, the literature contains examples of both membrane-coated and membrane-fused liposomes [560, 561]. Additionally, within this fusion model, structural heterogeneity may arise, meaning that not every vesicle is perfectly hybridized with an identical composition [558]. This poses limitations for batch-to-batch reproducibility, presenting a major obstacle to the clinical translation of biomimetic liposomes [557].

#### Therapeutic applications and translational considerations in lung cancer

**Fig. 15:**
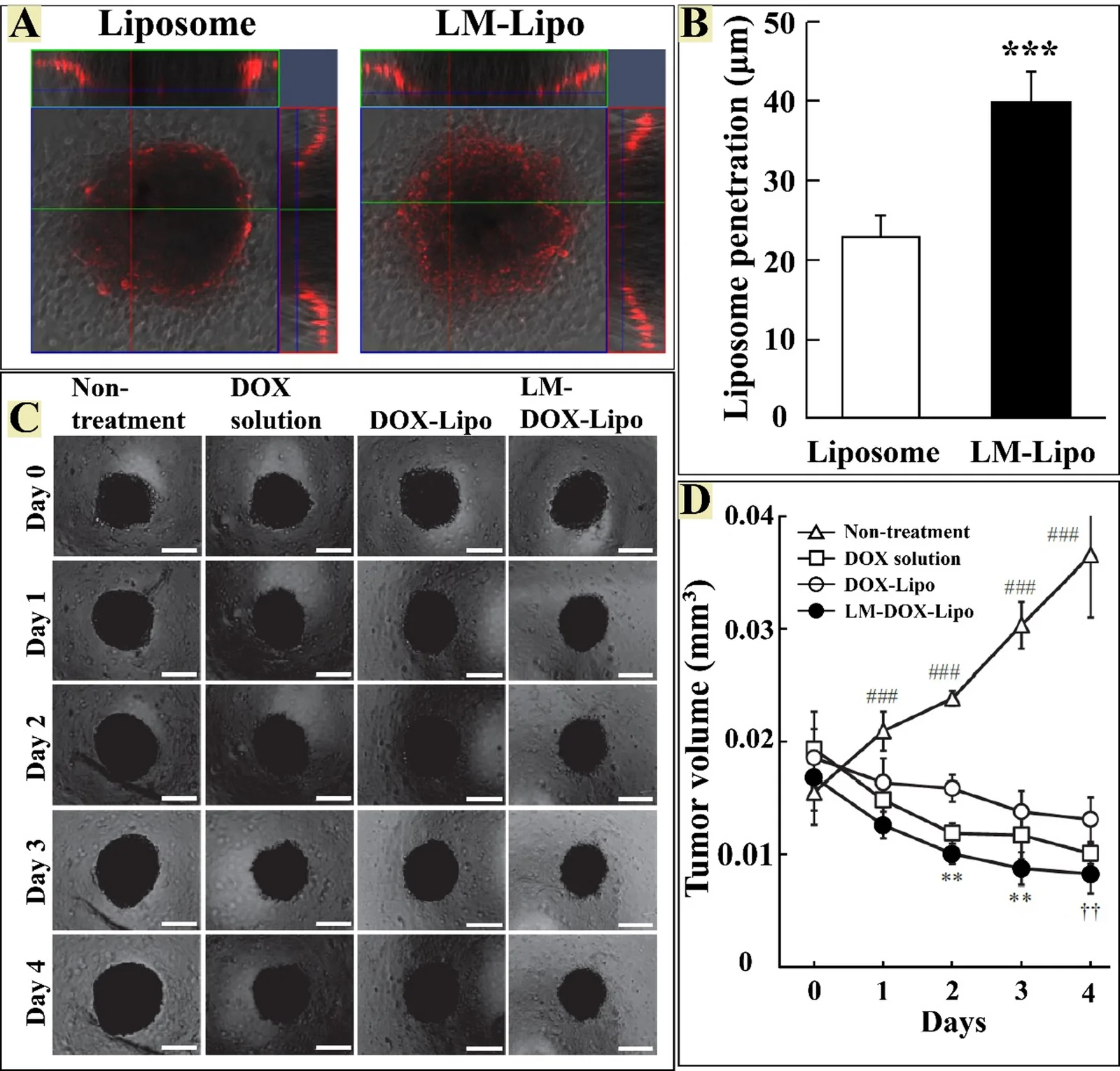
Leukocyte-mimetic liposomes (LM-Lipo) are designed for enhanced tumor penetration. **A** Microscopic images and (**B**) quantitative analysis of the spheroid A549 cell model, showcasing that LM-Lipo outperformed plain liposomes in penetrating the tumor spheroid. **C** & **D** Evaluation of the anti-cancer effect of different treatments indicated a superior anti-tumor effect of LM-DOX-Lipo. Reprinted with permission from ref [562]. Copyright © 2020 Elsevier.

Various biomimetic platforms have been specifically tailored for pulmonary oncology. To harness the ability of leukocytes to penetrate tumor tissue, a leukocyte-mimetic liposome containing leukocyte membrane proteins was fabricated and loaded with DOX (LM-Lipo). In an A549 spheroid cell model, LM-Lipo could favorably penetrate into the tumor spheroid and repress its growth compared to simple liposomes (Fig. 15) [562].

Comparative head-to-head analyses of cell membrane origin remain sparse within the biomimetic literature. Addressing this, Zhou et al. systematically evaluated macrophage membranes derived from M0, M1, and M2 phenotypes as functional liposomal coatings for lung cancer targeting. Despite distinct inflammatory profiles, all three variants showed comparable Siglec-10 surface expression and similar levels of tumor cell internalization, suggesting that macrophage polarization state exerts limited influence on targeting efficiency mediated by the Siglec-10/CD24 axis [563]. These findings highlight the need for more head-to-head comparisons of membrane sources, which remain limited but are essential for guiding rational design and informing future clinical translation.

Furthermore, recent advances in biomimetics have increasingly explored the hybridization of liposomes with multiple cell membrane types, aiming to integrate complementary functionalities from different donor cells within a single nanoplatform [564]. For instance, Liu et al. developed a hybrid erythrocyte and LLC membrane-coated liposomal platform for the co-delivery of CUR and astragaloside IV. This dual-membrane configuration combined the prolonged systemic circulation associated with erythrocyte cloaking with the homologous targeting capability of LLC membranes, resulting in increased tumor accumulation and stronger suppression of tumor growth in LLC-bearing mice in vivo [565]. However, the absence of single-membrane controls limits mechanistic interpretation of the individual contributions from the erythrocyte and LLC membranes, leaving it unclear whether the combined effect arises from simple additive contributions of each membrane or from emergent synergistic interactions between them.

Chimeric antigen receptor T (CAR-T) cells are engineered T cells that express desired CARs on their surfaces, which can act like antibodies to detect and attach to cancer antigens [566]. In a forward-looking study, exosomes, a type of extracellular vesicles containing the cell’s surface and intracellular components (e.g., proteins and nucleic acids) [567], derived from an engineered bispecific CAR-T cell, were fused with lung-targeting liposomes and loaded with PTX to benefit from cell-targeting, lung-targeting, chemotherapy, and the CAR-T exosomes’ cytotoxic molecules (such as perforin) all at once. The ultimate nanocomposite accumulated in the lungs and, through the presence of the surface CAR (anti-mesothelin), targeted the cancer cells and delivered PTX and cytotoxic molecules, while the other CAR (anti-PDL1) blocked PD-L1 to prevent T-cell immunosuppression [568]. Fusion with liposomes effectively overcame the low drug-loading limitation of exosomes.

Utilization of tumor-derived nanovesicles (TNVs) as coatings or hybrid components of NDDSs has garnered tremendous momentum in recent years, as they can facilitate tumor cell targeting and uptake of these engineered nanoplatforms [569]. To enhance the insufficient drug loading of TNVs, Asadujjaman et al. fused A549-derived TNVs with DTX-loaded liposomes to enhance tumor cell targeting and uptake of the hybrid NPs. The fabricated NPs showcased enhanced A549 internalization (3-fold), reinforced cytotoxicity (5-fold lower IC_50_), and attenuated immune cell clearance (1.5-fold) when compared to liposomes. Furthermore, the hybrid NPs demonstrated a stronger in vivo antitumor effect compared to liposomes [570]. Integration of such systems, which can compensate for the shortcomings of one another, may be the key to unlocking the ever-challenging gates of oncotherapy.

Finally, pathogen-inspired biomimetics have been developed to enhance intracellular delivery. In an innovative study designed to cope with low intracellular accumulation, a virus-mimicking liposome was fabricated by the surface affixation of comb-shaped pseudopeptides, similar to fusogenic peptides in virus spikes, on cholesterol-integrated liposomes that are akin to a viral envelope. In vitro evaluation in A549 cells showcased that the formulation could be effectively internalized and exert robust cytotoxicity [571].

Despite fascinating outcomes in biomimetic research, the clinical translation remains constrained by universal bottlenecks, including variable membrane fusion efficiency compromising reproducibility, stringent quality control requirements, and high manufacturing costs. Furthermore, depending on the membrane source, severe immunogenicity and safety risks can emerge, driven by the presence of foreign major histocompatibility complex (MHC) molecules on allogeneic nucleated cells when utilizing immortalized donor cell lines [572]. Hybrid cell membrane coatings may also introduce additional sources of variability compared with single-cell systems, as differences in donor cell type, membrane composition, and the relative proportions of fused membranes could alter vesicle structure, surface protein presentation, and functional behavior, potentially complicating clinical translation.

### Stimuli-responsive liposomal systems

The term “smart nanoplatforms” has been used in recent years to describe avant-garde NDDSs that are capable of responding to intrinsic stimuli/environment or an extrinsic impulse to release the cargo or to initiate a reaction (e.g., bubble formation [573]) [574]. In this stimuli-responsive behavior, the drug release or reaction can be triggered by various factors such as environmental acidity (pH-responsive), changes in O₂ (hypoxia-responsive), and temperature changes (thermo-responsive), which are all consistent with tumor tissue, as it exhibits low pH, hypoxia, and is usually hotter than normal tissues [574, 575]. The stimuli-responsive behavior can result in enhanced therapeutic efficacy via controlled and site-specific drug release, alongside attenuating systemic toxicities [576–579].

As a well-established NDDS, liposomes have also been examined to act as multifunctional and smart platforms. Regarding pH-sensitive liposomes, in vitro evaluation of afatinib (AFT)-loaded pH-responsive liposomes has showcased robust AFT release at acidic pH and remarkable cytotoxicity against the H-1975 lung cancer cell line [580]. Demirbolat et al. fabricated two different cholesteryl hemisuccinate (CHEMS)-integrated pH-sensitive liposomal Cis formulations and compared the EE, pH-mediated drug release, and cytotoxicity. Although DOPE-incorporated liposomes achieved lower EE compared to phosphatidylcholine-incorporated liposomes, they exhibited robust Cis release in an acidic milieu (pH = 5.5). Moreover, despite the comparable cytotoxicity of the two liposomal formulations, DOPE-incorporated liposomes showcased stronger repression of cell-to-cell intercommunication [581]. DOTAP-integrated liposomal ATRA demonstrated favorable pH-responsive behavior at pH = 6 and exceptional cytotoxicity toward A549 cells when compared with the free drug [582]. Although these studies lack in vivo assessment of the nano-liposomes, they accentuate the importance of the structural composition of NDDSs in their therapeutic behavior.

In more sophisticated research, Park et al. fabricated a dual-loaded doxycycline─ a calpain-2 (CAPN2) inhibitor, which contributes to NSCLC growth─ and DTX pH-sensitive liposome and functionalized this NDDS with folate to target FRβ. The release of the drugs was pH-dependent, as it exhibited boosted release at pH = 4 compared to pH = 6.5. In an A549 animal model, application of the liposomal formulation inhibited tumor growth, as the treatment group achieved the lowest tumor size, highest anti-angiogenic capability, and diminished M2 macrophage count, which was assumed to be correlated with active targeting of tumor cells and TAMs, pH-responsiveness, and synergistic anti-neoplastic co-therapy [583].

**Fig. 16:**
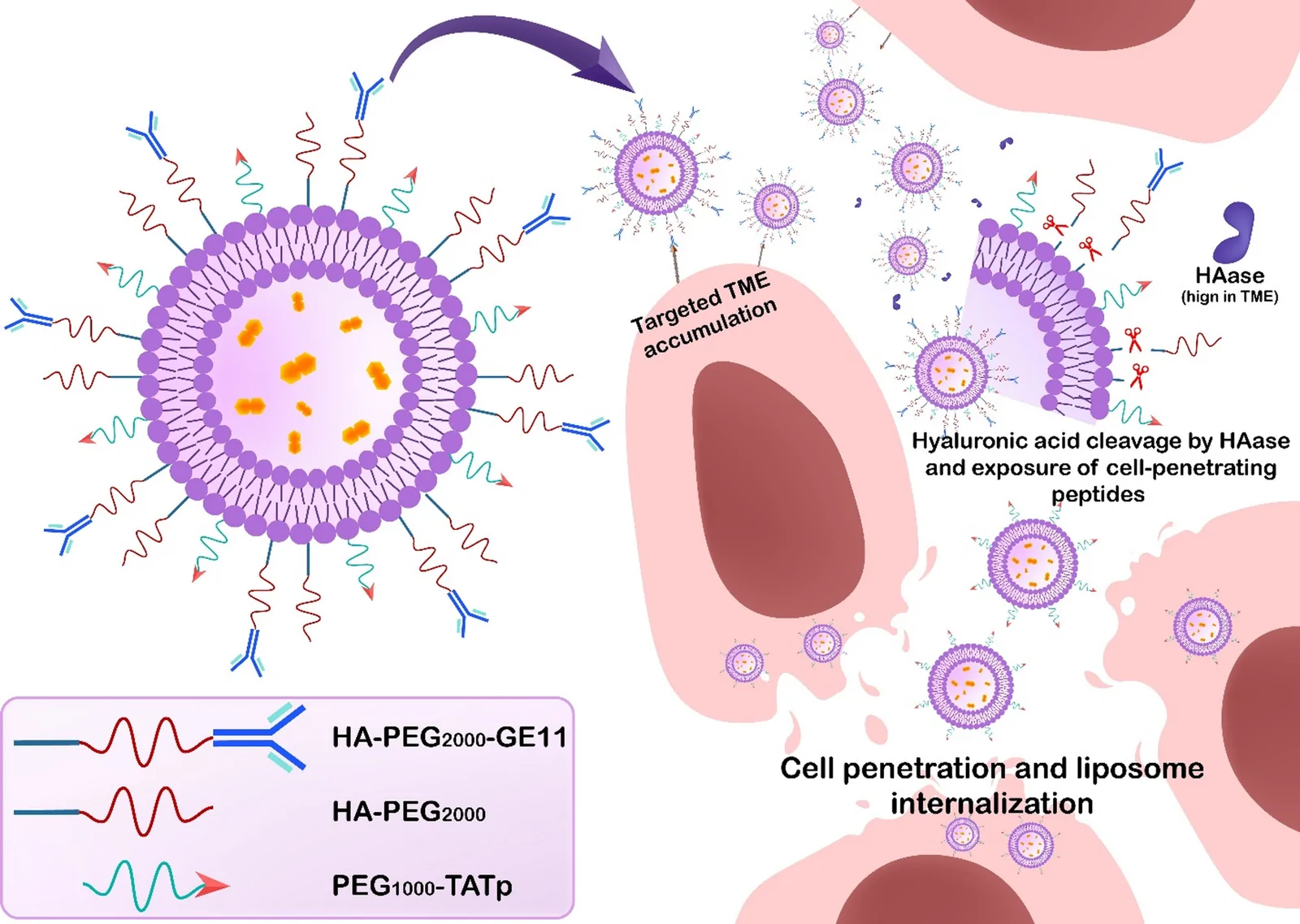
A schematic illustration of a TME-sensitive liposomal platform for delivery of irinotecan (IRI). Based on overabundant HAase in TME, a triple-modified liposome was designed for smart and TME-targeted cell penetration and cellular entrance. The HA was used as a linker for surface-affixing PEG_2000_-GE11, which actively targets over-expressed EGFR on lung cancer cells, and PEG_2000_. HA-PEG_2000_-GE11 and PEG_2000_ concealed PEG_1000_-TATp ligands, which were shorter than the other two ligands, to prevent unwanted and off-target effects. When reaching TME with the help of active targeting, the overexpressed HAase in TME can cleave the linker HA, uncovering the cloaked PEG_1000_-TATp ligands, facilitating cell penetration and liposomal entrance. Adapted with permission from ref [587]. Copyright © 2026 Ivyspring International Publisher. This is an open-access article distributed under a Creative Commons Attribution-Noncommercial 4.0 International license

The TME itself is another target for responsive NDDSs, which can react to imbalanced enzyme concentration, hypoxia, and acidity [584]. Harnessing the overabundance of MMP-2 in the TME, a responsive liposomal platform modified with an MMP-2-cleavable peptide-shielded CPP achieved higher cytotoxicity and cellular internalization in A549 in vitro and in vivo models [585]. A multifunctional MMP-9-stimulated, pH-sensitive, and redox-activated TME-responsive liposome and cancer cell membrane were hybridized and coated with the lipoic acid-modified polypeptide for gene delivery, and chemotherapy demonstrated potent oncotherapeutic potential in NSCLC [586]. Another study focused on the TME’s elevated hyaluronidase (HAase) and fabricated a triple-modified CPP (TATp), HA-PEG, and HA-PEG-EGFR antibody (GE11) liposome for targeted and responsive IRI delivery. This multifunctional liposome targeted the TME via GE11-mediated active targeting, and subsequent HA degradation following HAase activity in the TME resulted in the hidden TATp being exposed, thus increasing cell penetration and internalization (Fig. 16) [587].

Notwithstanding the encouraging results of smart liposomes in preclinical studies, a wide gap exists between the preclinical and clinical status of stimuli-responsive nanomaterials, as roughly 1% of preclinical investigations proceed to the clinical trial phase. Several challenges stand in the way of effective clinical translation of stimuli-responsive platforms that have yet to be addressed properly. As reviewed by Kobzev et al., (i) off-target drug release due to deficient selectivity toward intrinsic stimuli, (ii) intricate design procedures, (iii) high production costs, (iv) suboptimal tumor penetration of extrinsic stimuli, and (v) regulatory issues were regarded as main obstacles to the clinical translation of smart nanoplatforms [588]. We encourage researchers to keep the aforementioned challenges in mind when designing preclinical studies, alongside contemplating the potential of clinical translatability as a primary goal in line with the study’s other objectives.

### Theranostic liposomes

Cancer theranostics integrates diagnosis and therapy and offers a singular multifunctional platform for concomitant diagnosis and monitoring and oncotherapeutic purposes. Nanotheranostics are a powerhouse subdivision of the theranostic realm [589], with liposomes serving as an accentuated well-studied constituent.

Studies have chosen liposomes as NDDSs for lung cancer theranostics. Green-synthesized quantum dots (QDs) and CUR were co-loaded into liposomes, and the evaluation demonstrated that QDs were capable of emitting red fluorescent signals, which can be used for detection of cancerous cells. Moreover, the liposomal formulation enhanced the cellular uptake and cytotoxicity of CUR compared to free CUR [590]. In an artful study, Jha et al. fabricated DNA biodots, which are QDs fabricated from biomolecules, such as DNA and amino acids [591], and co-loaded them with etoposide into CTX-functionalized pH-responsive liposomes for theranostic application in an NSCLC model. Although this study lacked the in vivo antitumor evaluation, the targeted liposomal formulation achieved the highest cellular accumulation (evident by yellow fluorescence of biodots) and robust cytotoxicity and apoptosis in vitro, showcasing the ability of this platform to both spot and kill cancer cells [592].

In spite of the attractiveness of simultaneous diagnosis and treatment enabled by nanotheranostics, these platforms are still at their infancy. The lack of adequate studies on theranostic liposomes for lung cancer itself underscores the considerable difficulty involved in designing and implementing dual-purpose nanotheranostic platforms. Obstacles like overcomplicated manufacturing, biosafety issues, and the shortcomings of preclinical models in reflecting complex human tumors have impeded the applicability of nanotheranostics [593]. Moreover, the dynamic TME and high degree of intratumoral heterogeneity [594] may undermine precision imaging, even in the presence of active targeting, as variable ligand expression of cancer cells can lead to uneven cellular uptake of nanotheranostics, resulting in imprecise imaging. It is noteworthy that researchers should decipher whether liposomes are suitable for lung cancer theranostics by conducting head-to-head comparison studies with other nanoplatforms.

## Patent landscape and technological trends

Literature search alone does not always capture the full picture of technological development in the field, and many formulation innovations are absent from the peer-reviewed literature [595]. While most researchers prioritize publishing their findings in scientific articles, patent analysis is still required to access important technological developments that are often protected or not disclosed in publications [596]. Patent analysis is therefore required to access these developments, providing insight into emerging real-world trends in DDSs for cancer treatment that are not fully represented in scientific publications. Based on a broad patent search in Espacenet, recurring patterns and several trends were identified and categorized into three main axes.

1. Reformulated chemotherapy: This developmental axis is dominated by strategies that encapsulate classical agents, such as PTX, IRI, and Cis, within liposomal systems with modified lipid composition or surface properties. These approaches rarely introduce new drugs; instead, they refine EE, circulation behavior, and targeting specificity. For example, US2022031618A1 describes an IRI liposome employing sulfonate gradients (e.g., ammonium ethanedisulfonate) that achieves sustained release (1.03% at 24 h) compared with conventional ammonium sulfate (15.34% at 24 h) [597]. However, although lung cancer applications are claimed, no in vivo lung-specific data are provided, reflecting a common gap between broad claims and disease-specific validation. Furthermore, patent claims are often intentionally broader than the supporting experimental evidence, reflecting strategic intellectual property positioning rather than validated therapeutic scope. Despite these refinements, preparation methods remain highly conserved, repeatedly relying on thin-film hydration, extrusion, and remote loading techniques. Innovation therefore is concentrated in compositional adjustments and functional additions rather than in synthetic methodology. This convergence suggests that the field has reached a relatively mature stage, where manufacturing feasibility constrains the design space.
2. Active targeting ligands: The choice of active targeting ligands represents an additional layer of design complexity. A clear trend across recent patents is the increasing use of peptide- and antibody-based ligands, in contrast to earlier reliance on small molecules such as folic acid, as reflected in receptor-targeted liposomal systems including ApoPep-1 and SP5-2 peptides [598, 599]. Furthermore, conjugated antibodies serve as targeting moieties to enhance tumor accumulation, rather than as immunomodulatory agents designed to activate or redirect immune responses. This shift toward peptide- and antibody-conjugated liposomes reflects both accumulated evidence on receptor specificity and growing recognition of off-target uptake mediated by ubiquitously expressed transport systems. For example, US2016228573A1 describes an antibody-conjugated liposomal system targeting folate receptor α while explicitly avoiding the use of folic acid-based ligands to reduce non-specific interactions with physiological transport systems in healthy tissues [600]. More recent filings extend toward dual- and multi-targeted liposomes, combining multiple ligands or integrating targeting with additional functionalities to address tumor heterogeneity and MDR [601, 602]. While these designs offer increased conceptual precision, they also introduce greater formulation complexity, and their translational advantage over simpler systems remains uncertain. In addition, increasing structural complexity in multi-component systems may impose manufacturing and regulatory constraints that limit clinical translation despite their conceptual advantages.
3. Payload diversification and design variables: While earlier patents predominantly describe liposomal formulations of conventional cytotoxic agents, diversification of encapsulated payloads is evident. A subset of patents incorporates biologically complex cargos, including nucleic acids and immunomodulatory agents. Gene delivery approaches, such as liposomes carrying siRNA or plasmid DNA targeting oncogenic pathways, are described, but remain limited in number [599]. Immunotherapy-related patents in this field are predominantly centered on cancer vaccines, such as the well-known BLP25 (tecemotide) liposomal vaccine [603]. Non-vaccine immunomodulatory approaches remain relatively sparse; for example, AU2026201779A1 describes liposomal delivery of an IDO inhibitor prodrug intended to modulate the tumor immune microenvironment [604]. Phytochemical-based payloads are also represented across multiple patents, frequently rationalized by improved stability and therapeutic performance. WO2019105408A1 demonstrates that incorporation of ginsenoside Rg5H into the lipid bilayer enhances antiproliferative activity and reverses PTX resistance in A549/Taxol cells, reducing the IC₅₀ of PTX to 0.72 µM compared to 2.70 µM with ginsenoside Rp1-containing liposomes [605]. Similarly, WO2024222862A1 reports freeze-dried tangeretin liposomes with sustained release and improved cytotoxicity in resistant models [606]. Notably, several patents involving liposomal PhCs in this dataset carry Chinese patent numbers (CN series), suggesting active contributions from Chinese research groups and a possible ethnopharmacological orientation (see Table 4). Diversification is further observed in phototherapy-oriented formulations, although these remain comparatively sparse. For instance, CN110179754A describes a redox-responsive liposome co-encapsulating the photosensitizer Ce6 and a Cis prodrug, with reported tumor growth inhibition following near-infrared irradiation [607].

In addition to payload and targeting modifications, a consistent feature across the analyzed patents is lipid and membrane-level engineering, including variation in lipid composition, incorporation of functional lipids, and modification of bilayer properties. These changes are not incidental but represent a central design variable, indicating that the lipid bilayer is increasingly treated as an active and tunable component rather than a passive carrier (Table 4). One design dimension emerging from the patent landscape is route-of-delivery optimization, particularly through inhalable liposomal systems. Within the extracted dataset, patents addressing aerosolized or pulmonary delivery remain relatively limited but are more frequently represented in recent filings. Several describe nebulized or inhaled formulations designed to achieve direct pulmonary deposition. An observed design feature in a subset of these patents is the use of pulmonary surfactant-mimetic lipid systems to enhance alveolar deposition and retention within the lung microenvironment [608, 609]. For example, WO2019221544A1 reports a PTX-loaded formulation derived from natural lung surfactant, which, after nebulization, produced an approximately 30% reduction in lung tumor area based on histopathological assessment; however, no standardized quantitative tumor burden metrics were provided [608]. In another example, US2023092682A1 employs a natural pulmonary surfactant system combined with a protamine linker to facilitate binding of negatively charged proteins or nucleic acids. In vivo, inhaled particles demonstrated predominant retention within lung tissue for up to 24 h with widespread alveolar distribution [609]. This system leverages electrostatic interactions within a surfactant-based matrix via a protamine bridging component to enable incorporation of negatively charged proteins or nucleic acids. These inhalable systems are often built upon otherwise conventional liposomal platforms, suggesting that innovation in this axis is driven primarily by reconfiguration of delivery route rather than fundamental redesign of the carrier architecture. This pattern also highlights a potential translational hurdle, particularly the variability and regulatory complexity associated with natural or biologically derived surfactant materials.

**Table 4:** Summary of liposomal delivery systems for lung cancer therapy from patent literature

No.	Patent No.	Year	Payload	Formulation / Design Features	Key Outcome / Claim	Ref.
1	CN103417551A	2013	Paclitaxel + Bufalin	Liposome (lecithin, cholesterol, DSPE-PEG2000); co-delivery	~ 68% tumor inhibition in A549/NCI-H446 xenograft; reduced toxicity	[610]
2	WO2019105408A1	2019	Paclitaxel + Ginsenoside derivative (Rg5H, etc.)	Liposome with ginsenoside derivative as active membrane material; phospholipid (egg lecithin, HSPC, DPPC, etc.) ± cholesterol	Superior to free paclitaxel in A549/Taxol resistant xenograft; reversed paclitaxel resistance	[605]
3	WO2025185024A1	2024	Docetaxel	Monomolecular phospholipid membrane (DOPC) with neutral lipid hydrophobic core (fish oil/tricaprylin); surface functionalized with LTA-P33 (lung), ApoE (liver), or BCMA antibody (hematologic tumors)	Lung-targeted docetaxel adiposome achieved 79.8% tumor inhibition vs. 45.3% for docetaxel injection; superior safety with lower hemolysis and reduced weight loss	[611]
4	US2016067322A1	2016	BLP25 lipopeptide (MUC1 antigen) + MPL adjuvant	Liposomal vaccine formulation; MUC1-targeting lipopeptide with monophosphoryl lipid A (MPL) as immunostimulant	Phase III START trial did not meet its primary overall survival endpoint (hazard ratio [HR] 0.88, *p* = 0.123); subgroup analysis of concurrent chemoradiotherapy patients showed exploratory HR of 0.78	[603]
5	WO2019221544A1	2019	Paclitaxel	Natural pulmonary surfactant-derived liposome; contains membrane proteins (SP-A, SP-B, SP-C, SP-D)	Inhaled formulation achieved ~ 30% tumor area reduction in A549 xenograft; superior cellular uptake vs. synthetic mimic liposomes; 24 h lung retention; low toxicity	[608]
6	CN102008438A	2010	Cisplatin	Stealth liposomes surface-modified with anti-lung cancer monoclonal antibody F(ab’)2 fragments	Active targeting achieved 209.57% higher lung distribution and 80.34% higher tumor inhibition vs. free cisplatin; reduced off-target organ distribution	[612]
7	CN105726483A	2016	Sunitinib + diterpenoid derivative	RGD peptide-conjugated liposomes; hydrogenated phospholipid + cholesterol + cyclodextrin + DSPC-PEG2000 + nonionic surfactant	RGD-targeted liposomes achieved 92% inhibition in A549 cells (sunitinib + diterpenoid derivative vs. sunitinib alone)	[613]
8	CN104043133A	2014	T-VISA-BikDD plasmid (pro-apoptotic gene BikDD)	Cationic nanoliposomes (DC-CHOL: DOPE, 1:2 molar ratio)	T-VISA-BikDD liposomes induced significant apoptosis in H1975 and H1299 lung cancer cells vs. empty vector (*P* < 0.05), and inhibited tumor growth and prolonged survival in an in situ (lung-injected) H1299-luc nude mouse model	[614]
9	CN121221531A	2024	HDAC inhibitor (MS275 or SAHA)	Liposome (hydrogenated lecithin/cholesterol, 50:40) co-functionalized with DSPE-PEG-gefitinib (EGFR targeting) and DSPE-PEG-lenalidomide (E3 ligase recruitment); molar ratio 50:40:8:8:1	Showed strongest tumor inhibition in osimertinib-resistant H1975-OR xenograft vs. controls	[601]
10	CN117618348A	2023	Sorafenib + resveratrol	FA-targeted hybrid liposome (lecithin/cholesterol/PF127/DSPE-PEG2000-FA) + resveratrol-loaded PF127 micelles	Strongest tumor inhibition in A549 xenograft; IC₅₀ reduced 4.7× vs. free sorafenib; prolonged survival; no systemic toxicity	[615]
11	US11123353B2	2019	Ergosterol + gefitinib	SPC/Chol liposome (5:1) with RGD/R8 peptide modification	Targeted co-loaded liposomes showed greater tumor inhibition than free gefitinib in PC-9/GR xenograft	[616]
12	CN109876156A	2019	BIM-S plasmid	Folate-modified cationic liposome (DOTAP: Chol: mPEG-suc-Chol: F-PEG-suc-Chol = 50:45:4.75:0.25)	Reduced lung tumor growth and prolonged survival in LL/2 lung cancer-bearing mice; induced apoptosis in lung cancer cells and FRβ-positive M2 macrophages; reduced microvessel density	[617]
13	US2020164086A1	2019	None (immune cell labeling platform)	Fusogenic liposome (DOPC: DOTAP: DSPE-PEG2K: DOPE-PEG4-azide/BCN); surface-functionalized with clickable groups for antibody conjugation	System demonstrated labeling and immune-mediated killing of A549 human lung cancer cells	[618]
14	CN107432875A	2017	Hispidulin + epigallocatechin gallate	Folate-PEG-targeted liposome (lecithin/cholesterol/DSPE-PEG-FA)	In vitro: FA-targeted co-loaded liposome increased apoptosis in A549 cells to 42.0%, vs. 23.4% for free HPDL+EGCG combination; also active against A549 cancer stem cells (CD133⁺, ALDH1⁺); no in vivo data	[619]
Biomimetic/Hybrid Liposome						
15	CN118286157A	2024	IGF2BP3 siRNA plasmid + osimertinib	SP-B-expressing cell membrane coating; PABE/DNA NSs + osimertinib-loaded liposomes; inhalation delivery	Inhibited orthotopic lung tumor growth; prolonged survival (80% at 70 days)	[620]
16	WO2025100747A1	2023	Anticancer agents	Hybrid NP: stem cell-derived ectosomes (educated with cancer cell medium) fused with PEGylated liposomes (DOPC: cholesterol: PEG2000-PE, 60:35:5)	Ecto-liposome fusion NPs showed enhanced targeting to A549 lung tumors in mouse xenograft model; improved tumor cell uptake vs. liposomes or ectosomes alone	[621]

*MPL* Monophosphoryl lipid A, *Rg5H* Ginsenoside derivative (active membrane component), *LTA-P33* Lung-targeting peptide (Neuropilin-1 binding), F(ab′)₂, divalent antibody fragment lacking Fc region, *H1975-OR* Osimertinib-resistant H1975 lung cancer cell line, *FA* Folic acid, *PC-9/GR* Gefitinib-resistant PC-9 lung cancer cell line, *BIM-S* pro-apoptotic Bcl-2 family isoform, *mPEG-suc-Chol* methoxypolyethylene glycol-succinyl-cholesterol, *F-PEG-suc-Chol* Folate-polyethylene glycol-succinyl-cholesterol, *BCN* Bicyclo[6.1.0]non-4-yne (copper-free click chemistry handle), *IGF2BP3* Insulin-like growth factor 2 mRNA-binding protein 3.

A limitation of this patent analysis is potential language bias. Many patents, particularly those filed in Chinese, are available in Espacenet only through machine-generated English translations, which may result in loss of technical nuance or underestimation of non-English innovation. Additionally, many patents broadly claim applicability to lung cancer therapy, yet provide little or no lung cancer-specific experimental data, often relying on general cancer cell lines or subcutaneous xenograft models instead of orthotopic or lung-specific systems.

## Conclusion and future outlook

Lung cancer poses an immense burden, as it is the leading cause of cancer-related mortality. This necessitates the design of precise and efficient treatment approaches to overcome its high MDR and the limitations of current oncotherapy. Liposomal NDDSs represent a well-studied platform, are endowed with unique structural distinctiveness and tunability, and have been widely utilized as a potent carrier in lung cancer therapy, to augment chemo-, immuno-, and phototherapy efficacy, and enhance the delivery of these therapeutics. Liposomes have also been utilized to deliver phytochemical and nucleic acid payloads, reinforcing the efficacy of these treatment strategies in lung cancer therapy. The ability of liposomes to be administered via the inhalation route and to be formulated as dry powder positions them as promising candidates for pulmonary administration. Moreover, advanced stimuli-responsive, biomimetic, and theranostic liposomal platforms have introduced additional layers of multifunctionality, circumvented several biological barriers, and potentiated lung cancer oncotherapy.

**Fig. 17:**
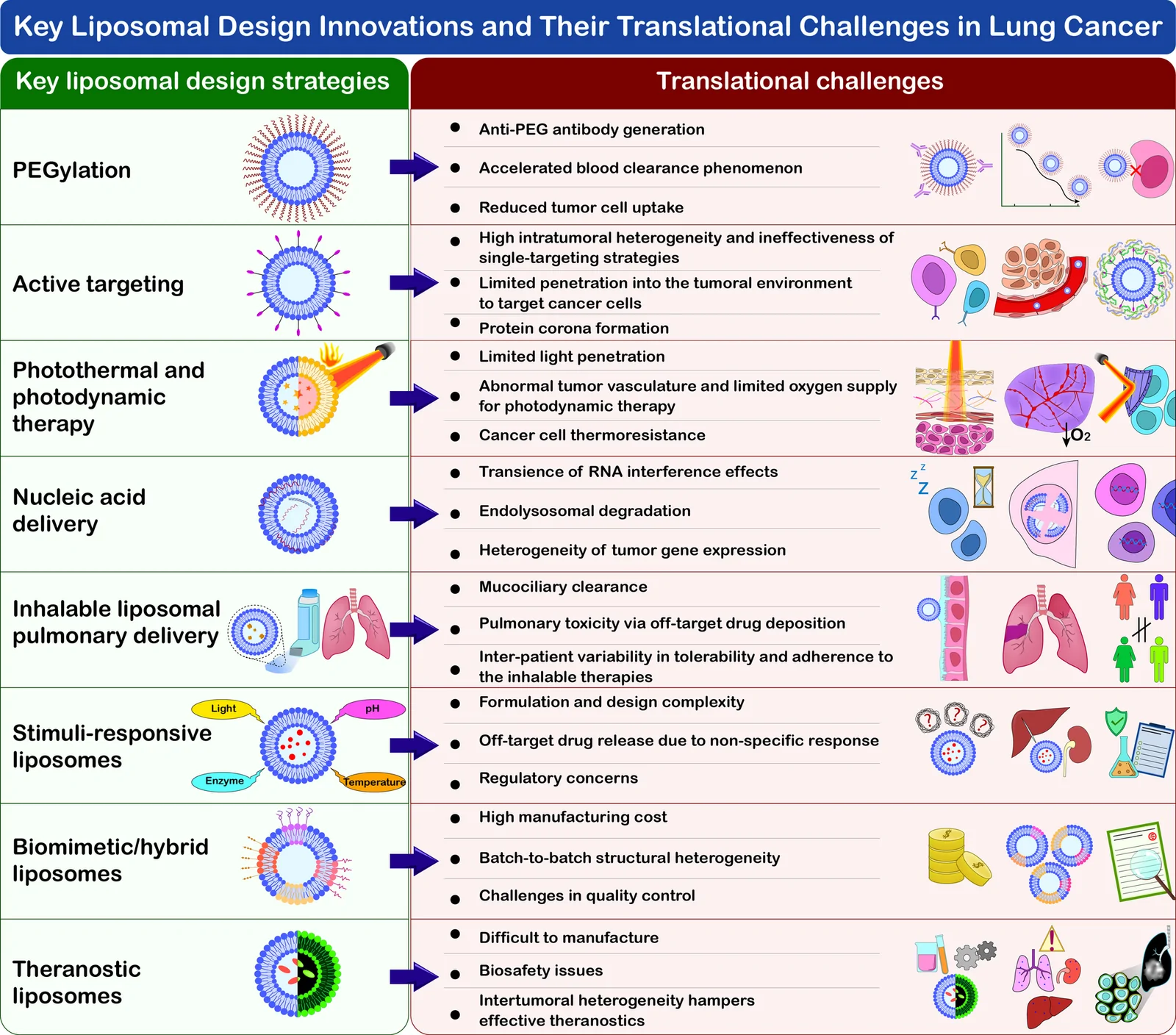
Integrative overview of key liposomal design innovations and their corresponding translational challenges

Despite the extensive body of preclinical evidence depicting the potency of liposome-based oncotherapy in lung cancer, several challenges remain on the path toward clinical translation and future applicability of liposomes (Fig. 17), which lie in the inherent characteristics of liposomal platforms and lung cancer’s intricate pathophysiology, and barriers that arise when these two systems interact:

(i) Misalignment between research directions and remaining challenges.

Failure to address the challenges in nanomedicine clinical translation, paired with a scarcity of challenge-solution-oriented study design frameworks that can bridge the gaps between preclinical and clinical stages, has delayed clinical translatability and overshadowed the effectiveness of clinical nano-oncology. Elucidating fundamental principles governing structural optimizations, factors affecting EPR variability, and nano-bio interactions can increase the reliability, predictability, and reproducibility of preclinical data. Additionally, investigating the practical solutions to overcome high intratumoral heterogeneity, efficient active targeting strategies, and a head-to-head comparison of liposomes with other nanoplatforms can underpin a smoother trajectory to the clinical stage.

(ii) Developmental and translational challenges of advanced technologies.

Multifunctional, smart, biomimetic, and theranostic strategies are in their nascent stage and have a long road ahead. Again, focus must be directed toward pursuing a problem-solving strategy in the design of advanced nanoplatforms to hasten the translatability, reduce the high production costs of advanced platforms, and empower clinical nanotechnology by integrating these cutting-edge insights, which are in need of extensive multidisciplinary collaborations. The capability of liposomes as efficient carriers for advanced oncotherapy should also be compared to other NDDSs to identify the optimal platform or to combine them to enhance the multifunctionality.

(iii) Bottlenecks regarding large-scale production and stockpiling.

Suboptimal storability and shelf life of liposomes hamper the large-scale translation and fabrication [105]. Lipid oxidation/hydrolysis can negatively affect liposomal shelf life [622]. By harnessing and advancing strategies such as using cryoprotectants during freeze-drying [623], leveraging antioxidants to prevent oxidative damage, applying electrostatic and steric stabilization to prevent aggregation [624], modulating lipid composition [625], and utilizing proliposomes [626–628], researchers can prolong the stability of liposomal formulations. Moreover, with the advancement of post-processing technologies [629] and the selection of the best industrial-scale fabrication methods [85], researchers and the pharmaceutical industry are able to cost-effectively fabricate liposomes in large-scale amounts with desired properties, which will streamline clinical translation.

(iv) Incongruence between preclinical and human models.

The imperfection of preclinical models, both in inadequacy in reflecting the intertwined heterogeneity of human cancers and complexities in conducting and maintaining animal models, dictates the establishment and advancement of novel experimental settings, such as patient-derived organoid models that can preserve the host tumoral architecture and heterogeneity, or organ-on-chip models that can harbor a TME inside [630].

Moving forward, future research must focus on devising definitive solutions to the abovementioned challenges. Moreover, it is advised to advance the current perspective of liposomal design, moving beyond conventional formulations and monotherapy toward rationally designed, barrier-responsive, and multifunctional liposomes. By fully leveraging the potential of nanotechnology and integrating existing treatment strategies, such innovations hold promise for improving therapeutic outcomes and addressing the persistent challenges of lung cancer. Prioritizing clinical translatability as a primary objective during the initial study design can increase the likelihood of achieving applicable results. Moreover, focusing on personalized nano-oncology to tackle inter- and intra-patient heterogeneity may pave the way for a more tangible lung cancer therapy.

## Data Availability

No datasets were generated or analysed during the current study.
